# Survival Disparities among Cancer Patients Based on Mobility Patterns: A Population-Based Study

**DOI:** 10.34133/hds.0198

**Published:** 2024-11-05

**Authors:** Fengyu Wen, Yike Zhang, Chao Yang, Pengfei Li, Qing Wang, Luxia Zhang

**Affiliations:** ^1^ Institute of Medical Technology, Peking University Health Science Center, Beijing, China.; ^2^ National Institute of Health Data Science at Peking University, Beijing, China.; ^3^ School of Public Health, Cheeloo College of Medicine, Shandong University, Jinan, Shandong, China.; ^4^ National Institute of Health Data Science of China, Shandong University, Jinan, Shandong, China.; ^5^ Renal Division, Department of Medicine, Peking University First Hospital, Peking University Institute of Nephrology, Beijing, China.; ^6^ Research Units of Diagnosis and Treatment of Immune-mediated Kidney Diseases, Chinese Academy of Medical Sciences, Beijing, China.; ^7^ Advanced Institute of Information Technology, Peking University, Hangzhou, China.

## Abstract

**Background:** Cancer is a major health problem worldwide. A growing number of cancer patients travel to hospitals outside their residential cities due to unbalanced medical resources. We aimed to evaluate the association between patterns of patient mobility and survival among patients with cancer. **Methods:** Data of patients hospitalized for cancer between January 2015 and December 2017 were collected from the regional data platform of an eastern coastal province of China. According to the cities of hospitalization and residency, 3 mobility patterns including intra-city, local center, and national center pattern were defined. Patients with intra-city pattern were sequentially matched to patients with the other 2 patterns on demographics, marital status, cancer type, comorbidity, and hospitalization frequency, using propensity score matching. We estimated 5-year survival and the associations between all-cause mortality and patient mobility. **Results:** Among 20,602 cancer patients, there were 17,035 (82.7%) patients with intra-city pattern, 2,974 (14.4%) patients with local center pattern, and 593 (2.9%) patients with national center pattern. Compared to patients with intra-city pattern, higher survival rates were observed in patients with local center pattern [5-year survival rate, 69.3% versus 65.4%; hazard ratio (HR), 0.85; 95% confidence interval (CI), 0.77 to 0.95] and in patients with national center pattern (5-year survival rate, 69.3% versus 64.5%; HR, 0.80; 95% CI, 0.67 to 0.97). **Conclusions:** We found significant survival disparities among different mobility patterns of patients with cancer. Improving the quality of cancer care is crucial, especially for cities with below-average healthcare resources.

## Introduction

Cancer is a major health problem worldwide, accounting for 9.7 million deaths in 2022 globally [1]. In China, there were an estimated 4.8 million new cancer cases and 2.6 million cancer deaths in 2022 [2]. Over the past several decades, substantial efforts have been made to prevent and control cancer in China, including the implementation of screening and educational programs [3]. In 2022, the age-standardized mortality rate decreased compared with that of 2016, while the cancer incidence still increased [2].

Survival disparities were observed among patients with cancer worldwide. For example, as a complex series of comparisons in both high-resource settings and resource-constrained settings, the issue of breast cancer disparities existed [4]. It has long been known that there were many factors driving disparities in cancer survival, such as race and geographic characteristics [5,6]. As commonly known, racial and urban–rural survival disparities can be partially attributed to inequal access to care, resulting in differences in stage at diagnosis and quality of treatment [7,8]. In addition, there is increasing evidence for survival disparities by gender, socioeconomic status, and insurance among patients with cancer [9–12]. When viewed through different lens, interventions in different directions can be evaluated to reduce the gap and improve outcomes for disadvantaged patients with cancer, such as increasing clinical trial enrollment in minority populations [6,8].

Substantial regional variation in financial resources, medical staff, and infrastructures is one of the most challenging issues for cancer care worldwide [13]. To seek better healthcare, a number of patients with cancer choose to travel to hospitals outside their place of living, which is known as patient mobility [14]. Nowadays, patient mobility is becoming a universal phenomenon because of not only uneven distribution of medical resources but also development of globalization and transportation [15]. In China, many factors have contributed to the patient mobility, such as the substantial regional development disparities and the insufficient implementation of gatekeeping mechanism [16–19]. Because of the tiered healthcare system that prioritizes hospitals from a supply-side perspective, lower quality of care offered by primary care facilities also have made patient mobility a common event [20]. According to the National Medical Service and Quality Safety Report 2021, in 2020, the average medical expenditure of trans-provincial inpatient admissions in tertiary hospitals is 50.38% more than that of intra-provincial inpatient admissions, and the in-hospital mortality is 0.22 percentage point lower [16].

Although limited evidence suggested disparities in in-hospital mortality by mobility patterns among patients with major chronic diseases, such as cancers and kidney failure [16,21], much less is known about disparities in all-cause mortality. Meanwhile, it is critically important to evaluate survival disparities among cancer patients with different mobility patterns not only to improve cancer patient outcomes but also for a sound policy-making for health resource allocation. To fill this gap, we aimed to evaluate the association between pattern of patient mobility and survival among patients with cancer based on a big data platform from millions of people.

## Methods

### Study population

**Fig. 1.**
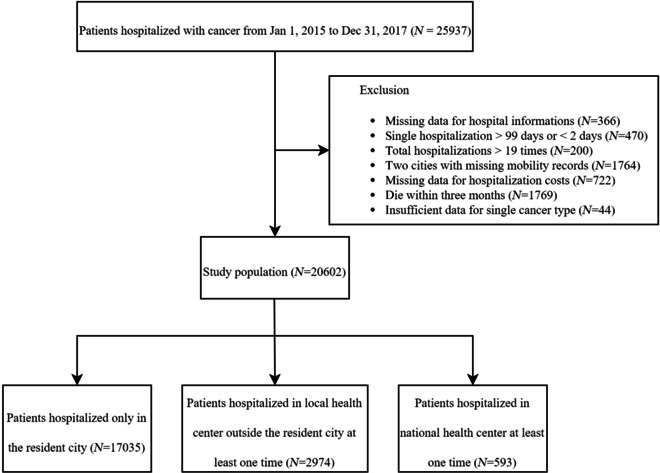
Flow chart of cohort inclusion and exclusion for cancer patients in Shandong province (2015 January 1 to 2017 December 31).

We obtained data from Shandong Multi-Center Healthcare Big Data Platform (SMCHBDP), which is a hybrid system involving more than 5 million residents and integrating multiple health-related sources, such as medical insurance payment systems and death registry [22]. This study included residents of Shandong province, China, who were hospitalized at least once for cancer between 2015 January 1 and 2017 December 31. Twenty-two types of cancer were included, and the corresponding International Classification of Diseases, Tenth Edition (ICD-10) codes are listed in Table S1. The vital status of the patient and the cause of death from 2015 January 1 to 2020 December 31 were derived from SMCHBDP. Of the 25,937 patients eligible for inclusion, those who died within 3 months after their first hospitalization during the study period (*n* = 1,769), those with missing data for essential variables (*n* = 1,132), those with single hospitalization length more than 99 days or less than 2 days (*n* = 470), those hospitalized more than 19 times (*n* = 200), and those in Rizhao and Laiwu where all records of hospitalization outside the city were missing (*n* = 1,764) were excluded, as shown in Fig. 1. Matching variables included age, sex, first hospitalization year, residential city, marital status, cancer types, comorbidities including hypertension, diabetes, heart disease, osteoporosis, and cerebral disease, as well as hospitalization frequency, which was defined as the total number of hospital admissions occurring during the study period [23–26]. The detailed definition of each matching variable can be seen in Table S2.

### Defining mobility patterns

We defined the mobility pattern of a patient based on the locations of the cities where they were hospitalized for cancer and their resident cities. Three mobility patterns were defined: (a) national center pattern—patients who were hospitalized at least once in national medical center cities outside of Shandong Province, primarily in Beijing and Shanghai; (b) local center pattern—patients who were hospitalized at least once in cities within Shandong Province other than their residential city, with a considerable number being treated in Jinan, the provincial capital; (c) intra-city pattern—patients who were hospitalized within their residential city.

### Survival outcomes

The survival outcome was overall 5-year survival. The study starting time was set to be the first hospitalization during the study period. Survival time was defined as the number of months between the study starting time and either the date of death from any cause or the end of the observation period. Patients alive at the end of the observation period were censored. We excluded the patients who died within 3 months after the study starting time to reduce the bias due to possible insufficient cancer-related treatment [12]. In this study, an at least 3-year follow-up period was ensured for each patient. Among the 20,602 included patients with cancer, 6,597 (32.0%) deaths were observed. The median follow-up was 48 months.

### Statistical analysis

In descriptive analysis, out-of-pocket costs and hospitalization frequency were presented as mean. Differences in costs and hospitalization frequency were analyzed using the Wilcoxon rank sum test. Other variables were set as categorical, which were expressed as counts and percentages and evaluated by chi-square test between the pattern groups.

As there were 3 mobility patterns, we conducted 2 sequential matching procedures in our study [11]. We chose intra-city pattern as the reference so that other 2 mobility patterns with fewer patients were relatively constant and representative of the cancer patients seeking healthcare outside residential city. We sequentially added the 5 sets matching variables to the propensity score matching (a) demographics including age, sex, first hospitalization year, residential city (demographic matched); (b) demographics, plus marital status (marriage matched); (c) demographics, marital status, plus cancer types (cancer matched); (d) demographics, marital status, cancer types, plus comorbidities including hypertension, diabetes, heart disease, osteoporosis, and cerebral disease (comorbidity matched); (e) demographics, marital status, cancer types, comorbidities, plus hospitalization frequencies (frequency matched). The propensity score matching was performed using the nearest neighbor search strategy with a caliper of 0.1 [27]. We estimated the propensity scores using a multivariate logistic regression model separately for each set of matching variables. We used exact balance for residential city and sex in matching. One and 2 patients with intra-city pattern were matched to each patient with local center and national center pattern, respectively, due to the relatively small number of patients seeking care in national medical center. Cox proportional hazards model was used to generate the hazard ratios (HRs) of 5-year all-cause mortality for patients with 2 mobility patterns compared to intra-city pattern separately. The overall survival was estimated using Kaplan–Meier method. A standardized mean difference (SMD) below 0.1 after matching was considered to indicate a good balance [28]. Statistical significance was determined based on the 2-sided *P* value <0.05. All analyses were performed in R-4.1.2.

We conducted subgroup analyses for cancer types and cities with different healthcare resource levels. The first 5 cancers (lung, colorectum, stomach, breast, and thyroid) with the largest number of patients in the dataset were classified as common cancers. Other cancers were classified as uncommon cancers and were listed in Table S3. As a proxy indicator for healthcare resources [29], hospital bed per capita was used to define above-average and below-average healthcare resource subgroups. Cities with more hospital beds per capita than the average of all cities in Shandong province were classified as above-average, while cities with fewer hospital beds per capita were classified as below-average. The detailed definition of each subgroup is listed in Table S3. To investigate the impact of patients who have been hospitalized before the study period, we performed a sensitivity analysis by restricting the study population to patients with no hospitalization records within the first 6 months of the study period (2015 January 1 to 2015 May 31).

## Results

### Patient characteristics and overall matching results

Primary characteristics of patients stratified by mobility patterns are shown in Table 1. A total of 20,602 residents in Shandong Province hospitalized for cancer from January 2015 to December 2017 were included. Among them, there were 17,035 (82.7%) patients with intra-city pattern, 2,974 (14.4%) patients with local center pattern, and 593(2.9%) patients with national center pattern. Eighty-eight percent of patients with local center pattern were hospitalized at least once in Jinan, the provincial capital of Shandong. Seventy-four percent of patients with national center pattern were hospitalized at least once in Beijing or Shanghai, and 22% in other provincial capitals or municipalities in China, such as Tianjin, Chengdu, and Hangzhou.

**Table 1.** Primary characteristics of patients with difference mobility patterns

Variables	Overall	Intra-city	Local center	*P* value^a^	National center	*P* value^b^
*N*	20,602	17,035	2,974		593	
Age (%)				<0.001		<0.001
<40	1,285 (6.2)	950 (5.6)	258 (8.7)		77 (13.0)	
40–50	2,550 (12.4)	1,950 (11.4)	461 (15.5)		139 (23.4)	
50–60	4,675 (22.7)	3,795 (22.3)	732 (24.6)		148 (25.0)	
>60	12,092 (58.7)	10,340 (60.7)	1,523 (51.2)		229 (38.6)	
Sex (%)				<0.001		0.004
Male	10,983 (53.3)	8,898 (52.2)	1,739 (58.5)		346 (58.3)	
Female	9,619 (46.7)	8,137 (47.8)	1,235 (41.5)		247 (41.7)	
Healthcare resources (%)				<0.001		<0.001
Above-average	14,576 (70.8)	13,118 (77.0)	1,126 (37.9)		332 (56.0)	
Below-average	6,026 (29.2)	3,917 (23.0)	1,848 (62.1)		261 (44.0)	
Cancer type (%)				0.475		<0.001
Five most common	13,487 (65.5)	11,189 (65.7)	1,974 (66.4)		324 (54.6)	
Uncommon	7,115 (34.5)	5,846 (34.3)	1,000 (33.6)		269 (45.4)	
Marital status (%)				<0.001		<0.001
Married	12,864 (62.4)	10,864 (63.8)	1,628 (54.7)		372 (62.7)	
Single	306 (1.5)	224 (1.3)	58 (2.0)		24 (4.0)	
Widowed/divorced	607 (2.9)	549 (3.2)	55 (1.8)		3 (0.5)	
Unknown	6,825 (33.1)	5,398 (31.7)	1,233 (41.5)		194 (32.7)	
Comorbidity (%)				<0.001		0.336
No	15,238 (74.0)	12,835 (75.3)	1,967 (66.1)		436 (73.5)	
Yes	5,364 (26.0)	4,200 (24.7)	1,007 (33.9)		157 (26.5)	
Hospitalization frequency	3.5	3.4	4.0	<0.001	3.8	0.021
Out-of-pocket costs ($)	1,202.7	995.9	2,183.0	<0.001	2,228.4	<0.001

^a^ The *P* values were calculated between intra-city and local center patterns.

^b^ The *P* values were calculated between intra-city and national center patterns. Intra-city indicated patients who were never hospitalized outside the residential city. Local center indicated patients who were never hospitalized outside the Shandong province but were hospitalized outside the residential city at least once. National center indicated patients who were hospitalized outside the Shandong province at least once. Five most common cancers included lung, colorectum, stomach, breast, and thyroid cancer. Uncommon cancers were defined as other cancers except 5 common cancers. Above-average and below-average healthcare resource indicated cities with hospital beds per capita more and less than average level, respectively. Comorbidity includes hypertension, diabetes, heart disease, osteoporosis, and cerebral disease.

As shown in Table 1, compared to those with 2 other patterns, patients with intra-city pattern were older, had lower hospitalization frequency, and cost less. No significant difference was observed in comorbidity. Median follow-up time was 3.9 years [interquartile range (IQR), 2.2 to 5.0] for patients with intra-city pattern, 4.3 years (IQR, 3.1 to 5.2) for local center pattern, and 4.1 years (IQR, 2.7 to 5.0) for national pattern. Patients with the 5 most common cancers, including lung, colorectum, stomach, breast, and thyroid cancer, were less likely to travel to local medical center, compared to patients with the relatively uncommon cancers. The proportions of patients with national and local center patterns residing in cities with above-average healthcare resources were significantly lower than those in cities with below-average healthcare resources.

Detailed characteristics are listed in Table S2. The sequential matches substantially increased the similarity between patients with different patterns, as the SMD was lower than 0.1 for most covariates, which was summarized in Tables S4 to S8.

### Survival disparity in the overall population

**Fig. 2.**
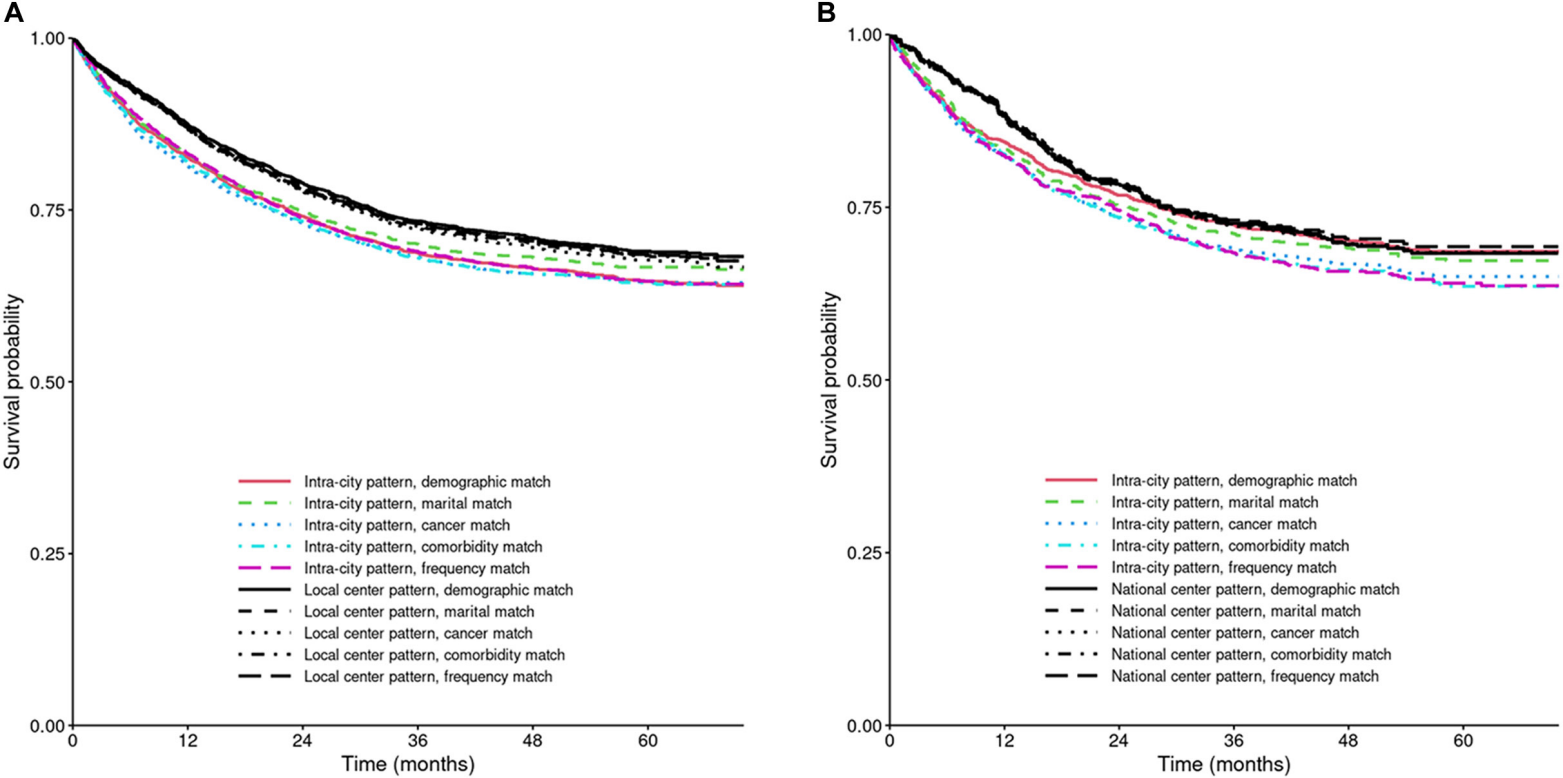
Overall survival curves for sequentially matched cancer patients. (A) Intra-city pattern versus local center pattern. (B) Intra-city pattern versus national center pattern.

Survival curves of sequentially matched patients are shown in Fig. 2. The 5-year survival, absolute survival rate difference between each matched set of 2 compared patterns, and HR of the mobility patterns are listed in Table 2. Marital status accounted for the largest overall 5-year survival disparities between intra-city and local center patterns, amounting to 1.9 percentage point (pp.) changes in absolute survival difference, followed by cancer type, amounting to 0.8 pp. changes, as shown in Table 2. Similarly, cancer type accounted for the largest overall 5-year survival disparities between intra-city and national center patterns, amounting to 2.3 pp. changes, followed by marital status, amounting to 1.2 pp. changes.

**Table 2.** Five-year survival for sequentially matched patients and HR of all-cause mortality risk for local and national center patterns versus intra-city pattern

Comparison between intra-city pattern and local center pattern					
	Demographic matched	Marriage matched	Cancer matched	Morbidity matched	Frequency matched
5-year all-cause survival (%)					
Intra-city	65.6	67.2	65.3	65.2	**65.4**
Local center	69.8	69.5	68.4	69.0	**69.3**
Difference in survival (pp., reference: intra-city)	4.2	2.3	3.1	3.8	**3.9**
Difference explained (pp.)^a^		1.9	0.8	0.7	**0.1**
HR (95% CI, reference: intra-city)	0.83 (0.75–0.92)	0.89 (0.80–0.99)	0.86 (0.77–0.95)	0.84 (0.75–0.93)	**0.85 (0.77–0.95)**
*P* value	<0.01	0.03	<0.01	<0.01	**<0.01**
Comparison between intra-city pattern and national center pattern					
	Demographic matched	Marriage matched	Cancer matched	Morbidity matched	Frequency matched
5-year all-cause survival (%)					
Intra-city	68.8	67.7	65.5	64.6	**64.5**
National center	68.3	68.4	68.5	68.4	**69.3**
Difference in survival (pp., reference: intra-city)	−0.5	0.7	3.0	3.8	**4.8**
Difference explained (pp.)^a^		1.2	2.3	0.8	**1.0**
HR (95% CI, reference: intra-city)	0.98 (0.82–1.17)	0.94 (0.78–1.12)	0.86 (0.71–1.03)	0.83 (0.69–1.00)	**0.80 (0.67–0.97)**
*P* value	0.82	0.47	0.10	0.04	**0.02**

^a^ The survival difference explained by a set of matching variables was calculate as the absolute change in survival difference before and after adjusting for the corresponding set of variables. Demographic variables included age, sex, first hospitalization year, residential city. Marriage variables included demographic variables and marital status. Cancer variables included marriage variables and cancer types. Comorbidity variables included cancer variables and comorbidities including hypertension, diabetes, heart disease, osteoporosis, cerebral disease. Frequency variables included comorbidity variables and hospitalization frequencies (shown in bold). Intra-city indicated patients who were never hospitalized outside the residential city. Local center indicated patients who were never hospitalized outside the Shandong province but were hospitalized outside the residential city at least once. National center indicated patients who were hospitalized outside the Shandong province at least once.

In total, significant survival disparities were observed for both comparisons between intra-city and local center patterns [5-year survival rate, 65.4% versus 69.3%; HR, 0.85; 95% confidence interval (CI), 0.77 to 0.95], and between intra-city and national center patterns (5-year survival rate, 64.5% versus 69.3%; HR, 0.80; 95% CI, 0.67 to 0.97) after all variables were matched. Patients with intra-city pattern had lower survival than patients with other 2 patterns, as shown in Fig. 2. Results were consistent in the performed sensitivity analysis, as listed in Table S9.

### Survival disparity in cancer type and health resource level subgroups

In subgroup analysis, the 5-year survival rates, absolute survival rate difference between each matched set of patients with 2 patterns, and HR of the mobility patterns are listed in Table 3. For the subgroup of the 5 most common cancers, a significantly higher survival was found in local center pattern than intra-city pattern after all variables were matched (5-year survival rate, 67.5% versus 73.4%; HR, 0.78; 95% CI, 0.67 to 0.90). There was no significant survival difference observed between intra-city and national center patterns. For the subgroup of relatively uncommon cancer, there was no significant survival difference between intra-city and local center patterns after final matching. Patients with national center pattern had a significantly higher survival than those with intra-city pattern after all variables were matched (5-year survival rate, 52.5% versus 62.6%; HR, 0.72; 95% CI, 0.55 to 0.94).

**Table 3.** Five-year survival for frequency-matched matched patients and HR of all-cause mortality risk for local and national center patterns versus intra-city pattern by healthcare resource and cancer type

Comparison between intra-city pattern and local center pattern					
	Five most common cancers	Uncommon cancers	Above-average healthcare resource	Below-average healthcare resources
5-year all-cause survival (%)				
Intra-city	67.5	59.8	65.8	64.9
Local center	73.4	62.2	69.2	68.3
Difference in survival (pp., reference: intra-city)	5.9	2.4	3.4	3.4
HR (95% CI, reference: intra-city)	0.78 (0.67–0.90)	0.88 (0.75–1.03)	0.87 (0.74–1.01)	0.85 (0.74–0.99)
*P* value	<0.01	0.10	0.07	0.03
Comparison between intra-city pattern and national center pattern					
	Five most common cancers	Uncommon cancers	Above-average healthcare resource	Below-average healthcare resources
5-year all-cause survival (%)				
Intra-city	71.9	52.5	65.3	66.5
National center	73.1	62.6	71.6	66.9
Difference in survival (pp., reference: intra-city)	1.2	10.1	6.3	0.4
HR (95% CI, reference: intra-city)	0.88 (0.67–1.16)	0.72 (0.55–0.94)	0.75 (0.58–0.96)	0.94 (0.70–1.25)
*P* value	0.37	0.02	0.03	0.67

In the frequency-matching process, which was the final step in the sequential matching procedure, all matching variables were included: age, sex, year of first hospitalization, residential city, marital status, cancer types, comorbidities (including hypertension, diabetes, heart disease, osteoporosis, and cerebral disease), and hospitalization frequencies. Intra-city indicated patients who were never hospitalized outside the residential city. Local center indicated patients who were never hospitalized outside the Shandong province but were hospitalized outside the residential city at least once. National center indicated patients who were hospitalized outside the Shandong province at least once. Five most common cancers included lung, colorectum, stomach, breast, and thyroid cancer. Uncommon cancers were defined as other cancers except 5 common cancers. Above-average and below-average healthcare resource groups indicated cities with hospital beds per capita more and less than average level of all cities in Shandong province, respectively.

Among patients residing in the cities with above-average healthcare resources, significant survival disparity was observed between intra-city and national center pattern after all variables were matched. A higher 5-year survival was observed in national center pattern (5-year survival rate, 65.3% versus 71.6%; HR, 0.75; 95% CI, 0.58 to 0.96), as listed in Table 3 and Table S10. There was no significant survival disparity observed between intra-city and local medical patterns. Among residents in the cities with below-average healthcare resources, there was also no significant difference in cancer survival between intra-city and national center patterns after final matching. Patients with local center pattern had a statistically significant higher survival than those with intra-city pattern after all variables were matched (5-year survival rate, 64.9% versus 68.3%; HR, 0.85; 95% CI, 0.74 to 0.99). The detailed matching properties are listed in Tables S5 to S8. The matching quality was adequate.

## Discussion

To the best of our knowledge, this is the first study that evaluated the survival disparities for patients with cancer choosing their healthcare provider with different patterns, using a multi-source big data platform of northern China. In this study, we found significant survival disparities across different mobility pattern groups using the sequential matching method. Overall, patients with intra-city pattern had lower survival rates than those with local center and national center patterns after 5 sets of variables were matched adequately, which was in line with the previous studies [16,21].

In recent years, there has been a growing number of studies on disparities in cancer survival. Despite different perspectives, they shared a common goal of improving the outcomes of disadvantaged groups and reducing survival disparities [6,10,24,25,30]. However, previous studies focused on survival disparities by a single factor. For example, a study found persistent disparities in breast cancer survival between black and white women [11]. Another study found that 5-year survivals of all cancers were higher in rural than in urban areas in Shanghai, China [8]. Given the complexity of the factors associated with cancer survival, it can be challenging to intervene on a single factor. Instead, patient mobility is determined by a complex interplay of multiple factors including allocation of healthcare resources and patient characteristics, such as age and socioeconomic status [31]. Over the past few years, patient mobility has gained increasing attention globally. In addition, for a patient with cancer, the choice of mobility pattern is a crucial step in the treatment process. Better outcomes and higher survival rates are the ultimate goals of treatment and also the primary starting point for policy-making [32–34]. Although there have been studies analyzing the association between patient mobility and in-hospital mortality, there is a lack of evidence on the association between patient mobility and all-cause mortality, partly because of the difficulty in obtaining follow-up data [15]. Therefore, focusing on mobility patterns of hospitalized patients with cancer, our study provided a new perspective on survival disparities. The findings of this study could provide clues for a better understanding of the differences in survival and serve as evidence for the rational allocation of healthcare resources to effectively improve survival rates for both “vulnerable” regions and patients. Moreover, survival disparities by mobility patterns offer new insights in policy-level incentives to overcome barriers to optimal treatment, including establishing an appropriate reimbursement rate for out-of-province patients and setting up cancer centers.

After matching several variables considered in previous studies [5,6], we still found higher survival rates for patients with center mobility patters, compared to patients with intra-city pattern. There might be multiple explanations for the survival disparities. First, previous research indicated a superior safety and effectiveness of the overall care of patients in top-ranked cancer hospitals compared with affiliate hospitals. The differences in care may include both nonsurgical and surgical components [35]. Second, higher hospital financial status in medical center has been demonstrated to influence the adoption of advanced technologies in cancer treatment [36].Third, the quality of healthcare services was an influential determinant of patient satisfaction, which had a positive impact on cancer survival as well [37]. Furthermore, several patient characteristics related to better capacity for mobility, such as higher socioeconomic status and better family support, were associated with higher survival as well [26,31,38,39].

It is worth noting that 2 center mobility patterns are 2-edged swords. Although higher survival rates were found among 2 center mobility patterns, higher out-of-pocket costs of hospitalization were also found. Furthermore, hospitalizations outside the residential city can result in additional burdens such as transportation costs, loss of productivity, and psychological burden imposed on patients and their families [40]. Outcomes can be adversely impacted by the financial burden resulting from direct or indirect costs, especially for patients with cancer, which is known as financial toxicity [41,42]. Although searching for the optimal mobility pattern is beyond the scope of this study, it is unreasonable to encourage people to be hospitalized only in medical centers.

The subgroup analyses provided important insights into the impact of healthcare resources and cancer types on survival disparities, which could serve as valuable evidence for policy-making. Regional factors also motivated patient mobility, such as seeking high-quality medical resources [43]. Worldwide, including China, medical resources tend to be skewed toward areas with higher economic status [29,44]. In this study, patients in cities with suboptimal healthcare resources tended to seek treatment outside of their residential cities. Compared with common cancers, the treatment of uncommon cancers demands higher expertise from medical personnel and has considerable unmet medical need, especially in smaller countries [45,46]. The results of the subgroup analysis were highly consistent with those of the complete cohort, showing that the survival rate of patients with both national and local center pattern was higher. However, a significant difference was observed only between intra-city and local center patterns within the common cancer and below-average resource subgroups and between intra-city and national center patterns within the uncommon cancer and above-average resource subgroups. While the local medical center had a good foundation for the treatment of common cancers, there was still room for improvement in the treatment of uncommon cancers compared with the national medical center. The quality of cancer care in cities with above-average medical resources was advanced within the province, but not comparable with national medical centers. The quality of cancer care in cities with medical below-average resources fell short of regional medical centers and needed to be strengthened. No significant difference was observed in the survival between national center and intra-city patterns in the subgroups with common cancers and below-average resources. There were 3 possible explanations. First, some factors that impact survival were not included in the study due to data availability. For instance, patients with national center pattern might have more advanced stages of cancer or more challenging cases than those with the other 2 patterns, which could counterbalance the benefits of better care. Second, traveling to national centers could result in less consistent follow-up treatment and monitoring compared to receiving care at closer local centers. Third, patients traveling to national centers may face higher psychological and financial burdens, which can negatively affect their overall health and survival [41,42,47]. Taken together, these lines of evidence suggested that more efforts were needed to be tailored for cities with below-average healthcare resources and uncommon cancers to control survival disparities.

A strength of our study is the use of sequential matching strategy. As patients hospitalized in the residential city accounted for the majority of the study cohort, the results from such an imbalanced population using a model-based method may be limited [11]. Another strength is the large population extracted from a hybrid data platform, which covered a wide range of variables such as survival time, marital status, and costs. The extensive data platform allowed us to focus on the survival, which has rarely been studied in similar literature, and to eliminate the potential confounding effects as much as possible. The large cohort also ensured the very close matches. There are some limitations in this study that could be addressed in future research. First, although we controlled for a wide variety of patient characteristics, the primary findings still might be subject to residual confounding. Factors not accounted for in this study that could contribute to the choice of mobility pattern and unexplained survival disparities included cancer stage and socioeconomic status [11,24,25]. However, the marital status included in this study was associated with socioeconomic status and could serve as a proxy, partially adjusting for it [48]. Second, the costs in this study were only those in the medical records. For patients hospitalized outside the residential city, there were many indirect costs for patients and their families that were not estimated, such as travel costs and loss of productivity [47,49], as well as psychological burden [50]. Third, this study was restricted to hospitalized patients and could not analyze the mobility for outpatients [51]. Fourth, due to the lack of data on patients’ diagnosis, survival times might be underestimated. However, our sensitivity analysis demonstrated its limited impact on our results. Although patients who visited local or national centers before the study period might have been misclassified to intra-city pattern, this misclassification led to underestimation of survival disparity and would not affect our main conclusions. To overcome these limitations, future research needs more detailed data on the preference of mobility patterns and survival outcomes, including factors such as cancer stage and date of diagnosis. This will help to better understand and address survival disparities, and ultimately promote the development of comprehensive strategies to reduce medical resource imbalances and improve patient outcomes.

## Conclusion

In conclusion, survival disparities existed among different mobility patterns of hospitalized patients with cancer. After the adjustment for several confounding variables, patients who never hospitalized outside the residential city had lower 5-year survival rates and lower out-of-pocket costs compared with those with trans-city hospitalizations. This study highlights the need to improve the cancer care, especially for cities with below-average healthcare resources. Future studies are needed to fully understand and control the survival disparities using more detailed data.

## Data Availability

The data that support the findings of this study are available from the corresponding author upon reasonable request.

## References

[1] Bray F, Laversanne M, Sung H, Ferlay J, Siegel RL, Soerjomataram I, Jemal A. Global cancer statistics 2022: GLOBOCAN estimates of incidence and mortality worldwide for 36 cancers in 185 countries. CA Cancer J Clin. 2024;74(3):229–263. 38572751 10.3322/caac.21834

[2] Han B, Zheng R, Zeng H, Wang S, Sun K, Chen R, Li L, Wei W, He J. Cancer incidence and mortality in China, 2022. J Natl Cancer Cent. 2024;4(1):47–53. 39036382 10.1016/j.jncc.2024.01.006 PMC11256708

[3] Cao M, Li H, Sun D, He S, Yu Y, Li J, Chen H, Shi J, Ren J, Li N, et al. Cancer screening in China: The current status, challenges, and suggestions. Cancer Lett. 2021;506:120–127. 33684533 10.1016/j.canlet.2021.02.017

[4] Reeder-Hayes KE, Anderson BO. Breast cancer disparities at home and abroad: A review of the challenges and opportunities for system-level change. Clin Cancer Res. 2017;23(11):2655–2664. 28572260 10.1158/1078-0432.CCR-16-2630 PMC5499686

[5] Silber JH, Rosenbaum PR, Ross RN, Niknam BA, Ludwig JM, Wang W, Clark AS, Fox KR, Wang M, Even-Shoshan O, et al. Racial disparities in colon cancer survival: A matched cohort study. Ann Intern Med. 2014;161(12):845–854. 25506853 10.7326/M14-0900

[6] Bhatia S, Landier W, Paskett ED, Peters KB, Merrill JK, Phillips J, Osarogiagbon RU. Rural–Urban disparities in cancer outcomes: Opportunities for future research. J Natl Cancer Inst. 2022;114(7):940–952. 35148389 10.1093/jnci/djac030 PMC9275775

[7] Meza R, Meernik C, Jeon J, Cote ML. Lung cancer incidence trends by gender, race and histology in the United States, 1973–2010. PLOS ONE. 2015;10(3): Article e0121323. 25822850 10.1371/journal.pone.0121323 PMC4379166

[8] Li X, Deng Y, Tang W, Sun Q, Chen Y, Yang C, Yan B, Wang Y, Wang J, Wang S, et al. Urban-rural disparity in cancer incidence, mortality, and survivals in Shanghai, China, during 2002 and 2015. Front Oncol. 2018;8:579. 30560091 10.3389/fonc.2018.00579 PMC6287035

[9] Tran PN, Taylor TH, Klempner SJ, Zell JA. The impact of gender, race, socioeconomic status, and treatment on outcomes in esophageal cancer: A population-based analysis. J Carcinog. 2017;16:3. 28974922 10.4103/jcar.JCar_4_17 PMC5615860

[10] Brouwer AF, Engle JM, Jeon J, Meza R. Sociodemographic survival disparities for lung cancer in the United States, 2000-2016. J Natl Cancer Inst. 2022;114(11):1492–1500. 35866998 10.1093/jnci/djac144 PMC9664170

[11] Silber JH, Rosenbaum PR, Clark AS, Giantonio BJ, Ross RN, Teng Y, Wang M, Niknam BA, Ludwig JM, Wang W, et al. Characteristics associated with differences in survival among black and white women with breast cancer. JAMA. 2013;310(4):389–397. 23917289 10.1001/jama.2013.8272

[12] Lai Y, Wang C, Civan JM, Palazzo JP, Ye Z, Hyslop T, Lin J, Myers RE, Li B, Jiang B, et al. Effects of cancer stage and treatment differences on racial disparities in survival from colon cancer: A United States population-based study. Gastroenterology. 2016;150(5):1135–1146. 26836586 10.1053/j.gastro.2016.01.030 PMC4842115

[13] Zhang Y, Wang Q, Jiang T, Wang J. Equity and efficiency of primary health care resource allocation in mainland China. Int J Equity Health. 2018;17(1):140. 30208890 10.1186/s12939-018-0851-8 PMC6134520

[14] Yan X, Shan L, He S, Zhang J. Cross-city patient mobility and healthcare equity and efficiency: Evidence from Hefei, China. Travel Behav Soc. 2022;28:1–12.

[15] Lunt N, Mannion R. Patient mobility in the global marketplace: A multidisciplinary perspective. Int J Health Policy Manag. 2014;2(4):155–157. 24847479 10.15171/ijhpm.2014.47 PMC4025090

[16] National Health Commission. 2021 National Medical Service and Quality Safety Report: Science and Technology Literature Publishing House; 2022.

[17] Xu J, Mills A. Challenges for gatekeeping: A qualitative systems analysis of a pilot in rural China. Int J Equity Health. 2017;16(1):106. 28666445 10.1186/s12939-017-0593-z PMC5493841

[18] Liu C, Wang Y. Research status and prospect of cross-regional medical treatment in China based on bibliometric analysis. Chin Gen Pract. 2024;27(12):1525–1532.

[19] Wang X, Nie X. The uneven distribution of medical resources for severe diseases in China: An analysis of the disparity in inter-city patient mobility. Appl Geogr. 2024;165: Article 103226.

[20] Zhang A, Nikoloski Z, Albala SA, Yip W, Xu J, Mossialos E. Patient choice of health care providers in China: Primary care facilities versus hospitals. Health Syst Reform. 2020;6(1): Article e1846844. 33314985 10.1080/23288604.2020.1846844

[21] Ao Y, Yang C, Li P, Wang F, Peng S, Wang H-Y, Wang J, Zhao M-H, Zhang L, Yuan Y, et al. Cost-effectiveness of medical migration for chronic kidney disease: A national cross-sectional study in China. BMC Health Serv Res. 2022;22(1):912. 35831849 10.1186/s12913-022-08266-x PMC9281168

[22] Du W-Y, Yin C-N, Wang H-T, Li Z-W, Wang W-J, Xue F-Z, Zhao L, Cao W-C, Cheeloo EcoHealth Consortium (CLEC). Infectious diseases among elderly persons: Results from a population-based observational study in Shandong province, China, 2013-2017. J Glob Health. 2021;11: Article 08010. 35003717 10.7189/jogh.11.08010 PMC8710039

[23] Ding J, Yang C, Wang Y, Li P, Wang F, Kang Y, Wang H, Liang Z, Zhang J, Han P, et al. Influential factors of intercity patient mobility and its network structure in China. Cities. 2023;132: Article 103975.

[24] Liu JC, Egleston BL, Blackman E, Ragin C. Racial survival disparities in head and neck cancer clinical trials. J Natl Cancer Inst. 2022;115(3):288–294. 10.1093/jnci/djac219 PMC9996207 36477855

[25] Valeri L, Chen JT, Garcia-Albeniz X, Krieger N, VanderWeele TJ, Coull BA. The role of stage at diagnosis in colorectal cancer black-white survival disparities: A counterfactual causal inference approach. Cancer Epidemiol Biomarkers Prev. 2016;25(1):83–89. 26503034 10.1158/1055-9965.EPI-15-0456 PMC4713332

[26] Muhamad M, Afshari M, Kazilan F. Family support in cancer survivorship. Asian Pac J Cancer Prev. 2011;12(6):1389–1397. 22126470

[27] Ho DE, Imai K, King G, Stuart EA. MatchIt: Nonparametric preprocessing for parametric causal inference. J Stat Softw. 2011;42(8):1–28.

[28] Pescarini JM, Williamson E, Nery JS, Ramond A, Ichihara MY, Fiaccone RL, Penna MLF, Smeeth L, Rodrigues LC, Penna GO, et al. Effect of a conditional cash transfer programme on leprosy treatment adherence and cure in patients from the nationwide 100 Million Brazilian Cohort: A quasi-experimental study. Lancet Infect Dis. 2020;20(5):618–627. 32066527 10.1016/S1473-3099(19)30624-3 PMC7191267

[29] Pan J, Shallcross D. Geographic distribution of hospital beds throughout China: A county-level econometric analysis. Int J Equity Health. 2016;15(1):179. 27821181 10.1186/s12939-016-0467-9 PMC5100192

[30] Ellis L, Canchola AJ, Spiegel D, Ladabaum U, Haile R, Gomez SL. Racial and ethnic disparities in cancer survival: The contribution of tumor, sociodemographic, institutional, and neighborhood characteristics. J Clin Oncol. 2018;36(1):25–33. 29035642 10.1200/JCO.2017.74.2049 PMC5756323

[31] Victoor A, Delnoij DM, Friele RD, Rademakers JJDJM. Determinants of patient choice of healthcare providers: A scoping review. BMC Health Serv Res. 2012;12:272. 22913549 10.1186/1472-6963-12-272 PMC3502383

[32] Durie BGM. Role of new treatment approaches in defining treatment goals in multiple myeloma – The ultimate goal is extended survival. Cancer Treat Rev. 2010;36(Suppl. 2):S18–S23. 20472184 10.1016/S0305-7372(10)70008-6

[33] Mariotto AB, Noone A-M, Howlader N, Cho H, Keel GE, Garshell J, Woloshin S, Schwartz LM. Cancer survival: An overview of measures, uses, and interpretation. J Natl Cancer Inst Monogr. 2014;2014(49):145–186. 25417231 10.1093/jncimonographs/lgu024 PMC4829054

[34] Tan X, Liu X, Shao H. Healthy China 2030: A vision for health care. Value Health Reg Issues. 2017;12:112–114. 28648308 10.1016/j.vhri.2017.04.001

[35] Boffa DJ, Mallin K, Herrin J, Resio B, Salazar MC, Palis B, Facktor M, McCabe R, Nelson H, Shulman LN. Survival after cancer treatment at Top-Ranked US Cancer Hospitals vs Affiliates of Top-Ranked Cancer Hospitals. JAMA Netw Open. 2020;3(5): Article e203942. 32453382 10.1001/jamanetworkopen.2020.3942 PMC7251445

[36] Wright JD, Tergas AI, Hou JY, Burke WM, Chen L, Hu JC, Neugut AI, Ananth CV, Hershman DL. Effect of regional hospital competition and hospital financial status on the use of robotic-assisted surgery. JAMA Surg. 2016;151(7):612–620. 26886156 10.1001/jamasurg.2015.5508

[37] Cheng S-H, Yang M-C, Chiang T-L. Patient satisfaction with and recommendation of a hospital: Effects of interpersonal and technical aspects of hospital care. Int J Qual Health Care. 2003;15(4):345–355. 12930050 10.1093/intqhc/mzg045

[38] Yu XQ, O’Connell DL, Gibberd RW, Armstrong BK. Assessing the impact of socio-economic status on cancer survival in New South Wales, Australia 1996–2001. Cancer Causes Control. 2008;19(10):1383–1390. 18704715 10.1007/s10552-008-9210-1

[39] Yuan B, Zhang T, Li J. Family support and transport cost: Understanding health service among older people from the perspective of social-ecological model. Arch Public Health. 2022;80(1):173. 35850919 10.1186/s13690-022-00923-1 PMC9295433

[40] Payne S, Jarrett N, Jeffs D. The impact of travel on cancer patients’ experiences of treatment: A literature review. Eur J Cancer Care. 2000;9(4):197–203. 10.1046/j.1365-2354.2000.00225.x 11829366

[41] Zafar SY, Peppercorn JM, Schrag D, Taylor DH, Goetzinger AM, Zhong X, Abernethy AP. The financial toxicity of cancer treatment: A pilot study assessing out-of-pocket expenses and the insured cancer patient's experience. Oncologist. 2013;18(4):381–390. 23442307 10.1634/theoncologist.2012-0279 PMC3639525

[42] Lentz R, Benson AB III, Kircher S. Financial toxicity in cancer care: Prevalence, causes, consequences, and reduction strategies. J Surg Oncol. 2019;120(1):85–92. 30650186 10.1002/jso.25374

[43] Balia S, Brau R, Marrocu E. Interregional patient mobility in a decentralized healthcare system. Reg Stud. 2018;52(3):388–402.

[44] Zhang T, Xu Y, Ren J, Sun L, Liu C. Inequality in the distribution of health resources and health services in China: Hospitals versus primary care institutions. Int J Equity Health. 2017;16(1):42. 28253876 10.1186/s12939-017-0543-9 PMC5335774

[45] Gatta G, Trama A, Capocaccia R. Epidemiology of rare cancers and inequalities in oncologic outcomes. Eur J Surg Oncol. 2019;45(1):3–11. 29032924 10.1016/j.ejso.2017.08.018

[46] Wang S, Jiang Y, Miao H, Fang Y, Jiang N, Yu Y, Ma P, Tang Q, Cui D, Fang H, et al. Targeting rare tumors: New focus for clinical research in China. EMBO Mol Med. 2023;15(1): Article e16415. 36437781 10.15252/emmm.202216415 PMC9832829

[47] Athanasakis K, Souliotis K, Kyriopoulos EJ, Loukidou E, Kritikou P, Kyriopoulos J. Inequalities in access to cancer treatment: An analysis of cross-regional patient mobility in Greece. Support Care Cancer. 2012;20(3):455–460. 21258947 10.1007/s00520-011-1093-0

[48] Karney BR. Socioeconomic status and intimate relationships. Annu Rev Psychol. 2021;72:391–414. 32886585 10.1146/annurev-psych-051920-013658 PMC8179854

[49] Jordan H, Roderick P, Martin D, Barnett S. Distance, rurality and the need for care: Access to health services in South West England. Int J Health Geogr. 2004;3(1):21. 15456514 10.1186/1476-072X-3-21 PMC524184

[50] Yabroff KR, Warren JL, Knopf K, Davis WW, Brown ML. Estimating patient time costs associated with colorectal cancer care. Med Care. 2005;43(7):640–648. 15970778 10.1097/01.mlr.0000167177.45020.4a

[51] Varkevisser M, van der Geest SA, Schut FT. Assessing hospital competition when prices don't matter to patients: The use of time-elasticities. Int J Health Care Finance Econ. 2010;10(1):43–60. 19662527 10.1007/s10754-009-9070-6

