# Supplementary material for: Survival Disparities among Cancer Patients Based on Mobility Patterns: A Population-Based Study
Source: Health Data Sci. 2024 Nov 5;10:0198. doi: 10.34133/hds.0198 (PMC11535395; doi:10.34133/hds.0198)
Supplement: Supplementary 1 — Tables S1 to S10 [file hds.0198.f1.docx]

Supplementary Material

[Supplementary Table 1. ICD-10 used in this study 2](#_Toc177589554)

[Supplementary Table 2. Detailed characteristics of patients belonging to different mobility patterns 3](#_Toc177589555)

[Supplementary Table 3. Cancer patients and definitions of subgroups 5](#_Toc177589556)

[Supplementary Table 4. Matching results for overall patients 6](#_Toc177589557)

[Supplementary Table 5. Matching results for patients with the five most common cancer 11](#_Toc177589558)

[Supplementary Table 6. Matching results for patients with uncommon cancer 15](#_Toc177589559)

[Supplementary Table 7. Matching results for cancer patients in cities with healthcare resources more than average level 20](#_Toc177589560)

[Supplementary Table 8. Matching results for cancer patients in cities with healthcare resources less than average level 24](#_Toc177589561)

[Supplementary Table 9. Five-year survival for sequentially matched patients and hazard ratio of all-cause mortality risk for local and national center patterns vs. intra-city pattern in sensitivity analysis. 29](#_Toc177589562)

[Supplementary Table 10. Five-year survival for sequentially matched patients and hazard ratio of all-cause mortality risk for local and national center patterns vs. intra-city pattern by healthcare resource and cancer type. 30](#_Toc177589563)

# Supplementary Table 1. ICD-10 used in this study

| Sites | ICD-10 |
| --- | --- |
| Oral cavity & pharynx | C00-C10, C12-C14 |
| Nasopharynx | C11 |
| Esophagus | C15 |
| Stomach | C16 |
| Colorectum | C18-21 |
| Liver | C22 |
| Gallbladder | C23-24 |
| Pancreas | C25 |
| Larynx | C32 |
| Lung | C33-34 |
| Other thoracic organs | C37-38 |
| Melanoma of the skin | C43 |
| Other skin cancer | C44 |
| Breast | C50 |
| Cervix | C53 |
| Prostate | C61 |
| Kidney | C64-66, C68 |
| Bladder | C67 |
| Brain, CNS | C70-72 |
| Thyroid | C73 |
| Lymphoma | C81-85, C88, C90, C96 |
| Leukemia | C91-95 |

Common cancers: lung, colorectum, stomach, breast, thyroid. Uncommon cancers: other sites in this table.

# Supplementary Table 2. Detailed characteristics of patients belonging to different mobility patterns

| Variables | Overall | Intra-city | Local center | P-value^1^ | National center | P-value^2^ |
| --- | --- | --- | --- | --- | --- | --- |
| N | 20602 | 17035 | 2974 |  | 593 |  |
| Age group (%) |  |  |  | <0.001 |  | <0.001 |
| < 40 | 1285 (6.2) | 950 (5.6) | 258 (8.7) |  | 77 (13.0) |  |
| 40-50 | 2550 (12.4) | 1950 (11.4) | 461 (15.5) |  | 139 (23.4) |  |
| 50-60 | 4675 (22.7) | 3795 (22.3) | 732 (24.6) |  | 148 (25.0) |  |
| > 60 | 12092 (58.7) | 10340 (60.7) | 1523 (51.2) |  | 229 (38.6) |  |
| Sex (%) |  |  |  | <0.001 |  | 0.004 |
| Male | 10983 (53.3) | 8898 (52.2) | 1739 (58.5) |  | 346 (58.3) |  |
| Female | 9619 (46.7) | 8137 (47.8) | 1235 (41.5) |  | 247 (41.7) |  |
| First hospitalization year (%) |  |  |  | <0.001 |  | <0.001 |
| 2015 | 7769 (37.7) | 6350 (37.3) | 1201 (40.4) |  | 218 (36.8) |  |
| 2016 | 6926 (33.6) | 5626 (33.0) | 1062 (35.7) |  | 238 (40.1) |  |
| 2017 | 5907 (28.7) | 5059 (29.7) | 711 (23.9) |  | 137 (23.1) |  |
| City (%) |  |  |  | <0.001 |  | <0.001 |
| Binzhou | 717 (3.5) | 584 (3.4) | 121 (4.1) |  | 12 (2.0) |  |
| Dezhou | 1247 (6.1) | 756 (4.4) | 423 (14.2) |  | 68 (11.5) |  |
| Dongying | 1555 (7.5) | 1036 (6.1) | 389 (13.1) |  | 130 (21.9) |  |
| Heze | 718 (3.5) | 160 (0.9) | 518 (17.4) |  | 40 (6.7) |  |
| Jinan | 1695 (8.2) | 1678 (9.9) | 16 (0.5) |  | 1 (0.2) |  |
| Jining | 1728 (8.4) | 1313 (7.7) | 332 (11.2) |  | 83 (14.0) |  |
| Liaocheng | 257 (1.2) | 30 (0.2) | 227 (7.6) |  | 0 (0.0) |  |
| Linyi | 726 (3.5) | 590 (3.5) | 89 (3.0) |  | 47 (7.9) |  |
| Qingdao | 6264 (30.4) | 6232 (36.6) | 29 (1.0) |  | 3 (0.5) |  |
| Taian | 1063 (5.2) | 816 (4.8) | 223 (7.5) |  | 24 (4.0) |  |
| Weifang | 418 (2.0) | 388 (2.3) | 21 (0.7) |  | 9 (1.5) |  |
| Weihai | 1424 (6.9) | 1216 (7.1) | 114 (3.8) |  | 94 (15.9) |  |
| Yantai | 1296 (6.3) | 1144 (6.7) | 144 (4.8) |  | 8 (1.3) |  |
| Zaozhuang | 215 (1.0) | 96 (0.6) | 117 (3.9) |  | 2 (0.3) |  |
| Zibo | 1279 (6.2) | 996 (5.8) | 211 (7.1) |  | 72 (12.1) |  |
| Marital status (%) |  |  |  | <0.001 |  | <0.001 |
| Married | 12864 (62.4) | 10864 (63.8) | 1628 (54.7) |  | 372 (62.7) |  |
| Single | 306 (1.5) | 224 (1.3) | 58 (2.0) |  | 24 (4.0) |  |
| Widowed/divorced | 607 (2.9) | 549 (3.2) | 55 (1.8) |  | 3 (0.5) |  |
| Unknown | 6825 (33.1) | 5398 (31.7) | 1233 (41.5) |  | 194 (32.7) |  |
| Cancer type (%) |  |  |  | <0.001 |  | <0.001 |
| Bladder | 459 (2.2) | 410 (2.4) | 41 (1.4) |  | 8 (1.3) |  |
| Brain | 268 (1.3) | 225 (1.3) | 24 (0.8) |  | 19 (3.2) |  |
| Breast | 2324 (11.3) | 2212 (13.0) | 70 (2.4) |  | 42 (7.1) |  |
| Cervix | 693 (3.4) | 527 (3.1) | 155 (5.2) |  | 11 (1.9) |  |
| Colorectum | 3566 (17.3) | 2529 (14.8) | 975 (32.8) |  | 62 (10.5) |  |
| Gallbladder | 287 (1.4) | 244 (1.4) | 32 (1.1) |  | 11 (1.9) |  |
| Kidney | 494 (2.4) | 396 (2.3) | 83 (2.8) |  | 15 (2.5) |  |
| Larynx | 107 (0.5) | 92 (0.5) | 15 (0.5) |  | 0 (0.0) |  |
| Leukemia | 364 (1.8) | 282 (1.7) | 44 (1.5) |  | 38 (6.4) |  |
| Liver | 1196 (5.8) | 965 (5.7) | 171 (5.7) |  | 60 (10.1) |  |
| Lung | 3958 (19.2) | 3389 (19.9) | 477 (16.0) |  | 92 (15.5) |  |
| Lymphoma | 691 (3.4) | 545 (3.2) | 103 (3.5) |  | 43 (7.3) |  |
| Melanoma | 55 (0.3) | 41 (0.2) | 14 (0.5) |  | 0 (0.0) |  |
| Nasopharynx | 114 (0.6) | 83 (0.5) | 25 (0.8) |  | 6 (1.0) |  |
| Esophagus | 1320 (6.4) | 1126 (6.6) | 172 (5.8) |  | 22 (3.7) |  |
| Oral | 249 (1.2) | 200 (1.2) | 40 (1.3) |  | 9 (1.5) |  |
| Other skin | 100 (0.5) | 89 (0.5) | 11 (0.4) |  | 0 (0.0) |  |
| Other thoracic | 61 (0.3) | 46 (0.3) | 15 (0.5) |  | 0 (0.0) |  |
| Pancreas | 296 (1.4) | 262 (1.5) | 24 (0.8) |  | 10 (1.7) |  |
| Prostate | 361 (1.8) | 313 (1.8) | 31 (1.0) |  | 17 (2.9) |  |
| Stomach | 2161 (10.5) | 1882 (11.0) | 231 (7.8) |  | 48 (8.1) |  |
| Thyroid | 1478 (7.2) | 1177 (6.9) | 221 (7.4) |  | 80 (13.5) |  |
| Hypertension (%) |  |  |  | <0.001 |  | 0.009 |
| No | 19962 (96.9) | 16456 (96.6) | 2921 (98.2) |  | 585 (98.7) |  |
| Yes | 640 (3.1) | 579 (3.4) | 53 (1.8) |  | 8 (1.3) |  |
| Diabetes (%) |  |  |  | <0.001 |  | 0.782 |
| No | 20036 (97.3) | 16527 (97.0) | 2932 (98.6) |  | 577 (97.3) |  |
| Yes | 566 (2.7) | 508 (3.0) | 42 (1.4) |  | 16 (2.7) |  |
| Heart (%) |  |  |  | <0.001 |  | 0.015 |
| No | 19396 (94.1) | 15974 (93.8) | 2851 (95.9) |  | 571 (96.3) |  |
| Yes | 1206 (5.9) | 1061 (6.2) | 123 (4.1) |  | 22 (3.7) |  |
| Osteoporosis (%) |  |  |  | <0.001 |  | <0.001 |
| No | 17884 (86.8) | 15199 (89.2) | 2209 (74.3) |  | 476 (80.3) |  |
| Yes | 2718 (13.2) | 1836 (10.8) | 765 (25.7) |  | 117 (19.7) |  |
| Cerebral (%) |  |  |  | 0.488 |  | 0.006 |
| No | 19570 (95.0) | 16161 (94.9) | 2831 (95.2) |  | 578 (97.5) |  |
| Yes | 1032 (5.0) | 874 (5.1) | 143 (4.8) |  | 15 (2.5) |  |
| Comorbidity (%) |  |  |  | <0.001 |  | 0.336 |
| No | 15238 (74.0) | 12835 (75.3) | 1967 (66.1) |  | 436 (73.5) |  |
| Yes | 5364 (26.0) | 4200 (24.7) | 1007 (33.9) |  | 157 (26.5) |  |
| Hospitalization frequency | 3.5 | 3.4 | 4.0 | <0.001 | 3.8 | 0.021 |
| Out-of-pocket costs ($) | 1202.7 | 995.9 | 2183.0 | <0.001 | 2228.4 | <0.001 |

^1^The p values are calculated between the patients belonging to intra-city and local center patterns. ^1^The p values are calculated between the patients belonging to intra-city and national center patterns.

# Supplementary Table 3. Cancer patients and definitions of subgroups

|  | Five most common cancers | Uncommon cancers | P-value |
| --- | --- | --- | --- |
| Patterns (%) |  |  | <0.001 |
| Intra-city | 1974(14.6) | 1000(14.1) |  |
| Local center | 324(2.4) | 269(3.8) |  |
| National center | 11189(83.0) | 5846(82.2) |  |
| Sites | Lung, Colorectum, Stomach, Breast, Thyroid | Oral cavity & pharynx, Nasopharynx, Esophagus, Liver, Gallbladder, Pancreas, Larynx, Other thoracic organs, Melanoma of the skin, Other skin cancer, Cervix, Prostate, Kidney, Bladder, Brain, CNS, Lymphoma, Leukemia |  |
|  | Cities with above-average healthcare resources | Cities with below-average healthcare resources |  |
| Patterns (%) |  |  | <0.001 |
| Intra-city | 13118(90.0) | 3917(65.0) |  |
| Local center | 1126(7.7) | 1848(30.7) |  |
| National center | 332(2.2) | 261(4.3) |  |
| Cities | Dezhou, Heze, Linyi, Liaocheng, Zaozhuang, Weifang, Binzhou, Jining | Taian, Yantai, Qingdao, Weihai, Zibo, Dongying, Jinan |  |

# Supplementary Table 4. Matching results for overall patients

|  | Demographic matched | | | Marriage matched | | | Cancer matched | | | Morbidity matched | | | Frequency matched | | |
| --- | --- | --- | --- | --- | --- | --- | --- | --- | --- | --- | --- | --- | --- | --- | --- |
| Variables | Local | Intra-city | SMD | Local | Intra-city | SMD | Local | Intra-city | SMD | Local | Intra-city | SMD | Local | Intra-city | SMD |
| Age group |  |  |  |  |  |  |  |  |  |  |  |  |  |  |  |
| Less40 | 0.09 | 0.08 | 0.03 | 0.10 | 0.08 | 0.06 | 0.09 | 0.07 | 0.05 | 0.09 | 0.07 | 0.06 | 0.08 | 0.07 | 0.03 |
| 40-50 | 0.14 | 0.14 | -0.01 | 0.13 | 0.14 | -0.05 | 0.14 | 0.13 | 0.03 | 0.15 | 0.13 | 0.03 | 0.15 | 0.14 | 0.02 |
| 50-60 | 0.24 | 0.26 | -0.02 | 0.25 | 0.26 | -0.01 | 0.24 | 0.25 | -0.01 | 0.24 | 0.25 | -0.02 | 0.25 | 0.25 | 0.00 |
| Greater60 | 0.53 | 0.52 | 0.01 | 0.52 | 0.52 | 0.01 | 0.53 | 0.55 | -0.04 | 0.53 | 0.55 | -0.04 | 0.53 | 0.54 | -0.03 |
| Sex |  |  |  |  |  |  |  |  |  |  |  |  |  |  |  |
| Male | 0.57 | 0.57 | 0.00 | 0.57 | 0.57 | 0.00 | 0.58 | 0.58 | 0.00 | 0.58 | 0.58 | 0.00 | 0.58 | 0.58 | 0.00 |
| Female | 0.43 | 0.43 | 0.00 | 0.43 | 0.43 | 0.00 | 0.42 | 0.42 | 0.00 | 0.42 | 0.42 | 0.00 | 0.42 | 0.42 | 0.00 |
| First hospitalization year |  |  |  |  |  |  |  |  |  |  |  |  |  |  |  |
| 2015 | 0.45 | 0.41 | 0.08 | 0.37 | 0.39 | -0.04 | 0.37 | 0.39 | -0.05 | 0.40 | 0.39 | 0.02 | 0.39 | 0.39 | 0.01 |
| 2016 | 0.31 | 0.35 | -0.08 | 0.37 | 0.35 | 0.04 | 0.37 | 0.35 | 0.04 | 0.35 | 0.35 | 0.00 | 0.34 | 0.34 | 0.00 |
| 2017 | 0.24 | 0.24 | -0.01 | 0.25 | 0.25 | -0.01 | 0.26 | 0.26 | 0.01 | 0.25 | 0.26 | -0.02 | 0.26 | 0.27 | -0.01 |
| City |  |  |  |  |  |  |  |  |  |  |  |  |  |  |  |
| Binzhou | 0.05 | 0.05 | 0.00 | 0.05 | 0.05 | 0.00 | 0.05 | 0.05 | 0.00 | 0.05 | 0.05 | 0.00 | 0.05 | 0.05 | 0.00 |
| Dezhou | 0.17 | 0.17 | 0.00 | 0.15 | 0.15 | 0.00 | 0.15 | 0.15 | 0.00 | 0.15 | 0.15 | 0.00 | 0.15 | 0.15 | 0.00 |
| Dongying | 0.17 | 0.17 | 0.00 | 0.16 | 0.16 | 0.00 | 0.17 | 0.17 | 0.00 | 0.17 | 0.17 | 0.00 | 0.17 | 0.17 | 0.00 |
| Heze | 0.07 | 0.07 | 0.00 | 0.07 | 0.07 | 0.00 | 0.07 | 0.07 | 0.00 | 0.07 | 0.07 | 0.00 | 0.07 | 0.07 | 0.00 |
| Jinan | 0.01 | 0.01 | 0.00 | 0.01 | 0.01 | 0.00 | 0.01 | 0.01 | 0.00 | 0.01 | 0.01 | 0.00 | 0.01 | 0.01 | 0.00 |
| Jining | 0.14 | 0.14 | 0.00 | 0.15 | 0.15 | 0.00 | 0.14 | 0.14 | 0.00 | 0.14 | 0.14 | 0.00 | 0.14 | 0.14 | 0.00 |
| Liaocheng | 0.01 | 0.01 | 0.00 | 0.01 | 0.01 | 0.00 | 0.01 | 0.01 | 0.00 | 0.01 | 0.01 | 0.00 | 0.01 | 0.01 | 0.00 |
| Linyi | 0.04 | 0.04 | 0.00 | 0.04 | 0.04 | 0.00 | 0.04 | 0.04 | 0.00 | 0.04 | 0.04 | 0.00 | 0.04 | 0.04 | 0.00 |
| Qingdao | 0.01 | 0.01 | 0.00 | 0.01 | 0.01 | 0.00 | 0.01 | 0.01 | 0.00 | 0.01 | 0.01 | 0.00 | 0.01 | 0.01 | 0.00 |
| Taian | 0.10 | 0.10 | 0.00 | 0.10 | 0.10 | 0.00 | 0.10 | 0.10 | 0.00 | 0.10 | 0.10 | 0.00 | 0.09 | 0.09 | 0.00 |
| Weifang | 0.01 | 0.01 | 0.00 | 0.01 | 0.01 | 0.00 | 0.01 | 0.01 | 0.00 | 0.01 | 0.01 | 0.00 | 0.01 | 0.01 | 0.00 |
| Weihai | 0.05 | 0.05 | 0.00 | 0.05 | 0.05 | 0.00 | 0.05 | 0.05 | 0.00 | 0.05 | 0.05 | 0.00 | 0.05 | 0.05 | 0.00 |
| Yantai | 0.06 | 0.06 | 0.00 | 0.06 | 0.06 | 0.00 | 0.06 | 0.06 | 0.00 | 0.06 | 0.06 | 0.00 | 0.06 | 0.06 | 0.00 |
| Zaozhuang | 0.03 | 0.03 | 0.00 | 0.03 | 0.03 | 0.00 | 0.03 | 0.03 | 0.00 | 0.03 | 0.03 | 0.00 | 0.03 | 0.03 | 0.00 |
| Zibo | 0.09 | 0.09 | 0.00 | 0.09 | 0.09 | 0.00 | 0.09 | 0.09 | 0.00 | 0.09 | 0.09 | 0.00 | 0.09 | 0.09 | 0.00 |
| Marital status |  |  |  |  |  |  |  |  |  |  |  |  |  |  |  |
| Married | 0.55 | 0.67 | -0.24 | 0.58 | 0.58 | 0.00 | 0.61 | 0.61 | -0.01 | 0.61 | 0.61 | -0.02 | 0.61 | 0.62 | -0.01 |
| Single | 0.02 | 0.01 | 0.04 | 0.02 | 0.02 | -0.01 | 0.02 | 0.02 | 0.02 | 0.02 | 0.02 | 0.01 | 0.02 | 0.02 | -0.02 |
| Widowed/divorced | 0.02 | 0.05 | -0.19 | 0.02 | 0.02 | 0.01 | 0.02 | 0.02 | 0.00 | 0.02 | 0.02 | 0.00 | 0.02 | 0.02 | 0.01 |
| Unknown | 0.41 | 0.27 | 0.29 | 0.38 | 0.38 | 0.00 | 0.35 | 0.35 | 0.01 | 0.35 | 0.34 | 0.02 | 0.34 | 0.34 | 0.02 |
| Cancer type |  |  |  |  |  |  |  |  |  |  |  |  |  |  |  |
| Bladder | 0.01 | 0.03 | -0.15 | 0.01 | 0.03 | -0.11 | 0.02 | 0.02 | 0.00 | 0.02 | 0.02 | 0.01 | 0.02 | 0.02 | 0.01 |
| Brain | 0.01 | 0.02 | -0.06 | 0.01 | 0.01 | -0.06 | 0.01 | 0.01 | 0.03 | 0.01 | 0.01 | 0.02 | 0.01 | 0.01 | -0.01 |
| Breast | 0.03 | 0.11 | -0.55 | 0.02 | 0.11 | -0.57 | 0.02 | 0.03 | -0.01 | 0.02 | 0.03 | -0.02 | 0.02 | 0.03 | -0.02 |
| Cervix | 0.05 | 0.04 | 0.04 | 0.06 | 0.04 | 0.09 | 0.06 | 0.06 | 0.01 | 0.06 | 0.06 | 0.01 | 0.06 | 0.05 | 0.02 |
| Colorectum | 0.33 | 0.15 | 0.39 | 0.30 | 0.17 | 0.29 | 0.25 | 0.26 | -0.01 | 0.26 | 0.27 | -0.01 | 0.25 | 0.26 | -0.02 |
| Gallbladder | 0.01 | 0.01 | -0.01 | 0.01 | 0.01 | -0.02 | 0.01 | 0.01 | -0.02 | 0.01 | 0.01 | -0.03 | 0.01 | 0.01 | -0.01 |
| Kidney | 0.03 | 0.03 | 0.01 | 0.03 | 0.03 | 0.02 | 0.03 | 0.03 | 0.01 | 0.03 | 0.03 | 0.02 | 0.03 | 0.03 | 0.02 |
| Larynx | 0.00 | 0.01 | -0.06 | 0.00 | 0.01 | -0.03 | 0.01 | 0.01 | -0.01 | 0.01 | 0.00 | 0.03 | 0.00 | 0.00 | 0.00 |
| Leukemia | 0.02 | 0.02 | -0.07 | 0.02 | 0.03 | -0.10 | 0.02 | 0.02 | 0.00 | 0.02 | 0.02 | 0.00 | 0.02 | 0.02 | 0.00 |
| Liver | 0.06 | 0.07 | -0.04 | 0.06 | 0.07 | -0.03 | 0.06 | 0.07 | -0.04 | 0.06 | 0.07 | -0.01 | 0.06 | 0.06 | 0.00 |
| Lung | 0.16 | 0.17 | -0.03 | 0.17 | 0.16 | 0.02 | 0.18 | 0.18 | 0.00 | 0.18 | 0.18 | -0.01 | 0.18 | 0.18 | 0.00 |
| Lymphoma | 0.04 | 0.04 | -0.03 | 0.04 | 0.04 | 0.00 | 0.04 | 0.04 | 0.01 | 0.04 | 0.04 | -0.01 | 0.04 | 0.04 | 0.02 |
| Melanoma | 0.01 | 0.00 | 0.05 | 0.01 | 0.00 | 0.05 | 0.00 | 0.00 | -0.01 | 0.00 | 0.01 | -0.03 | 0.00 | 0.00 | -0.01 |
| Nasopharynx | 0.01 | 0.00 | 0.05 | 0.01 | 0.00 | 0.05 | 0.01 | 0.01 | 0.04 | 0.01 | 0.01 | 0.05 | 0.01 | 0.01 | 0.03 |
| Esophagus | 0.05 | 0.06 | -0.05 | 0.06 | 0.06 | -0.03 | 0.07 | 0.06 | 0.02 | 0.06 | 0.06 | -0.03 | 0.06 | 0.07 | -0.02 |
| Oral | 0.01 | 0.01 | 0.01 | 0.01 | 0.01 | 0.02 | 0.02 | 0.01 | 0.03 | 0.02 | 0.02 | 0.00 | 0.02 | 0.02 | -0.01 |
| Other skin | 0.00 | 0.01 | -0.07 | 0.00 | 0.01 | -0.07 | 0.00 | 0.00 | -0.01 | 0.00 | 0.00 | 0.00 | 0.00 | 0.00 | 0.03 |
| Other thoracic | 0.00 | 0.00 | 0.04 | 0.00 | 0.00 | 0.04 | 0.00 | 0.00 | -0.01 | 0.00 | 0.00 | 0.03 | 0.01 | 0.00 | 0.04 |
| Pancreas | 0.01 | 0.02 | -0.09 | 0.01 | 0.01 | -0.07 | 0.01 | 0.01 | -0.03 | 0.01 | 0.01 | 0.00 | 0.01 | 0.01 | 0.01 |
| Prostate | 0.01 | 0.02 | -0.09 | 0.01 | 0.01 | -0.02 | 0.01 | 0.01 | -0.02 | 0.01 | 0.01 | 0.01 | 0.01 | 0.01 | -0.01 |
| Stomach | 0.08 | 0.10 | -0.05 | 0.09 | 0.09 | -0.03 | 0.09 | 0.09 | -0.01 | 0.09 | 0.09 | 0.00 | 0.09 | 0.10 | -0.02 |
| Thyroid | 0.07 | 0.08 | -0.03 | 0.07 | 0.08 | -0.05 | 0.08 | 0.07 | 0.02 | 0.08 | 0.07 | 0.04 | 0.08 | 0.07 | 0.01 |
| Hypertension |  |  |  |  |  |  |  |  |  |  |  |  |  |  |  |
| No | 0.98 | 0.97 | 0.07 | 0.98 | 0.97 | 0.06 | 0.98 | 0.98 | 0.04 | 0.98 | 0.98 | -0.02 | 0.98 | 0.98 | 0.00 |
| Yes | 0.02 | 0.03 | -0.07 | 0.02 | 0.03 | -0.06 | 0.02 | 0.02 | -0.04 | 0.02 | 0.02 | 0.02 | 0.02 | 0.02 | 0.00 |
| Diabetes |  |  |  |  |  |  |  |  |  |  |  |  |  |  |  |
| No | 0.99 | 0.98 | 0.11 | 0.99 | 0.98 | 0.08 | 0.99 | 0.98 | 0.08 | 0.99 | 0.99 | 0.00 | 0.99 | 0.99 | -0.01 |
| Yes | 0.01 | 0.02 | -0.11 | 0.01 | 0.02 | -0.08 | 0.01 | 0.02 | -0.08 | 0.01 | 0.01 | 0.00 | 0.01 | 0.01 | 0.01 |
| Heart |  |  |  |  |  |  |  |  |  |  |  |  |  |  |  |
| No | 0.96 | 0.93 | 0.14 | 0.96 | 0.94 | 0.11 | 0.96 | 0.94 | 0.09 | 0.95 | 0.96 | -0.04 | 0.95 | 0.96 | -0.02 |
| Yes | 0.04 | 0.07 | -0.14 | 0.04 | 0.06 | -0.11 | 0.04 | 0.06 | -0.09 | 0.05 | 0.04 | 0.04 | 0.05 | 0.04 | 0.02 |
| Osteoporosis |  |  |  |  |  |  |  |  |  |  |  |  |  |  |  |
| No | 0.79 | 0.78 | 0.04 | 0.79 | 0.77 | 0.06 | 0.80 | 0.77 | 0.07 | 0.81 | 0.81 | 0.01 | 0.80 | 0.79 | 0.02 |
| Yes | 0.21 | 0.22 | -0.04 | 0.21 | 0.23 | -0.06 | 0.20 | 0.23 | -0.07 | 0.19 | 0.19 | -0.01 | 0.20 | 0.21 | -0.02 |
| Cerebral |  |  |  |  |  |  |  |  |  |  |  |  |  |  |  |
| No | 0.95 | 0.94 | 0.05 | 0.95 | 0.95 | 0.01 | 0.95 | 0.94 | 0.06 | 0.95 | 0.96 | -0.05 | 0.95 | 0.96 | -0.02 |
| Yes | 0.05 | 0.06 | -0.05 | 0.05 | 0.05 | -0.01 | 0.05 | 0.06 | -0.06 | 0.05 | 0.04 | 0.05 | 0.05 | 0.04 | 0.02 |
| Comorbidity |  |  |  |  |  |  |  |  |  |  |  |  |  |  |  |
| No | 0.71 | 0.66 | 0.11 | 0.71 | 0.67 | 0.10 | 0.72 | 0.66 | 0.12 | 0.72 | 0.74 | -0.04 | 0.71 | 0.71 | 0.00 |
| Yes | 0.29 | 0.34 | -0.11 | 0.29 | 0.33 | -0.10 | 0.28 | 0.34 | -0.12 | 0.28 | 0.26 | 0.04 | 0.29 | 0.29 | 0.00 |
| Hospitalization frequency | 4.18 | 3.06 | 0.29 | 4.14 | 3.01 | 0.29 | 4.11 | 2.96 | 0.30 | 4.16 | 3.03 | 0.29 | 3.66 | 3.64 | 0.01 |
| Variables | National | Intra-city | SMD | National | Intra-city | SMD | National | Intra-city | SMD | National | Intra-city | SMD | National | Intra-city | SMD |
| Age group |  |  |  |  |  |  |  |  |  |  |  |  |  |  |  |
| Less40 | 0.12 | 0.12 | 0.01 | 0.11 | 0.10 | 0.03 | 0.10 | 0.10 | 0.00 | 0.11 | 0.11 | 0.02 | 0.11 | 0.11 | 0.02 |
| 40-50 | 0.24 | 0.24 | 0.00 | 0.24 | 0.24 | 0.00 | 0.23 | 0.20 | 0.07 | 0.22 | 0.21 | 0.03 | 0.21 | 0.20 | 0.04 |
| 50-60 | 0.25 | 0.25 | 0.00 | 0.25 | 0.25 | 0.00 | 0.26 | 0.25 | 0.02 | 0.25 | 0.22 | 0.07 | 0.26 | 0.25 | 0.01 |
| Greater60 | 0.39 | 0.40 | -0.01 | 0.40 | 0.40 | -0.02 | 0.41 | 0.45 | -0.07 | 0.41 | 0.46 | -0.10 | 0.42 | 0.45 | -0.06 |
| Sex |  |  |  |  |  |  |  |  |  |  |  |  |  |  |  |
| Male | 0.59 | 0.59 | 0.00 | 0.59 | 0.58 | 0.00 | 0.58 | 0.58 | 0.00 | 0.58 | 0.57 | 0.01 | 0.58 | 0.57 | 0.01 |
| Female | 0.41 | 0.41 | 0.00 | 0.41 | 0.42 | 0.00 | 0.42 | 0.42 | 0.00 | 0.42 | 0.43 | -0.01 | 0.42 | 0.43 | -0.01 |
| First hospitalization year |  |  |  |  |  |  |  |  |  |  |  |  |  |  |  |
| 2015 | 0.37 | 0.37 | 0.00 | 0.37 | 0.36 | 0.01 | 0.37 | 0.36 | 0.02 | 0.37 | 0.36 | 0.01 | 0.37 | 0.36 | 0.03 |
| 2016 | 0.40 | 0.39 | 0.01 | 0.40 | 0.40 | -0.01 | 0.39 | 0.43 | -0.10 | 0.39 | 0.39 | -0.01 | 0.38 | 0.40 | -0.04 |
| 2017 | 0.23 | 0.23 | 0.00 | 0.23 | 0.24 | -0.01 | 0.25 | 0.21 | 0.08 | 0.25 | 0.25 | 0.00 | 0.25 | 0.24 | 0.02 |
| City |  |  |  |  |  |  |  |  |  |  |  |  |  |  |  |
| Binzhou | 0.02 | 0.02 | 0.00 | 0.02 | 0.02 | 0.00 | 0.02 | 0.02 | 0.00 | 0.02 | 0.02 | 0.00 | 0.02 | 0.02 | -0.01 |
| Dezhou | 0.12 | 0.12 | 0.00 | 0.12 | 0.12 | -0.01 | 0.12 | 0.10 | 0.05 | 0.11 | 0.11 | 0.02 | 0.11 | 0.10 | 0.03 |
| Dongying | 0.22 | 0.22 | 0.01 | 0.22 | 0.21 | 0.02 | 0.21 | 0.21 | 0.01 | 0.21 | 0.21 | 0.01 | 0.21 | 0.21 | 0.01 |
| Heze | 0.06 | 0.05 | 0.02 | 0.05 | 0.05 | 0.01 | 0.05 | 0.04 | 0.02 | 0.05 | 0.04 | 0.02 | 0.04 | 0.04 | 0.02 |
| Jinan | 0.00 | 0.00 | 0.00 | 0.00 | 0.00 | 0.00 | 0.00 | 0.00 | 0.00 | 0.00 | 0.00 | 0.00 | 0.00 | 0.00 | 0.00 |
| Jining | 0.14 | 0.14 | -0.01 | 0.14 | 0.15 | -0.01 | 0.15 | 0.15 | -0.01 | 0.15 | 0.15 | -0.01 | 0.15 | 0.15 | 0.00 |
| Linyi | 0.08 | 0.08 | 0.00 | 0.08 | 0.08 | 0.00 | 0.08 | 0.08 | -0.01 | 0.08 | 0.08 | 0.00 | 0.08 | 0.08 | -0.01 |
| Qingdao | 0.01 | 0.01 | 0.00 | 0.01 | 0.01 | 0.00 | 0.01 | 0.01 | 0.00 | 0.01 | 0.01 | 0.00 | 0.01 | 0.01 | 0.00 |
| Taian | 0.04 | 0.04 | 0.00 | 0.04 | 0.04 | 0.00 | 0.04 | 0.04 | -0.01 | 0.04 | 0.04 | -0.01 | 0.04 | 0.05 | -0.01 |
| Weifang | 0.02 | 0.02 | 0.00 | 0.02 | 0.02 | 0.00 | 0.01 | 0.01 | 0.00 | 0.01 | 0.01 | 0.01 | 0.01 | 0.01 | 0.00 |
| Weihai | 0.16 | 0.16 | -0.01 | 0.16 | 0.17 | -0.01 | 0.17 | 0.17 | -0.02 | 0.17 | 0.17 | -0.02 | 0.17 | 0.18 | -0.02 |
| Yantai | 0.01 | 0.01 | 0.00 | 0.01 | 0.01 | 0.00 | 0.01 | 0.02 | -0.01 | 0.01 | 0.02 | -0.01 | 0.01 | 0.02 | -0.01 |
| Zaozhuang | 0.00 | 0.00 | 0.00 | 0.00 | 0.00 | 0.00 | 0.00 | 0.00 | 0.00 | 0.00 | 0.00 | 0.00 | 0.00 | 0.00 | 0.00 |
| Zibo | 0.12 | 0.12 | 0.00 | 0.12 | 0.12 | -0.01 | 0.12 | 0.13 | -0.02 | 0.13 | 0.13 | -0.01 | 0.13 | 0.13 | -0.01 |
| Marital status |  |  |  |  |  |  |  |  |  |  |  |  |  |  |  |
| Married | 0.63 | 0.65 | -0.04 | 0.63 | 0.65 | -0.04 | 0.63 | 0.64 | -0.01 | 0.64 | 0.65 | -0.04 | 0.63 | 0.69 | -0.11 |
| Single | 0.04 | 0.02 | 0.09 | 0.03 | 0.02 | 0.06 | 0.03 | 0.03 | 0.00 | 0.03 | 0.02 | 0.05 | 0.03 | 0.02 | 0.06 |
| Widowed/divorced | 0.01 | 0.03 | -0.28 | 0.01 | 0.00 | 0.01 | 0.01 | 0.00 | 0.02 | 0.01 | 0.00 | 0.01 | 0.01 | 0.01 | 0.00 |
| Unknown | 0.33 | 0.30 | 0.05 | 0.33 | 0.32 | 0.01 | 0.34 | 0.33 | 0.01 | 0.33 | 0.32 | 0.02 | 0.33 | 0.29 | 0.09 |
| Cancer type |  |  |  |  |  |  |  |  |  |  |  |  |  |  |  |
| Bladder | 0.01 | 0.03 | -0.13 | 0.01 | 0.03 | -0.12 | 0.01 | 0.01 | 0.02 | 0.01 | 0.01 | 0.04 | 0.01 | 0.02 | -0.01 |
| Brain | 0.03 | 0.02 | 0.05 | 0.03 | 0.03 | 0.01 | 0.03 | 0.02 | 0.05 | 0.03 | 0.02 | 0.06 | 0.04 | 0.02 | 0.06 |
| Breast | 0.07 | 0.14 | -0.25 | 0.07 | 0.13 | -0.22 | 0.08 | 0.09 | -0.06 | 0.08 | 0.08 | -0.03 | 0.08 | 0.09 | -0.03 |
| Cervix | 0.02 | 0.04 | -0.12 | 0.02 | 0.03 | -0.11 | 0.02 | 0.02 | -0.02 | 0.02 | 0.02 | 0.02 | 0.02 | 0.03 | -0.05 |
| Colorectum | 0.11 | 0.14 | -0.10 | 0.11 | 0.14 | -0.10 | 0.11 | 0.13 | -0.05 | 0.11 | 0.12 | -0.03 | 0.11 | 0.10 | 0.04 |
| Gallbladder | 0.02 | 0.01 | 0.06 | 0.02 | 0.01 | 0.06 | 0.02 | 0.02 | 0.03 | 0.02 | 0.02 | 0.01 | 0.02 | 0.02 | 0.01 |
| Kidney | 0.03 | 0.03 | -0.03 | 0.02 | 0.03 | -0.03 | 0.03 | 0.02 | 0.05 | 0.03 | 0.02 | 0.03 | 0.03 | 0.03 | 0.00 |
| Leukemia | 0.06 | 0.04 | 0.11 | 0.06 | 0.03 | 0.12 | 0.04 | 0.04 | -0.01 | 0.04 | 0.04 | -0.03 | 0.04 | 0.04 | 0.00 |
| Liver | 0.10 | 0.06 | 0.13 | 0.10 | 0.06 | 0.15 | 0.10 | 0.10 | 0.00 | 0.10 | 0.11 | -0.01 | 0.09 | 0.10 | -0.04 |
| Lung | 0.16 | 0.16 | -0.01 | 0.16 | 0.16 | 0.00 | 0.17 | 0.18 | -0.03 | 0.17 | 0.18 | -0.04 | 0.17 | 0.17 | 0.00 |
| Lymphoma | 0.07 | 0.05 | 0.09 | 0.07 | 0.04 | 0.09 | 0.06 | 0.04 | 0.06 | 0.06 | 0.05 | 0.01 | 0.05 | 0.06 | -0.01 |
| Nasopharynx | 0.01 | 0.01 | 0.05 | 0.01 | 0.00 | 0.07 | 0.01 | 0.00 | 0.06 | 0.01 | 0.01 | 0.02 | 0.01 | 0.01 | 0.00 |
| Esophagus | 0.04 | 0.05 | -0.04 | 0.04 | 0.05 | -0.06 | 0.04 | 0.04 | 0.01 | 0.04 | 0.03 | 0.03 | 0.04 | 0.03 | 0.03 |
| Oral | 0.02 | 0.02 | -0.03 | 0.02 | 0.02 | -0.02 | 0.02 | 0.02 | 0.01 | 0.02 | 0.01 | 0.02 | 0.02 | 0.02 | 0.00 |
| Pancreas | 0.02 | 0.02 | 0.01 | 0.02 | 0.02 | 0.01 | 0.01 | 0.01 | 0.02 | 0.02 | 0.01 | 0.02 | 0.02 | 0.01 | 0.05 |
| Prostate | 0.03 | 0.02 | 0.08 | 0.03 | 0.01 | 0.09 | 0.03 | 0.03 | 0.01 | 0.03 | 0.03 | 0.01 | 0.03 | 0.03 | 0.03 |
| Stomach | 0.08 | 0.10 | -0.07 | 0.08 | 0.10 | -0.07 | 0.09 | 0.09 | -0.02 | 0.08 | 0.10 | -0.04 | 0.09 | 0.10 | -0.05 |
| Thyroid | 0.13 | 0.10 | 0.11 | 0.14 | 0.11 | 0.08 | 0.14 | 0.14 | 0.01 | 0.14 | 0.13 | 0.02 | 0.14 | 0.14 | -0.01 |
| Hypertension |  |  |  |  |  |  |  |  |  |  |  |  |  |  |  |
| No | 0.99 | 0.97 | 0.14 | 0.99 | 0.97 | 0.12 | 0.99 | 0.98 | 0.08 | 0.99 | 0.98 | 0.01 | 0.99 | 0.99 | -0.06 |
| Yes | 0.01 | 0.03 | -0.14 | 0.01 | 0.03 | -0.12 | 0.01 | 0.02 | -0.08 | 0.01 | 0.02 | -0.01 | 0.01 | 0.01 | 0.06 |
| Diabetes |  |  |  |  |  |  |  |  |  |  |  |  |  |  |  |
| No | 0.97 | 0.98 | -0.04 | 0.97 | 0.98 | -0.04 | 0.97 | 0.98 | -0.03 | 0.98 | 0.98 | 0.01 | 0.98 | 0.98 | 0.00 |
| Yes | 0.03 | 0.02 | 0.04 | 0.03 | 0.02 | 0.04 | 0.03 | 0.02 | 0.03 | 0.02 | 0.02 | -0.01 | 0.02 | 0.02 | 0.00 |
| Heart |  |  |  |  |  |  |  |  |  |  |  |  |  |  |  |
| No | 0.96 | 0.94 | 0.13 | 0.96 | 0.94 | 0.10 | 0.96 | 0.95 | 0.07 | 0.96 | 0.97 | -0.03 | 0.96 | 0.97 | -0.03 |
| Yes | 0.04 | 0.06 | -0.13 | 0.04 | 0.06 | -0.10 | 0.04 | 0.05 | -0.07 | 0.04 | 0.03 | 0.03 | 0.04 | 0.03 | 0.03 |
| Osteoporosis |  |  |  |  |  |  |  |  |  |  |  |  |  |  |  |
| No | 0.80 | 0.82 | -0.03 | 0.80 | 0.80 | 0.01 | 0.80 | 0.81 | -0.03 | 0.81 | 0.81 | 0.00 | 0.81 | 0.82 | -0.01 |
| Yes | 0.20 | 0.18 | 0.03 | 0.20 | 0.20 | -0.01 | 0.20 | 0.19 | 0.03 | 0.19 | 0.19 | 0.00 | 0.19 | 0.18 | 0.01 |
| Cerebral |  |  |  |  |  |  |  |  |  |  |  |  |  |  |  |
| No | 0.97 | 0.95 | 0.18 | 0.98 | 0.95 | 0.18 | 0.97 | 0.95 | 0.17 | 0.97 | 0.98 | -0.04 | 0.97 | 0.98 | -0.06 |
| Yes | 0.03 | 0.05 | -0.18 | 0.02 | 0.05 | -0.18 | 0.03 | 0.05 | -0.17 | 0.03 | 0.02 | 0.04 | 0.03 | 0.02 | 0.06 |
| Comorbidity |  |  |  |  |  |  |  |  |  |  |  |  |  |  |  |
| No | 0.74 | 0.70 | 0.07 | 0.74 | 0.69 | 0.10 | 0.73 | 0.71 | 0.05 | 0.74 | 0.75 | -0.02 | 0.74 | 0.76 | -0.04 |
| Yes | 0.26 | 0.30 | -0.07 | 0.26 | 0.31 | -0.10 | 0.27 | 0.29 | -0.05 | 0.26 | 0.25 | 0.02 | 0.26 | 0.24 | 0.04 |
| Hospitalization frequency | 3.78 | 3.16 | 0.17 | 3.79 | 3.20 | 0.16 | 3.73 | 3.17 | 0.15 | 3.74 | 3.33 | 0.11 | 3.53 | 3.34 | 0.05 |

Local, local center pattern; National, National center pattern; SMD, Standardized mean difference.

# Supplementary Table 5. Matching results for patients with the five most common cancer

|  | Demographic matched | | | Marriage matched | | | Cancer matched | | | Morbidity matched | | | Frequency matched | | |
| --- | --- | --- | --- | --- | --- | --- | --- | --- | --- | --- | --- | --- | --- | --- | --- |
| Variables | Local | Intra-city | SMD | Local | Intra-city | SMD | Local | Intra-city | SMD | Local | Intra-city | SMD | Local | Intra-city | SMD |
| Age group |  |  |  |  |  |  |  |  |  |  |  |  |  |  |  |
| Less40 | 0.17 | 0.14 | 0.09 | 0.14 | 0.14 | -0.01 | 0.14 | 0.12 | 0.05 | 0.16 | 0.12 | 0.10 | 0.15 | 0.12 | 0.06 |
| 40-50 | 0.23 | 0.25 | -0.04 | 0.27 | 0.25 | 0.04 | 0.24 | 0.25 | -0.01 | 0.22 | 0.24 | -0.03 | 0.23 | 0.24 | 0.00 |
| 50-60 | 0.52 | 0.54 | -0.05 | 0.51 | 0.54 | -0.07 | 0.54 | 0.58 | -0.07 | 0.54 | 0.58 | -0.07 | 0.56 | 0.58 | -0.05 |
| Greater60 | 0.08 | 0.07 | 0.02 | 0.09 | 0.07 | 0.07 | 0.08 | 0.06 | 0.09 | 0.08 | 0.07 | 0.04 | 0.06 | 0.06 | 0.03 |
| Sex |  |  |  |  |  |  |  |  |  |  |  |  |  |  |  |
| Male | 0.56 | 0.56 | 0.00 | 0.56 | 0.56 | 0.00 | 0.58 | 0.58 | 0.00 | 0.58 | 0.58 | 0.00 | 0.58 | 0.58 | 0.00 |
| Female | 0.44 | 0.44 | 0.00 | 0.44 | 0.44 | 0.00 | 0.42 | 0.42 | 0.00 | 0.42 | 0.42 | 0.00 | 0.42 | 0.42 | 0.00 |
| First hospitalization year |  |  |  |  |  |  |  |  |  |  |  |  |  |  |  |
| 2015 | 0.45 | 0.46 | -0.01 | 0.46 | 0.44 | 0.04 | 0.36 | 0.43 | -0.15 | 0.40 | 0.43 | -0.06 | 0.43 | 0.44 | -0.03 |
| 2016 | 0.35 | 0.34 | 0.01 | 0.32 | 0.34 | -0.04 | 0.40 | 0.31 | 0.18 | 0.37 | 0.33 | 0.08 | 0.34 | 0.31 | 0.06 |
| 2017 | 0.20 | 0.20 | 0.00 | 0.22 | 0.22 | -0.01 | 0.24 | 0.25 | -0.04 | 0.23 | 0.24 | -0.02 | 0.24 | 0.25 | -0.04 |
| City |  |  |  |  |  |  |  |  |  |  |  |  |  |  |  |
| Binzhou | 0.05 | 0.05 | 0.00 | 0.05 | 0.05 | 0.00 | 0.05 | 0.05 | 0.00 | 0.05 | 0.05 | 0.00 | 0.04 | 0.04 | 0.00 |
| Dezhou | 0.18 | 0.18 | 0.00 | 0.16 | 0.16 | 0.00 | 0.16 | 0.16 | 0.00 | 0.16 | 0.16 | 0.00 | 0.17 | 0.17 | 0.00 |
| Dongying | 0.15 | 0.15 | 0.00 | 0.16 | 0.16 | 0.00 | 0.17 | 0.17 | 0.00 | 0.17 | 0.17 | 0.00 | 0.17 | 0.17 | 0.00 |
| Heze | 0.04 | 0.04 | 0.00 | 0.04 | 0.04 | 0.00 | 0.05 | 0.05 | 0.00 | 0.05 | 0.05 | 0.00 | 0.05 | 0.05 | 0.00 |
| Jinan | 0.01 | 0.01 | 0.00 | 0.01 | 0.01 | 0.00 | 0.01 | 0.01 | 0.00 | 0.01 | 0.01 | 0.00 | 0.01 | 0.01 | 0.00 |
| Jining | 0.16 | 0.16 | 0.00 | 0.17 | 0.17 | 0.00 | 0.16 | 0.16 | 0.00 | 0.15 | 0.15 | 0.00 | 0.16 | 0.16 | 0.00 |
| Linyi | 0.04 | 0.04 | 0.00 | 0.04 | 0.04 | 0.00 | 0.04 | 0.04 | 0.00 | 0.04 | 0.04 | 0.00 | 0.04 | 0.04 | 0.00 |
| Qingdao | 0.01 | 0.01 | 0.00 | 0.01 | 0.01 | 0.00 | 0.02 | 0.02 | 0.00 | 0.02 | 0.02 | 0.00 | 0.02 | 0.02 | 0.00 |
| Taian | 0.11 | 0.11 | 0.00 | 0.11 | 0.11 | 0.00 | 0.11 | 0.11 | 0.00 | 0.12 | 0.12 | 0.00 | 0.10 | 0.10 | 0.00 |
| Weifang | 0.01 | 0.01 | 0.00 | 0.01 | 0.01 | 0.00 | 0.01 | 0.01 | 0.00 | 0.01 | 0.01 | 0.00 | 0.01 | 0.01 | 0.00 |
| Weihai | 0.05 | 0.05 | 0.00 | 0.05 | 0.05 | 0.00 | 0.06 | 0.06 | 0.00 | 0.06 | 0.06 | 0.00 | 0.06 | 0.06 | 0.00 |
| Yantai | 0.07 | 0.07 | 0.00 | 0.07 | 0.07 | 0.00 | 0.07 | 0.07 | 0.00 | 0.07 | 0.07 | 0.00 | 0.07 | 0.07 | 0.00 |
| Zaozhuang | 0.02 | 0.02 | 0.00 | 0.01 | 0.01 | 0.00 | 0.00 | 0.00 | 0.00 | 0.01 | 0.01 | 0.00 | 0.01 | 0.01 | 0.00 |
| Zibo | 0.11 | 0.11 | 0.00 | 0.10 | 0.10 | 0.00 | 0.10 | 0.10 | 0.00 | 0.10 | 0.10 | 0.00 | 0.10 | 0.10 | 0.00 |
| Marital status |  |  |  |  |  |  |  |  |  |  |  |  |  |  |  |
| Married | 0.02 | 0.05 | -0.27 | 0.02 | 0.02 | 0.01 | 0.02 | 0.02 | 0.01 | 0.02 | 0.02 | 0.01 | 0.02 | 0.02 | 0.00 |
| Single | 0.48 | 0.65 | -0.33 | 0.52 | 0.52 | 0.00 | 0.57 | 0.55 | 0.02 | 0.57 | 0.55 | 0.02 | 0.57 | 0.57 | -0.01 |
| Widowed/divorced | 0.01 | 0.01 | 0.03 | 0.01 | 0.01 | 0.03 | 0.01 | 0.01 | 0.01 | 0.01 | 0.01 | 0.03 | 0.01 | 0.01 | -0.01 |
| Unknown | 0.49 | 0.30 | 0.40 | 0.45 | 0.45 | -0.01 | 0.40 | 0.42 | -0.03 | 0.40 | 0.42 | -0.03 | 0.40 | 0.39 | 0.01 |
| Cancer type |  |  |  |  |  |  |  |  |  |  |  |  |  |  |  |
| Breast | 0.04 | 0.17 | -0.66 | 0.04 | 0.16 | -0.60 | 0.04 | 0.05 | -0.02 | 0.04 | 0.05 | -0.04 | 0.04 | 0.04 | -0.01 |
| Colorectum | 0.49 | 0.25 | 0.47 | 0.46 | 0.30 | 0.32 | 0.40 | 0.41 | -0.02 | 0.40 | 0.40 | -0.01 | 0.40 | 0.41 | -0.01 |
| Lung | 0.24 | 0.30 | -0.15 | 0.26 | 0.28 | -0.04 | 0.29 | 0.30 | -0.02 | 0.29 | 0.29 | -0.01 | 0.29 | 0.28 | 0.01 |
| Stomach | 0.13 | 0.17 | -0.14 | 0.13 | 0.16 | -0.08 | 0.15 | 0.14 | 0.03 | 0.15 | 0.14 | 0.02 | 0.15 | 0.16 | -0.02 |
| Thyroid | 0.11 | 0.11 | 0.00 | 0.11 | 0.11 | 0.00 | 0.12 | 0.11 | 0.04 | 0.12 | 0.12 | 0.03 | 0.12 | 0.11 | 0.04 |
| Hypertension |  |  |  |  |  |  |  |  |  |  |  |  |  |  |  |
| No | 0.98 | 0.97 | 0.11 | 0.98 | 0.97 | 0.08 | 0.98 | 0.98 | 0.02 | 0.98 | 0.98 | -0.01 | 0.98 | 0.99 | -0.03 |
| Yes | 0.02 | 0.03 | -0.11 | 0.02 | 0.03 | -0.08 | 0.02 | 0.02 | -0.02 | 0.02 | 0.02 | 0.01 | 0.02 | 0.01 | 0.03 |
| Diabetes |  |  |  |  |  |  |  |  |  |  |  |  |  |  |  |
| No | 0.99 | 0.97 | 0.14 | 0.99 | 0.98 | 0.11 | 0.99 | 0.97 | 0.15 | 0.99 | 0.99 | 0.01 | 0.99 | 0.99 | 0.01 |
| Yes | 0.01 | 0.03 | -0.14 | 0.01 | 0.02 | -0.11 | 0.01 | 0.03 | -0.15 | 0.01 | 0.01 | -0.01 | 0.01 | 0.01 | -0.01 |
| Heart |  |  |  |  |  |  |  |  |  |  |  |  |  |  |  |
| No | 0.95 | 0.93 | 0.11 | 0.95 | 0.94 | 0.06 | 0.95 | 0.95 | 0.03 | 0.95 | 0.95 | -0.03 | 0.95 | 0.96 | -0.04 |
| Yes | 0.05 | 0.07 | -0.11 | 0.05 | 0.06 | -0.06 | 0.05 | 0.05 | -0.03 | 0.05 | 0.05 | 0.03 | 0.05 | 0.04 | 0.04 |
| Osteoporosis |  |  |  |  |  |  |  |  |  |  |  |  |  |  |  |
| No | 0.80 | 0.76 | 0.11 | 0.80 | 0.74 | 0.13 | 0.81 | 0.76 | 0.13 | 0.80 | 0.79 | 0.01 | 0.78 | 0.80 | -0.05 |
| Yes | 0.20 | 0.24 | -0.11 | 0.20 | 0.26 | -0.13 | 0.19 | 0.24 | -0.13 | 0.20 | 0.21 | -0.01 | 0.22 | 0.20 | 0.05 |
| Cerebral |  |  |  |  |  |  |  |  |  |  |  |  |  |  |  |
| No | 0.96 | 0.94 | 0.10 | 0.96 | 0.94 | 0.07 | 0.96 | 0.94 | 0.09 | 0.95 | 0.95 | -0.01 | 0.95 | 0.96 | -0.03 |
| Yes | 0.04 | 0.06 | -0.10 | 0.04 | 0.06 | -0.07 | 0.04 | 0.06 | -0.09 | 0.05 | 0.05 | 0.01 | 0.05 | 0.04 | 0.03 |
| Comorbidity |  |  |  |  |  |  |  |  |  |  |  |  |  |  |  |
| No | 0.28 | 0.37 | -0.20 | 0.28 | 0.36 | -0.18 | 0.28 | 0.36 | -0.17 | 0.30 | 0.29 | 0.01 | 0.31 | 0.28 | 0.07 |
| Yes | 0.72 | 0.63 | 0.20 | 0.72 | 0.64 | 0.18 | 0.72 | 0.64 | 0.17 | 0.70 | 0.71 | -0.01 | 0.69 | 0.72 | -0.07 |
| Hospitalization frequency | 4.56 | 3.29 | 0.30 | 4.58 | 3.22 | 0.33 | 4.42 | 3.10 | 0.33 | 4.44 | 3.13 | 0.32 | 3.81 | 3.76 | 0.02 |
| Variables | National | Intra-city | SMD | National | Intra-city | SMD | National | Intra-city | SMD | National | Intra-city | SMD | National | Intra-city | SMD |
| Age group |  |  |  |  |  |  |  |  |  |  |  |  |  |  |  |
| Less40 | 0.11 | 0.11 | -0.01 | 0.11 | 0.10 | 0.03 | 0.11 | 0.09 | 0.05 | 0.11 | 0.10 | 0.03 | 0.11 | 0.09 | 0.06 |
| 40-50 | 0.27 | 0.27 | 0.01 | 0.27 | 0.28 | -0.02 | 0.26 | 0.25 | 0.02 | 0.26 | 0.26 | 0.00 | 0.25 | 0.24 | 0.02 |
| 50-60 | 0.26 | 0.26 | 0.00 | 0.26 | 0.26 | 0.00 | 0.26 | 0.27 | -0.02 | 0.26 | 0.24 | 0.04 | 0.26 | 0.26 | 0.00 |
| Greater60 | 0.36 | 0.36 | 0.00 | 0.36 | 0.36 | 0.00 | 0.37 | 0.39 | -0.04 | 0.37 | 0.40 | -0.06 | 0.38 | 0.41 | -0.06 |
| Sex |  |  |  |  |  |  |  |  |  |  |  |  |  |  |  |
| Male | 0.46 | 0.46 | 0.00 | 0.46 | 0.46 | 0.00 | 0.46 | 0.47 | -0.01 | 0.46 | 0.46 | 0.00 | 0.46 | 0.46 | 0.00 |
| Female | 0.54 | 0.54 | 0.00 | 0.54 | 0.54 | 0.00 | 0.54 | 0.53 | 0.01 | 0.54 | 0.54 | 0.00 | 0.54 | 0.54 | 0.00 |
| First hospitalization year |  |  |  |  |  |  |  |  |  |  |  |  |  |  |  |
| 2015 | 0.39 | 0.39 | -0.01 | 0.39 | 0.39 | 0.01 | 0.38 | 0.38 | 0.02 | 0.39 | 0.36 | 0.04 | 0.37 | 0.39 | -0.03 |
| 2016 | 0.37 | 0.37 | 0.00 | 0.36 | 0.38 | -0.03 | 0.37 | 0.37 | -0.01 | 0.37 | 0.38 | -0.02 | 0.37 | 0.37 | 0.00 |
| 2017 | 0.24 | 0.24 | 0.00 | 0.25 | 0.24 | 0.02 | 0.25 | 0.25 | -0.01 | 0.25 | 0.26 | -0.02 | 0.25 | 0.24 | 0.03 |
| City |  |  |  |  |  |  |  |  |  |  |  |  |  |  |  |
| Binzhou | 0.03 | 0.03 | 0.00 | 0.03 | 0.03 | 0.00 | 0.03 | 0.03 | 0.00 | 0.03 | 0.03 | 0.00 | 0.03 | 0.03 | 0.00 |
| Dezhou | 0.17 | 0.16 | 0.01 | 0.17 | 0.17 | 0.00 | 0.15 | 0.13 | 0.06 | 0.15 | 0.13 | 0.05 | 0.15 | 0.14 | 0.04 |
| Dongying | 0.22 | 0.22 | 0.00 | 0.22 | 0.22 | 0.00 | 0.22 | 0.22 | 0.00 | 0.22 | 0.22 | 0.00 | 0.22 | 0.21 | 0.02 |
| Heze | 0.02 | 0.01 | 0.02 | 0.01 | 0.01 | 0.03 | 0.02 | 0.02 | 0.03 | 0.02 | 0.02 | 0.03 | 0.02 | 0.01 | 0.02 |
| Jinan | 0.00 | 0.00 | 0.00 | 0.00 | 0.00 | 0.00 | 0.00 | 0.00 | 0.00 | 0.00 | 0.00 | 0.00 | 0.00 | 0.00 | 0.00 |
| Jining | 0.11 | 0.11 | 0.00 | 0.11 | 0.11 | 0.00 | 0.11 | 0.11 | -0.02 | 0.11 | 0.12 | -0.02 | 0.11 | 0.12 | -0.02 |
| Linyi | 0.08 | 0.08 | 0.00 | 0.08 | 0.08 | 0.00 | 0.08 | 0.08 | -0.01 | 0.08 | 0.08 | -0.01 | 0.08 | 0.09 | -0.01 |
| Qingdao | 0.00 | 0.00 | 0.00 | 0.00 | 0.00 | 0.00 | 0.00 | 0.00 | 0.00 | 0.00 | 0.00 | 0.00 | 0.00 | 0.00 | 0.00 |
| Taian | 0.03 | 0.03 | 0.00 | 0.03 | 0.03 | 0.00 | 0.03 | 0.03 | -0.01 | 0.03 | 0.03 | -0.01 | 0.03 | 0.03 | -0.01 |
| Weifang | 0.02 | 0.02 | 0.00 | 0.02 | 0.02 | 0.01 | 0.02 | 0.02 | 0.01 | 0.02 | 0.02 | 0.01 | 0.02 | 0.02 | 0.01 |
| Weihai | 0.18 | 0.18 | 0.00 | 0.18 | 0.18 | 0.00 | 0.19 | 0.19 | -0.02 | 0.19 | 0.19 | -0.02 | 0.18 | 0.19 | -0.02 |
| Yantai | 0.02 | 0.02 | 0.00 | 0.02 | 0.02 | 0.00 | 0.02 | 0.02 | -0.01 | 0.02 | 0.02 | -0.01 | 0.02 | 0.02 | -0.01 |
| Zibo | 0.13 | 0.13 | 0.00 | 0.13 | 0.13 | 0.00 | 0.14 | 0.14 | -0.02 | 0.14 | 0.14 | -0.02 | 0.13 | 0.14 | -0.02 |
| Marital status |  |  |  |  |  |  |  |  |  |  |  |  |  |  |  |
| Married | 0.65 | 0.64 | 0.03 | 0.65 | 0.66 | -0.02 | 0.66 | 0.65 | 0.01 | 0.66 | 0.66 | 0.00 | 0.66 | 0.68 | -0.03 |
| Single | 0.02 | 0.02 | -0.01 | 0.02 | 0.01 | 0.01 | 0.01 | 0.02 | -0.05 | 0.01 | 0.02 | -0.05 | 0.02 | 0.01 | 0.06 |
| Widowed/divorced | 0.01 | 0.03 | -0.23 | 0.01 | 0.01 | 0.00 | 0.01 | 0.01 | 0.03 | 0.01 | 0.00 | 0.08 | 0.01 | 0.01 | 0.05 |
| Unknown | 0.32 | 0.32 | 0.02 | 0.32 | 0.31 | 0.02 | 0.32 | 0.32 | 0.00 | 0.32 | 0.32 | 0.00 | 0.31 | 0.31 | 0.01 |
| Cancer type |  |  |  |  |  |  |  |  |  |  |  |  |  |  |  |
| Breast | 0.13 | 0.25 | -0.37 | 0.13 | 0.25 | -0.37 | 0.13 | 0.14 | -0.03 | 0.14 | 0.14 | -0.02 | 0.14 | 0.14 | 0.00 |
| Colorectum | 0.19 | 0.20 | -0.02 | 0.20 | 0.19 | 0.00 | 0.20 | 0.20 | -0.01 | 0.20 | 0.22 | -0.04 | 0.20 | 0.22 | -0.04 |
| Lung | 0.29 | 0.26 | 0.06 | 0.28 | 0.25 | 0.07 | 0.29 | 0.30 | -0.01 | 0.29 | 0.31 | -0.03 | 0.30 | 0.27 | 0.06 |
| Stomach | 0.15 | 0.15 | 0.01 | 0.15 | 0.14 | 0.02 | 0.15 | 0.16 | -0.01 | 0.16 | 0.14 | 0.04 | 0.15 | 0.17 | -0.08 |
| Thyroid | 0.24 | 0.14 | 0.23 | 0.24 | 0.16 | 0.20 | 0.22 | 0.20 | 0.05 | 0.21 | 0.19 | 0.05 | 0.22 | 0.20 | 0.04 |
| Hypertension |  |  |  |  |  |  |  |  |  |  |  |  |  |  |  |
| No | 0.99 | 0.97 | 0.23 | 0.99 | 0.97 | 0.20 | 0.99 | 0.98 | 0.11 | 0.99 | 0.99 | -0.03 | 0.99 | 0.99 | 0.04 |
| Yes | 0.01 | 0.03 | -0.23 | 0.01 | 0.03 | -0.20 | 0.01 | 0.02 | -0.11 | 0.01 | 0.01 | 0.03 | 0.01 | 0.01 | -0.04 |
| Diabetes |  |  |  |  |  |  |  |  |  |  |  |  |  |  |  |
| No | 0.98 | 0.97 | 0.02 | 0.98 | 0.97 | 0.03 | 0.98 | 0.98 | 0.00 | 0.98 | 0.99 | -0.06 | 0.98 | 0.98 | -0.05 |
| Yes | 0.02 | 0.03 | -0.02 | 0.02 | 0.03 | -0.03 | 0.02 | 0.02 | 0.00 | 0.02 | 0.01 | 0.06 | 0.02 | 0.02 | 0.05 |
| Heart |  |  |  |  |  |  |  |  |  |  |  |  |  |  |  |
| No | 0.97 | 0.94 | 0.16 | 0.97 | 0.95 | 0.13 | 0.97 | 0.94 | 0.16 | 0.97 | 0.96 | 0.06 | 0.97 | 0.97 | 0.00 |
| Yes | 0.03 | 0.06 | -0.16 | 0.03 | 0.05 | -0.13 | 0.03 | 0.06 | -0.16 | 0.03 | 0.04 | -0.06 | 0.03 | 0.03 | 0.00 |
| Osteoporosis |  |  |  |  |  |  |  |  |  |  |  |  |  |  |  |
| No | 0.80 | 0.80 | 0.00 | 0.80 | 0.80 | 0.01 | 0.80 | 0.80 | -0.02 | 0.80 | 0.81 | -0.03 | 0.81 | 0.81 | -0.01 |
| Yes | 0.20 | 0.20 | 0.00 | 0.20 | 0.20 | -0.01 | 0.20 | 0.20 | 0.02 | 0.20 | 0.19 | 0.03 | 0.19 | 0.19 | 0.01 |
| Cerebral |  |  |  |  |  |  |  |  |  |  |  |  |  |  |  |
| No | 0.98 | 0.95 | 0.22 | 0.98 | 0.95 | 0.21 | 0.98 | 0.95 | 0.22 | 0.98 | 0.98 | -0.02 | 0.98 | 0.98 | -0.01 |
| Yes | 0.02 | 0.05 | -0.22 | 0.02 | 0.05 | -0.21 | 0.02 | 0.05 | -0.22 | 0.02 | 0.02 | 0.02 | 0.02 | 0.02 | 0.01 |
| Comorbidity |  |  |  |  |  |  |  |  |  |  |  |  |  |  |  |
| No | 0.74 | 0.69 | 0.11 | 0.74 | 0.69 | 0.12 | 0.74 | 0.70 | 0.09 | 0.74 | 0.75 | -0.03 | 0.75 | 0.75 | -0.01 |
| Yes | 0.26 | 0.31 | -0.11 | 0.26 | 0.31 | -0.12 | 0.26 | 0.30 | -0.09 | 0.26 | 0.25 | 0.03 | 0.25 | 0.25 | 0.01 |
| Hospitalization frequency | 3.69 | 3.31 | 0.10 | 3.70 | 3.25 | 0.12 | 3.75 | 3.09 | 0.17 | 3.77 | 3.19 | 0.15 | 3.62 | 3.43 | 0.05 |

Local, local center pattern; National, National center pattern; SMD, Standardized mean difference.

# Supplementary Table 6. Matching results for patients with uncommon cancer

|  | Demographic matched | | | Marriage matched | | | Cancer matched | | | Morbidity matched | | | Frequency matched | | |
| --- | --- | --- | --- | --- | --- | --- | --- | --- | --- | --- | --- | --- | --- | --- | --- |
| Variables | Local | Intra-city | SMD | Local | Intra-city | SMD | Local | Intra-city | SMD | Local | Intra-city | SMD | Local | Intra-city | SMD |
| Age group |  |  |  |  |  |  |  |  |  |  |  |  |  |  |  |
| Less40 | 0.10 | 0.09 | 0.01 | 0.09 | 0.08 | 0.00 | 0.08 | 0.07 | 0.04 | 0.09 | 0.08 | 0.03 | 0.08 | 0.08 | 0.01 |
| 40-50 | 0.14 | 0.15 | -0.03 | 0.15 | 0.15 | -0.02 | 0.14 | 0.14 | 0.02 | 0.15 | 0.14 | 0.01 | 0.15 | 0.14 | 0.02 |
| 50-60 | 0.28 | 0.27 | 0.02 | 0.28 | 0.27 | 0.01 | 0.28 | 0.26 | 0.04 | 0.28 | 0.27 | 0.03 | 0.26 | 0.27 | -0.03 |
| Greater60 | 0.49 | 0.49 | 0.00 | 0.49 | 0.49 | 0.01 | 0.49 | 0.53 | -0.07 | 0.49 | 0.51 | -0.05 | 0.51 | 0.51 | 0.01 |
| Sex |  |  |  |  |  |  |  |  |  |  |  |  |  |  |  |
| Male | 0.59 | 0.59 | 0.00 | 0.59 | 0.59 | 0.00 | 0.60 | 0.60 | 0.00 | 0.60 | 0.60 | 0.00 | 0.60 | 0.60 | 0.00 |
| Female | 0.41 | 0.41 | 0.00 | 0.41 | 0.41 | 0.00 | 0.40 | 0.40 | 0.00 | 0.40 | 0.40 | 0.00 | 0.40 | 0.40 | 0.00 |
| First hospitalization year |  |  |  |  |  |  |  |  |  |  |  |  |  |  |  |
| 2015 | 0.30 | 0.33 | -0.07 | 0.31 | 0.33 | -0.04 | 0.32 | 0.32 | 0.01 | 0.33 | 0.32 | 0.02 | 0.33 | 0.30 | 0.07 |
| 2016 | 0.36 | 0.36 | 0.00 | 0.37 | 0.36 | 0.02 | 0.36 | 0.37 | -0.03 | 0.36 | 0.37 | -0.04 | 0.35 | 0.37 | -0.04 |
| 2017 | 0.35 | 0.31 | 0.07 | 0.32 | 0.31 | 0.02 | 0.32 | 0.31 | 0.02 | 0.31 | 0.30 | 0.02 | 0.32 | 0.34 | -0.03 |
| City |  |  |  |  |  |  |  |  |  |  |  |  |  |  |  |
| Binzhou | 0.05 | 0.05 | 0.00 | 0.05 | 0.05 | 0.00 | 0.05 | 0.05 | 0.00 | 0.05 | 0.05 | 0.00 | 0.05 | 0.05 | 0.00 |
| Dezhou | 0.13 | 0.13 | 0.00 | 0.13 | 0.13 | 0.00 | 0.13 | 0.13 | 0.00 | 0.13 | 0.13 | 0.00 | 0.13 | 0.13 | 0.00 |
| Dongying | 0.18 | 0.18 | 0.00 | 0.18 | 0.18 | 0.00 | 0.18 | 0.18 | 0.00 | 0.18 | 0.18 | 0.00 | 0.19 | 0.19 | 0.00 |
| Heze | 0.12 | 0.12 | 0.00 | 0.12 | 0.12 | 0.00 | 0.11 | 0.11 | 0.00 | 0.11 | 0.11 | 0.00 | 0.11 | 0.11 | 0.00 |
| Jinan | 0.01 | 0.01 | 0.00 | 0.01 | 0.01 | 0.00 | 0.01 | 0.01 | 0.00 | 0.01 | 0.01 | 0.00 | 0.01 | 0.01 | 0.00 |
| Jining | 0.12 | 0.12 | 0.00 | 0.12 | 0.12 | 0.00 | 0.12 | 0.12 | 0.00 | 0.12 | 0.12 | 0.00 | 0.12 | 0.12 | 0.00 |
| Liaocheng | 0.03 | 0.03 | 0.00 | 0.03 | 0.03 | 0.00 | 0.03 | 0.03 | 0.00 | 0.03 | 0.03 | 0.00 | 0.03 | 0.03 | 0.00 |
| Linyi | 0.04 | 0.04 | 0.00 | 0.04 | 0.04 | 0.00 | 0.04 | 0.04 | 0.00 | 0.04 | 0.04 | 0.00 | 0.04 | 0.04 | 0.00 |
| Qingdao | 0.01 | 0.01 | 0.00 | 0.01 | 0.01 | 0.00 | 0.01 | 0.01 | 0.00 | 0.01 | 0.01 | 0.00 | 0.01 | 0.01 | 0.00 |
| Taian | 0.07 | 0.07 | 0.00 | 0.08 | 0.08 | 0.00 | 0.08 | 0.08 | 0.00 | 0.08 | 0.08 | 0.00 | 0.08 | 0.08 | 0.00 |
| Weifang | 0.01 | 0.01 | 0.00 | 0.01 | 0.01 | 0.00 | 0.01 | 0.01 | 0.00 | 0.01 | 0.01 | 0.00 | 0.01 | 0.01 | 0.00 |
| Weihai | 0.05 | 0.05 | 0.00 | 0.05 | 0.05 | 0.00 | 0.05 | 0.05 | 0.00 | 0.05 | 0.05 | 0.00 | 0.05 | 0.05 | 0.00 |
| Yantai | 0.05 | 0.05 | 0.00 | 0.05 | 0.05 | 0.00 | 0.05 | 0.05 | 0.00 | 0.05 | 0.05 | 0.00 | 0.05 | 0.05 | 0.00 |
| Zaozhuang | 0.06 | 0.06 | 0.00 | 0.06 | 0.06 | 0.00 | 0.06 | 0.06 | 0.00 | 0.06 | 0.06 | 0.00 | 0.04 | 0.04 | 0.00 |
| Zibo | 0.06 | 0.06 | 0.00 | 0.06 | 0.06 | 0.00 | 0.06 | 0.06 | 0.00 | 0.06 | 0.06 | 0.00 | 0.07 | 0.07 | 0.00 |
| Marital status |  |  |  |  |  |  |  |  |  |  |  |  |  |  |  |
| Married | 0.72 | 0.70 | 0.05 | 0.75 | 0.72 | 0.08 | 0.73 | 0.75 | -0.04 | 0.72 | 0.74 | -0.05 | 0.73 | 0.72 | 0.02 |
| Single | 0.04 | 0.02 | 0.07 | 0.02 | 0.02 | 0.03 | 0.02 | 0.02 | 0.01 | 0.03 | 0.02 | 0.02 | 0.03 | 0.02 | 0.01 |
| Widowed/divorced | 0.03 | 0.04 | -0.04 | 0.02 | 0.03 | -0.02 | 0.03 | 0.02 | 0.02 | 0.03 | 0.02 | 0.02 | 0.03 | 0.03 | -0.02 |
| Unknown | 0.21 | 0.24 | -0.07 | 0.21 | 0.24 | -0.08 | 0.22 | 0.21 | 0.03 | 0.23 | 0.21 | 0.04 | 0.21 | 0.22 | -0.02 |
| Cancer type |  |  |  |  |  |  |  |  |  |  |  |  |  |  |  |
| Bladder | 0.04 | 0.07 | -0.15 | 0.05 | 0.07 | -0.12 | 0.04 | 0.05 | -0.02 | 0.04 | 0.05 | -0.01 | 0.04 | 0.04 | 0.04 |
| Brain | 0.03 | 0.05 | -0.14 | 0.03 | 0.04 | -0.08 | 0.02 | 0.02 | 0.02 | 0.03 | 0.02 | 0.04 | 0.03 | 0.02 | 0.05 |
| Cervix | 0.16 | 0.12 | 0.10 | 0.16 | 0.12 | 0.09 | 0.15 | 0.16 | -0.01 | 0.15 | 0.16 | 0.00 | 0.15 | 0.14 | 0.04 |
| Gallbladder | 0.03 | 0.03 | 0.03 | 0.03 | 0.03 | 0.03 | 0.03 | 0.03 | 0.01 | 0.04 | 0.03 | 0.01 | 0.03 | 0.03 | 0.02 |
| Kidney | 0.08 | 0.06 | 0.06 | 0.08 | 0.07 | 0.04 | 0.08 | 0.09 | -0.03 | 0.09 | 0.09 | -0.03 | 0.08 | 0.09 | -0.03 |
| Larynx | 0.01 | 0.02 | -0.03 | 0.01 | 0.01 | -0.01 | 0.02 | 0.01 | 0.03 | 0.01 | 0.01 | 0.03 | 0.01 | 0.01 | 0.00 |
| Leukemia | 0.04 | 0.07 | -0.12 | 0.04 | 0.07 | -0.14 | 0.05 | 0.04 | 0.03 | 0.05 | 0.05 | -0.01 | 0.04 | 0.05 | -0.06 |
| Liver | 0.17 | 0.17 | 0.00 | 0.17 | 0.18 | -0.04 | 0.19 | 0.18 | 0.01 | 0.18 | 0.18 | 0.00 | 0.18 | 0.18 | -0.01 |
| Lymphoma | 0.11 | 0.11 | -0.03 | 0.11 | 0.11 | 0.02 | 0.11 | 0.11 | 0.00 | 0.11 | 0.11 | -0.01 | 0.11 | 0.13 | -0.06 |
| Melanoma | 0.02 | 0.00 | 0.10 | 0.02 | 0.00 | 0.11 | 0.01 | 0.01 | 0.02 | 0.01 | 0.01 | 0.00 | 0.01 | 0.00 | 0.05 |
| Nasopharynx | 0.03 | 0.02 | 0.07 | 0.03 | 0.02 | 0.06 | 0.02 | 0.02 | 0.02 | 0.02 | 0.01 | 0.05 | 0.02 | 0.02 | 0.04 |
| Esophagus | 0.16 | 0.13 | 0.08 | 0.16 | 0.13 | 0.07 | 0.16 | 0.17 | -0.02 | 0.16 | 0.17 | -0.04 | 0.17 | 0.16 | 0.02 |
| Oral | 0.04 | 0.04 | -0.01 | 0.04 | 0.04 | -0.02 | 0.04 | 0.05 | -0.06 | 0.04 | 0.04 | 0.01 | 0.04 | 0.05 | -0.05 |
| Other skin | 0.01 | 0.02 | -0.05 | 0.01 | 0.02 | -0.08 | 0.01 | 0.01 | 0.05 | 0.01 | 0.01 | 0.05 | 0.01 | 0.01 | 0.04 |
| Other thoracic | 0.01 | 0.01 | 0.05 | 0.01 | 0.01 | 0.04 | 0.01 | 0.01 | -0.03 | 0.01 | 0.01 | 0.00 | 0.01 | 0.01 | -0.03 |
| Pancreas | 0.02 | 0.04 | -0.08 | 0.02 | 0.03 | -0.09 | 0.03 | 0.02 | 0.02 | 0.02 | 0.03 | -0.02 | 0.02 | 0.02 | -0.02 |
| Prostate | 0.04 | 0.04 | -0.04 | 0.03 | 0.04 | -0.03 | 0.04 | 0.03 | 0.02 | 0.04 | 0.03 | 0.03 | 0.04 | 0.03 | 0.01 |
| Hypertension |  |  |  |  |  |  |  |  |  |  |  |  |  |  |  |
| No | 0.98 | 0.97 | 0.07 | 0.98 | 0.97 | 0.04 | 0.98 | 0.98 | 0.04 | 0.98 | 0.98 | 0.00 | 0.98 | 0.98 | 0.01 |
| Yes | 0.02 | 0.03 | -0.07 | 0.02 | 0.03 | -0.04 | 0.02 | 0.02 | -0.04 | 0.02 | 0.02 | 0.00 | 0.02 | 0.02 | -0.01 |
| Diabetes |  |  |  |  |  |  |  |  |  |  |  |  |  |  |  |
| No | 0.98 | 0.98 | 0.04 | 0.98 | 0.98 | 0.03 | 0.98 | 0.98 | 0.00 | 0.98 | 0.99 | -0.04 | 0.98 | 0.99 | -0.04 |
| Yes | 0.02 | 0.02 | -0.04 | 0.02 | 0.02 | -0.03 | 0.02 | 0.02 | 0.00 | 0.01 | 0.01 | 0.04 | 0.02 | 0.01 | 0.04 |
| Heart |  |  |  |  |  |  |  |  |  |  |  |  |  |  |  |
| No | 0.97 | 0.93 | 0.25 | 0.97 | 0.94 | 0.21 | 0.97 | 0.94 | 0.21 | 0.97 | 0.97 | -0.01 | 0.97 | 0.97 | -0.02 |
| Yes | 0.03 | 0.07 | -0.25 | 0.03 | 0.06 | -0.21 | 0.03 | 0.06 | -0.21 | 0.03 | 0.03 | 0.01 | 0.03 | 0.03 | 0.02 |
| Osteoporosis |  |  |  |  |  |  |  |  |  |  |  |  |  |  |  |
| No | 0.82 | 0.79 | 0.08 | 0.82 | 0.79 | 0.07 | 0.81 | 0.81 | 0.02 | 0.83 | 0.81 | 0.05 | 0.82 | 0.80 | 0.04 |
| Yes | 0.18 | 0.21 | -0.08 | 0.18 | 0.21 | -0.07 | 0.19 | 0.19 | -0.02 | 0.17 | 0.19 | -0.05 | 0.18 | 0.20 | -0.04 |
| Cerebral |  |  |  |  |  |  |  |  |  |  |  |  |  |  |  |
| No | 0.95 | 0.95 | 0.01 | 0.95 | 0.95 | 0.01 | 0.95 | 0.94 | 0.02 | 0.96 | 0.96 | -0.05 | 0.95 | 0.95 | -0.02 |
| Yes | 0.05 | 0.05 | -0.01 | 0.05 | 0.05 | -0.01 | 0.05 | 0.06 | -0.02 | 0.04 | 0.04 | 0.05 | 0.05 | 0.05 | 0.02 |
| Comorbidity |  |  |  |  |  |  |  |  |  |  |  |  |  |  |  |
| No | 0.74 | 0.68 | 0.12 | 0.73 | 0.69 | 0.09 | 0.73 | 0.70 | 0.07 | 0.75 | 0.74 | 0.01 | 0.73 | 0.72 | 0.02 |
| Yes | 0.26 | 0.32 | -0.12 | 0.27 | 0.31 | -0.09 | 0.27 | 0.30 | -0.07 | 0.25 | 0.26 | -0.01 | 0.27 | 0.28 | -0.02 |
| Hospitalization frequency | 3.50 | 2.72 | 0.25 | 3.51 | 2.70 | 0.26 | 3.50 | 2.68 | 0.26 | 3.51 | 2.84 | 0.21 | 3.25 | 3.11 | 0.05 |
| Variables | National | Intra-city | SMD | National | Intra-city | SMD | National | Intra-city | SMD | National | Intra-city | SMD | National | Intra-city | SMD |
| Age group |  |  |  |  |  |  |  |  |  |  |  |  |  |  |  |
| Less40 | 0.12 | 0.09 | 0.09 | 0.08 | 0.06 | 0.09 | 0.10 | 0.08 | 0.08 | 0.09 | 0.07 | 0.08 | 0.07 | 0.07 | 0.02 |
| 40-50 | 0.20 | 0.19 | 0.00 | 0.20 | 0.19 | 0.05 | 0.17 | 0.15 | 0.05 | 0.18 | 0.13 | 0.13 | 0.17 | 0.16 | 0.01 |
| 50-60 | 0.24 | 0.26 | -0.03 | 0.24 | 0.28 | -0.08 | 0.24 | 0.22 | 0.05 | 0.24 | 0.22 | 0.04 | 0.25 | 0.24 | 0.01 |
| Greater60 | 0.44 | 0.46 | -0.04 | 0.47 | 0.48 | -0.01 | 0.49 | 0.55 | -0.13 | 0.49 | 0.58 | -0.18 | 0.51 | 0.53 | -0.03 |
| Sex |  |  |  |  |  |  |  |  |  |  |  |  |  |  |  |
| Male | 0.75 | 0.75 | 0.00 | 0.75 | 0.75 | -0.01 | 0.75 | 0.75 | 0.01 | 0.74 | 0.74 | 0.01 | 0.76 | 0.76 | 0.00 |
| Female | 0.25 | 0.25 | 0.00 | 0.25 | 0.25 | 0.01 | 0.25 | 0.25 | -0.01 | 0.26 | 0.26 | -0.01 | 0.24 | 0.24 | 0.00 |
| First hospitalization year |  |  |  |  |  |  |  |  |  |  |  |  |  |  |  |
| 2015 | 0.36 | 0.36 | 0.00 | 0.34 | 0.35 | -0.04 | 0.37 | 0.38 | -0.02 | 0.35 | 0.35 | 0.00 | 0.35 | 0.36 | -0.02 |
| 2016 | 0.43 | 0.43 | -0.01 | 0.44 | 0.43 | 0.02 | 0.41 | 0.41 | 0.00 | 0.43 | 0.40 | 0.04 | 0.41 | 0.38 | 0.06 |
| 2017 | 0.21 | 0.21 | 0.01 | 0.22 | 0.22 | 0.01 | 0.22 | 0.21 | 0.02 | 0.23 | 0.25 | -0.05 | 0.24 | 0.26 | -0.05 |
| City |  |  |  |  |  |  |  |  |  |  |  |  |  |  |  |
| Binzhou | 0.02 | 0.02 | -0.01 | 0.01 | 0.01 | -0.01 | 0.01 | 0.01 | -0.01 | 0.01 | 0.01 | 0.01 | 0.01 | 0.01 | -0.01 |
| Dezhou | 0.06 | 0.06 | 0.01 | 0.05 | 0.05 | 0.01 | 0.05 | 0.05 | 0.01 | 0.06 | 0.05 | 0.02 | 0.06 | 0.06 | 0.03 |
| Dongying | 0.22 | 0.21 | 0.02 | 0.22 | 0.21 | 0.02 | 0.23 | 0.22 | 0.03 | 0.21 | 0.21 | 0.00 | 0.21 | 0.20 | 0.02 |
| Heze | 0.10 | 0.09 | 0.01 | 0.08 | 0.08 | 0.01 | 0.07 | 0.06 | 0.01 | 0.07 | 0.06 | 0.06 | 0.07 | 0.05 | 0.06 |
| Jining | 0.19 | 0.19 | 0.01 | 0.20 | 0.20 | 0.01 | 0.20 | 0.20 | 0.00 | 0.20 | 0.20 | 0.01 | 0.20 | 0.20 | 0.01 |
| Linyi | 0.08 | 0.09 | -0.01 | 0.08 | 0.09 | -0.01 | 0.08 | 0.09 | -0.02 | 0.09 | 0.09 | -0.01 | 0.08 | 0.08 | 0.00 |
| Qingdao | 0.01 | 0.01 | 0.00 | 0.01 | 0.01 | 0.00 | 0.01 | 0.01 | -0.01 | 0.01 | 0.01 | -0.01 | 0.01 | 0.01 | -0.01 |
| Taian | 0.06 | 0.06 | 0.00 | 0.05 | 0.05 | 0.00 | 0.06 | 0.06 | -0.01 | 0.05 | 0.06 | -0.02 | 0.06 | 0.06 | -0.02 |
| Weifang | 0.01 | 0.01 | 0.02 | 0.01 | 0.01 | 0.00 | 0.01 | 0.00 | 0.04 | 0.00 | 0.00 | -0.01 | 0.01 | 0.01 | -0.01 |
| Weihai | 0.14 | 0.14 | -0.02 | 0.15 | 0.16 | -0.02 | 0.15 | 0.17 | -0.04 | 0.16 | 0.17 | -0.03 | 0.17 | 0.18 | -0.05 |
| Yantai | 0.00 | 0.00 | 0.00 | 0.00 | 0.00 | 0.00 | 0.00 | 0.00 | -0.01 | 0.00 | 0.00 | -0.01 | 0.00 | 0.01 | -0.01 |
| Zaozhuang | 0.01 | 0.01 | 0.00 | 0.01 | 0.01 | 0.00 | 0.01 | 0.01 | 0.02 | 0.01 | 0.01 | 0.02 | 0.01 | 0.01 | -0.01 |
| Zibo | 0.12 | 0.12 | -0.02 | 0.12 | 0.12 | -0.01 | 0.12 | 0.12 | 0.00 | 0.11 | 0.12 | -0.01 | 0.12 | 0.12 | -0.01 |
| Marital status |  |  |  |  |  |  |  |  |  |  |  |  |  |  |  |
| Married | 0.60 | 0.64 | -0.09 | 0.63 | 0.66 | -0.06 | 0.62 | 0.64 | -0.03 | 0.62 | 0.62 | 0.02 | 0.62 | 0.64 | -0.05 |
| Single | 0.07 | 0.02 | 0.18 | 0.03 | 0.02 | 0.08 | 0.03 | 0.02 | 0.08 | 0.01 | 0.02 | -0.03 | 0.01 | 0.02 | -0.06 |
| Unknown | 0.34 | 0.32 | 0.05 | 0.34 | 0.33 | 0.03 | 0.35 | 0.35 | 0.00 | 0.36 | 0.37 | -0.01 | 0.37 | 0.34 | 0.06 |
| Cancer type |  |  |  |  |  |  |  |  |  |  |  |  |  |  |  |
| Bladder | 0.03 | 0.09 | -0.33 | 0.03 | 0.08 | -0.24 | 0.03 | 0.03 | 0.05 | 0.04 | 0.04 | -0.03 | 0.04 | 0.05 | -0.05 |
| Brain | 0.07 | 0.08 | -0.03 | 0.07 | 0.07 | 0.03 | 0.07 | 0.07 | 0.01 | 0.07 | 0.09 | -0.06 | 0.07 | 0.08 | -0.03 |
| Cervix | 0.04 | 0.07 | -0.18 | 0.04 | 0.08 | -0.23 | 0.05 | 0.06 | -0.04 | 0.05 | 0.05 | -0.01 | 0.05 | 0.06 | -0.07 |
| Gallbladder | 0.04 | 0.03 | 0.05 | 0.05 | 0.03 | 0.07 | 0.05 | 0.02 | 0.11 | 0.05 | 0.03 | 0.07 | 0.05 | 0.02 | 0.12 |
| Kidney | 0.06 | 0.08 | -0.10 | 0.06 | 0.09 | -0.13 | 0.06 | 0.07 | -0.03 | 0.07 | 0.05 | 0.05 | 0.07 | 0.06 | 0.06 |
| Leukemia | 0.13 | 0.09 | 0.12 | 0.11 | 0.08 | 0.09 | 0.12 | 0.08 | 0.12 | 0.10 | 0.09 | 0.00 | 0.08 | 0.09 | -0.04 |
| Liver | 0.22 | 0.16 | 0.13 | 0.23 | 0.17 | 0.14 | 0.22 | 0.27 | -0.11 | 0.21 | 0.25 | -0.10 | 0.21 | 0.25 | -0.08 |
| Lymphoma | 0.16 | 0.11 | 0.12 | 0.15 | 0.11 | 0.11 | 0.14 | 0.15 | -0.03 | 0.15 | 0.12 | 0.09 | 0.14 | 0.12 | 0.06 |
| Nasopharynx | 0.02 | 0.01 | 0.09 | 0.03 | 0.01 | 0.08 | 0.02 | 0.02 | -0.01 | 0.02 | 0.02 | -0.01 | 0.03 | 0.02 | 0.08 |
| Esophagus | 0.08 | 0.13 | -0.17 | 0.09 | 0.15 | -0.21 | 0.09 | 0.10 | -0.01 | 0.09 | 0.09 | 0.01 | 0.10 | 0.11 | -0.04 |
| Oral | 0.03 | 0.05 | -0.06 | 0.04 | 0.04 | -0.03 | 0.04 | 0.04 | 0.01 | 0.04 | 0.03 | 0.03 | 0.04 | 0.04 | 0.02 |
| Pancreas | 0.04 | 0.04 | -0.01 | 0.04 | 0.04 | 0.00 | 0.04 | 0.03 | 0.05 | 0.04 | 0.03 | 0.03 | 0.05 | 0.04 | 0.05 |
| Prostate | 0.07 | 0.05 | 0.08 | 0.07 | 0.05 | 0.08 | 0.07 | 0.07 | -0.03 | 0.07 | 0.08 | -0.04 | 0.08 | 0.08 | -0.02 |
| Hypertension |  |  |  |  |  |  |  |  |  |  |  |  |  |  |  |
| No | 0.98 | 0.96 | 0.14 | 0.98 | 0.97 | 0.08 | 0.98 | 0.98 | 0.00 | 0.98 | 0.99 | -0.05 | 0.98 | 0.98 | -0.04 |
| Yes | 0.02 | 0.04 | -0.14 | 0.02 | 0.03 | -0.08 | 0.02 | 0.02 | 0.00 | 0.02 | 0.01 | 0.05 | 0.02 | 0.02 | 0.04 |
| Diabetes |  |  |  |  |  |  |  |  |  |  |  |  |  |  |  |
| No | 0.97 | 0.98 | -0.07 | 0.96 | 0.97 | -0.05 | 0.97 | 0.98 | -0.08 | 0.97 | 0.97 | 0.01 | 0.97 | 0.97 | -0.01 |
| Yes | 0.03 | 0.02 | 0.07 | 0.04 | 0.03 | 0.05 | 0.03 | 0.02 | 0.08 | 0.03 | 0.03 | -0.01 | 0.03 | 0.03 | 0.01 |
| Heart |  |  |  |  |  |  |  |  |  |  |  |  |  |  |  |
| No | 0.95 | 0.93 | 0.08 | 0.95 | 0.93 | 0.09 | 0.96 | 0.95 | 0.03 | 0.95 | 0.95 | 0.00 | 0.95 | 0.96 | -0.05 |
| Yes | 0.05 | 0.07 | -0.08 | 0.05 | 0.07 | -0.09 | 0.04 | 0.05 | -0.03 | 0.05 | 0.05 | 0.00 | 0.05 | 0.04 | 0.05 |
| Osteoporosis |  |  |  |  |  |  |  |  |  |  |  |  |  |  |  |
| No | 0.80 | 0.84 | -0.09 | 0.80 | 0.84 | -0.11 | 0.81 | 0.84 | -0.08 | 0.84 | 0.83 | 0.03 | 0.82 | 0.84 | -0.07 |
| Yes | 0.20 | 0.16 | 0.09 | 0.20 | 0.16 | 0.11 | 0.19 | 0.16 | 0.08 | 0.16 | 0.17 | -0.03 | 0.18 | 0.16 | 0.07 |
| Cerebral |  |  |  |  |  |  |  |  |  |  |  |  |  |  |  |
| No | 0.97 | 0.93 | 0.18 | 0.96 | 0.93 | 0.16 | 0.97 | 0.95 | 0.11 | 0.96 | 0.96 | -0.02 | 0.96 | 0.96 | 0.04 |
| Yes | 0.03 | 0.07 | -0.18 | 0.04 | 0.07 | -0.16 | 0.03 | 0.05 | -0.11 | 0.04 | 0.04 | 0.02 | 0.04 | 0.04 | -0.04 |
| Comorbidity |  |  |  |  |  |  |  |  |  |  |  |  |  |  |  |
| No | 0.72 | 0.70 | 0.05 | 0.71 | 0.69 | 0.05 | 0.73 | 0.74 | -0.04 | 0.74 | 0.75 | -0.02 | 0.72 | 0.75 | -0.08 |
| Yes | 0.28 | 0.30 | -0.05 | 0.29 | 0.31 | -0.05 | 0.27 | 0.26 | 0.04 | 0.26 | 0.25 | 0.02 | 0.28 | 0.25 | 0.08 |
| Hospitalization frequency | 3.92 | 3.16 | 0.20 | 3.83 | 3.33 | 0.14 | 3.92 | 3.38 | 0.14 | 3.86 | 3.23 | 0.17 | 3.41 | 3.18 | 0.07 |

Local, local center pattern; National, National center pattern; SMD, Standardized mean difference.

# Supplementary Table 7. Matching results for cancer patients in cities with healthcare resources more than average level

|  | Demographic matched | | | Marriage matched | | | Cancer matched | | | Morbidity matched | | | Frequency matched | | |
| --- | --- | --- | --- | --- | --- | --- | --- | --- | --- | --- | --- | --- | --- | --- | --- |
| Variables | Local | Intra-city | SMD | Local | Intra-city | SMD | Local | Intra-city | SMD | Local | Intra-city | SMD | Local | Intra-city | SMD |
| Age group |  |  |  |  |  |  |  |  |  |  |  |  |  |  |  |
| Less40 | 0.06 | 0.06 | 0.00 | 0.06 | 0.06 | -0.02 | 0.06 | 0.05 | 0.04 | 0.06 | 0.05 | 0.04 | 0.06 | 0.05 | 0.07 |
| 40-50 | 0.15 | 0.14 | 0.02 | 0.14 | 0.15 | -0.01 | 0.14 | 0.13 | 0.03 | 0.14 | 0.14 | 0.00 | 0.14 | 0.14 | 0.01 |
| 50-60 | 0.26 | 0.26 | -0.01 | 0.26 | 0.26 | 0.01 | 0.26 | 0.27 | -0.01 | 0.27 | 0.27 | -0.01 | 0.25 | 0.26 | -0.03 |
| Greater60 | 0.53 | 0.53 | 0.00 | 0.54 | 0.53 | 0.01 | 0.54 | 0.55 | -0.03 | 0.54 | 0.54 | -0.01 | 0.55 | 0.56 | -0.02 |
| Sex |  |  |  |  |  |  |  |  |  |  |  |  |  |  |  |
| Male | 0.60 | 0.60 | 0.00 | 0.59 | 0.59 | 0.00 | 0.60 | 0.60 | 0.00 | 0.60 | 0.60 | 0.00 | 0.60 | 0.60 | 0.00 |
| Female | 0.40 | 0.40 | 0.00 | 0.41 | 0.41 | 0.00 | 0.40 | 0.40 | 0.00 | 0.40 | 0.40 | 0.00 | 0.40 | 0.40 | 0.00 |
| First hospitalization year |  |  |  |  |  |  |  |  |  |  |  |  |  |  |  |
| 2015 | 0.44 | 0.43 | 0.00 | 0.42 | 0.43 | -0.02 | 0.43 | 0.43 | -0.01 | 0.43 | 0.44 | -0.02 | 0.42 | 0.43 | -0.03 |
| 2016 | 0.33 | 0.33 | 0.00 | 0.34 | 0.33 | 0.03 | 0.32 | 0.32 | 0.00 | 0.32 | 0.32 | 0.00 | 0.33 | 0.31 | 0.03 |
| 2017 | 0.23 | 0.23 | 0.00 | 0.24 | 0.25 | -0.02 | 0.25 | 0.24 | 0.01 | 0.25 | 0.24 | 0.02 | 0.25 | 0.25 | 0.00 |
| City |  |  |  |  |  |  |  |  |  |  |  |  |  |  |  |
| Dongying | 0.35 | 0.35 | 0.00 | 0.34 | 0.34 | 0.00 | 0.36 | 0.36 | 0.00 | 0.35 | 0.35 | 0.00 | 0.36 | 0.36 | 0.00 |
| Jinan | 0.01 | 0.01 | 0.00 | 0.01 | 0.01 | 0.00 | 0.02 | 0.02 | 0.00 | 0.02 | 0.02 | 0.00 | 0.02 | 0.02 | 0.00 |
| Qingdao | 0.03 | 0.03 | 0.00 | 0.03 | 0.03 | 0.00 | 0.03 | 0.03 | 0.00 | 0.03 | 0.03 | 0.00 | 0.03 | 0.03 | 0.00 |
| Taian | 0.20 | 0.20 | 0.00 | 0.20 | 0.20 | 0.00 | 0.20 | 0.20 | 0.00 | 0.20 | 0.20 | 0.00 | 0.19 | 0.19 | 0.00 |
| Weifang | 0.10 | 0.10 | 0.00 | 0.11 | 0.11 | 0.00 | 0.11 | 0.11 | 0.00 | 0.11 | 0.11 | 0.00 | 0.11 | 0.11 | 0.00 |
| Yantai | 0.13 | 0.13 | 0.00 | 0.12 | 0.12 | 0.00 | 0.12 | 0.12 | 0.00 | 0.12 | 0.12 | 0.00 | 0.13 | 0.13 | 0.00 |
| Zibo | 0.19 | 0.19 | 0.00 | 0.18 | 0.18 | 0.00 | 0.17 | 0.17 | 0.00 | 0.18 | 0.18 | 0.00 | 0.17 | 0.17 | 0.00 |
| Marital status |  |  |  |  |  |  |  |  |  |  |  |  |  |  |  |
| Married | 0.66 | 0.76 | -0.21 | 0.69 | 0.68 | 0.02 | 0.72 | 0.72 | 0.01 | 0.72 | 0.72 | 0.01 | 0.72 | 0.72 | 0.01 |
| Single | 0.02 | 0.01 | 0.07 | 0.02 | 0.02 | 0.00 | 0.02 | 0.02 | 0.01 | 0.02 | 0.02 | -0.01 | 0.02 | 0.02 | 0.00 |
| Widowed/divorced | 0.02 | 0.06 | -0.26 | 0.02 | 0.02 | 0.00 | 0.02 | 0.02 | 0.01 | 0.02 | 0.02 | 0.03 | 0.02 | 0.03 | -0.03 |
| Unknown | 0.30 | 0.17 | 0.28 | 0.27 | 0.28 | -0.02 | 0.23 | 0.24 | -0.02 | 0.23 | 0.24 | -0.02 | 0.24 | 0.24 | 0.00 |
| Cancer type |  |  |  |  |  |  |  |  |  |  |  |  |  |  |  |
| Bladder | 0.02 | 0.04 | -0.11 | 0.02 | 0.03 | -0.08 | 0.02 | 0.02 | 0.00 | 0.02 | 0.02 | 0.05 | 0.02 | 0.03 | -0.03 |
| Brain | 0.01 | 0.01 | 0.04 | 0.01 | 0.01 | 0.03 | 0.01 | 0.01 | 0.05 | 0.01 | 0.01 | 0.02 | 0.01 | 0.01 | 0.02 |
| Breast | 0.04 | 0.12 | -0.44 | 0.04 | 0.12 | -0.45 | 0.04 | 0.05 | -0.04 | 0.04 | 0.05 | -0.04 | 0.04 | 0.04 | -0.01 |
| Cervix | 0.05 | 0.02 | 0.13 | 0.06 | 0.02 | 0.17 | 0.05 | 0.05 | -0.01 | 0.05 | 0.05 | 0.00 | 0.05 | 0.05 | 0.03 |
| Colorectum | 0.30 | 0.16 | 0.30 | 0.27 | 0.20 | 0.16 | 0.24 | 0.25 | -0.04 | 0.24 | 0.24 | -0.01 | 0.23 | 0.26 | -0.06 |
| Gallbladder | 0.01 | 0.02 | -0.09 | 0.01 | 0.02 | -0.10 | 0.01 | 0.02 | -0.06 | 0.01 | 0.01 | -0.04 | 0.01 | 0.01 | -0.04 |
| Kidney | 0.03 | 0.03 | -0.01 | 0.03 | 0.03 | 0.02 | 0.03 | 0.03 | 0.02 | 0.03 | 0.03 | 0.01 | 0.03 | 0.03 | 0.03 |
| Larynx | 0.00 | 0.00 | -0.01 | 0.00 | 0.00 | -0.02 | 0.00 | 0.00 | 0.05 | 0.00 | 0.00 | 0.05 | 0.00 | 0.00 | 0.03 |
| Leukemia | 0.01 | 0.01 | -0.02 | 0.01 | 0.02 | -0.05 | 0.01 | 0.01 | 0.01 | 0.01 | 0.01 | 0.04 | 0.01 | 0.02 | -0.04 |
| Liver | 0.06 | 0.06 | -0.01 | 0.06 | 0.06 | 0.00 | 0.07 | 0.06 | 0.02 | 0.07 | 0.07 | -0.01 | 0.07 | 0.07 | -0.01 |
| Lung | 0.18 | 0.17 | 0.01 | 0.18 | 0.16 | 0.07 | 0.20 | 0.20 | -0.01 | 0.20 | 0.20 | -0.02 | 0.20 | 0.20 | -0.01 |
| Lymphoma | 0.04 | 0.04 | -0.02 | 0.04 | 0.03 | 0.02 | 0.04 | 0.04 | 0.00 | 0.04 | 0.04 | 0.02 | 0.04 | 0.04 | 0.00 |
| Melanoma | 0.01 | 0.00 | 0.07 | 0.01 | 0.00 | 0.05 | 0.00 | 0.00 | 0.00 | 0.00 | 0.00 | 0.00 | 0.00 | 0.01 | -0.09 |
| Nasopharynx | 0.01 | 0.01 | 0.04 | 0.01 | 0.01 | 0.04 | 0.01 | 0.01 | 0.02 | 0.01 | 0.01 | 0.05 | 0.01 | 0.01 | 0.00 |
| Esophagus | 0.04 | 0.05 | -0.04 | 0.04 | 0.05 | -0.03 | 0.05 | 0.05 | -0.01 | 0.05 | 0.05 | -0.01 | 0.05 | 0.04 | 0.05 |
| Oral | 0.01 | 0.01 | 0.02 | 0.01 | 0.01 | 0.02 | 0.01 | 0.01 | 0.04 | 0.01 | 0.01 | 0.02 | 0.01 | 0.01 | 0.04 |
| Other skin | 0.00 | 0.01 | -0.09 | 0.00 | 0.01 | -0.11 | 0.00 | 0.00 | 0.06 | 0.00 | 0.00 | 0.03 | 0.00 | 0.00 | 0.06 |
| Other thoracic | 0.01 | 0.00 | 0.04 | 0.01 | 0.00 | 0.04 | 0.00 | 0.00 | -0.02 | 0.00 | 0.00 | 0.00 | 0.00 | 0.00 | 0.01 |
| Pancreas | 0.01 | 0.02 | -0.06 | 0.01 | 0.01 | -0.04 | 0.01 | 0.01 | 0.02 | 0.01 | 0.01 | 0.02 | 0.01 | 0.01 | 0.03 |
| Prostate | 0.01 | 0.02 | -0.12 | 0.01 | 0.01 | -0.03 | 0.01 | 0.01 | 0.02 | 0.01 | 0.01 | -0.01 | 0.01 | 0.01 | -0.02 |
| Stomach | 0.09 | 0.11 | -0.06 | 0.10 | 0.10 | 0.00 | 0.10 | 0.10 | 0.02 | 0.10 | 0.10 | 0.00 | 0.10 | 0.09 | 0.05 |
| Thyroid | 0.06 | 0.08 | -0.09 | 0.06 | 0.09 | -0.10 | 0.07 | 0.07 | 0.00 | 0.07 | 0.07 | -0.03 | 0.07 | 0.07 | 0.00 |
| Hypertension |  |  |  |  |  |  |  |  |  |  |  |  |  |  |  |
| No | 0.99 | 0.97 | 0.11 | 0.99 | 0.97 | 0.11 | 0.99 | 0.98 | 0.05 | 0.99 | 0.99 | 0.01 | 0.99 | 0.98 | 0.03 |
| Yes | 0.01 | 0.03 | -0.11 | 0.01 | 0.03 | -0.11 | 0.01 | 0.02 | -0.05 | 0.01 | 0.01 | -0.01 | 0.01 | 0.02 | -0.03 |
| Diabetes |  |  |  |  |  |  |  |  |  |  |  |  |  |  |  |
| No | 0.99 | 0.97 | 0.14 | 0.99 | 0.97 | 0.14 | 0.99 | 0.98 | 0.10 | 0.99 | 0.99 | -0.02 | 0.99 | 0.99 | -0.03 |
| Yes | 0.01 | 0.03 | -0.14 | 0.01 | 0.03 | -0.14 | 0.01 | 0.02 | -0.10 | 0.01 | 0.01 | 0.02 | 0.01 | 0.01 | 0.03 |
| Heart |  |  |  |  |  |  |  |  |  |  |  |  |  |  |  |
| No | 0.95 | 0.92 | 0.15 | 0.95 | 0.93 | 0.10 | 0.96 | 0.94 | 0.07 | 0.95 | 0.96 | -0.06 | 0.95 | 0.96 | -0.03 |
| Yes | 0.05 | 0.08 | -0.15 | 0.05 | 0.07 | -0.10 | 0.04 | 0.06 | -0.07 | 0.05 | 0.04 | 0.06 | 0.05 | 0.04 | 0.03 |
| Osteoporosis |  |  |  |  |  |  |  |  |  |  |  |  |  |  |  |
| No | 0.90 | 0.86 | 0.12 | 0.90 | 0.84 | 0.19 | 0.90 | 0.84 | 0.19 | 0.89 | 0.90 | -0.03 | 0.89 | 0.90 | -0.04 |
| Yes | 0.10 | 0.14 | -0.12 | 0.10 | 0.16 | -0.19 | 0.10 | 0.16 | -0.19 | 0.11 | 0.10 | 0.03 | 0.11 | 0.10 | 0.04 |
| Cerebral |  |  |  |  |  |  |  |  |  |  |  |  |  |  |  |
| No | 0.96 | 0.94 | 0.09 | 0.96 | 0.95 | 0.04 | 0.96 | 0.94 | 0.06 | 0.95 | 0.96 | -0.03 | 0.95 | 0.96 | -0.02 |
| Yes | 0.04 | 0.06 | -0.09 | 0.04 | 0.05 | -0.04 | 0.04 | 0.06 | -0.06 | 0.05 | 0.04 | 0.03 | 0.05 | 0.04 | 0.02 |
| Comorbidity |  |  |  |  |  |  |  |  |  |  |  |  |  |  |  |
| No | 0.80 | 0.72 | 0.20 | 0.81 | 0.72 | 0.21 | 0.81 | 0.73 | 0.19 | 0.80 | 0.82 | -0.07 | 0.79 | 0.82 | -0.06 |
| Yes | 0.20 | 0.28 | -0.20 | 0.19 | 0.28 | -0.21 | 0.19 | 0.27 | -0.19 | 0.20 | 0.18 | 0.07 | 0.21 | 0.18 | 0.06 |
| Hospitalization frequency | 4.55 | 3.55 | 0.24 | 4.54 | 3.54 | 0.24 | 4.41 | 3.55 | 0.21 | 4.41 | 3.51 | 0.22 | 3.98 | 3.93 | 0.01 |
| Variables | National | Intra-city | SMD | National | Intra-city | SMD | National | Intra-city | SMD | National | Intra-city | SMD | National | Intra-city | SMD |
| Age group |  |  |  |  |  |  |  |  |  |  |  |  |  |  |  |
| Less40 | 0.11 | 0.10 | 0.02 | 0.10 | 0.09 | 0.06 | 0.09 | 0.10 | -0.05 | 0.09 | 0.11 | -0.09 | 0.09 | 0.09 | -0.01 |
| 40-50 | 0.24 | 0.24 | 0.01 | 0.24 | 0.24 | 0.00 | 0.22 | 0.19 | 0.07 | 0.23 | 0.19 | 0.08 | 0.22 | 0.22 | 0.02 |
| 50-60 | 0.26 | 0.26 | -0.01 | 0.26 | 0.26 | -0.01 | 0.26 | 0.24 | 0.05 | 0.27 | 0.26 | 0.01 | 0.26 | 0.27 | -0.01 |
| Greater60 | 0.39 | 0.40 | -0.01 | 0.40 | 0.41 | -0.02 | 0.42 | 0.46 | -0.07 | 0.42 | 0.44 | -0.03 | 0.43 | 0.43 | 0.00 |
| Sex |  |  |  |  |  |  |  |  |  |  |  |  |  |  |  |
| Male | 0.58 | 0.58 | 0.01 | 0.58 | 0.58 | 0.00 | 0.57 | 0.57 | 0.00 | 0.57 | 0.57 | 0.01 | 0.56 | 0.57 | -0.02 |
| Female | 0.42 | 0.42 | -0.01 | 0.42 | 0.42 | 0.00 | 0.43 | 0.43 | 0.00 | 0.43 | 0.43 | -0.01 | 0.44 | 0.43 | 0.02 |
| First hospitalization year |  |  |  |  |  |  |  |  |  |  |  |  |  |  |  |
| 2015 | 0.36 | 0.36 | -0.01 | 0.36 | 0.35 | 0.02 | 0.36 | 0.36 | -0.01 | 0.37 | 0.33 | 0.07 | 0.37 | 0.35 | 0.05 |
| 2016 | 0.40 | 0.39 | 0.01 | 0.40 | 0.39 | 0.01 | 0.39 | 0.38 | 0.01 | 0.38 | 0.40 | -0.03 | 0.38 | 0.38 | -0.02 |
| 2017 | 0.24 | 0.25 | -0.01 | 0.24 | 0.26 | -0.04 | 0.25 | 0.25 | 0.00 | 0.25 | 0.27 | -0.04 | 0.26 | 0.27 | -0.03 |
| City |  |  |  |  |  |  |  |  |  |  |  |  |  |  |  |
| Dongying | 0.39 | 0.38 | 0.02 | 0.39 | 0.38 | 0.03 | 0.38 | 0.36 | 0.03 | 0.37 | 0.36 | 0.03 | 0.37 | 0.36 | 0.02 |
| Jinan | 0.00 | 0.00 | 0.00 | 0.00 | 0.00 | 0.00 | 0.00 | 0.00 | 0.00 | 0.00 | 0.00 | 0.00 | 0.00 | 0.00 | 0.00 |
| Qingdao | 0.01 | 0.01 | 0.00 | 0.01 | 0.01 | 0.00 | 0.01 | 0.01 | 0.00 | 0.01 | 0.01 | 0.00 | 0.01 | 0.01 | 0.00 |
| Taian | 0.07 | 0.07 | 0.00 | 0.07 | 0.07 | 0.00 | 0.07 | 0.07 | 0.00 | 0.07 | 0.07 | 0.00 | 0.07 | 0.07 | -0.01 |
| Weifang | 0.28 | 0.29 | -0.01 | 0.28 | 0.29 | -0.02 | 0.30 | 0.31 | -0.03 | 0.30 | 0.31 | -0.03 | 0.30 | 0.31 | -0.01 |
| Yantai | 0.02 | 0.02 | 0.00 | 0.02 | 0.03 | 0.00 | 0.03 | 0.03 | -0.01 | 0.03 | 0.03 | -0.01 | 0.03 | 0.03 | -0.01 |
| Zibo | 0.22 | 0.22 | -0.01 | 0.21 | 0.22 | -0.01 | 0.22 | 0.22 | 0.00 | 0.22 | 0.22 | 0.00 | 0.22 | 0.22 | 0.00 |
| Marital status |  |  |  |  |  |  |  |  |  |  |  |  |  |  |  |
| Married | 0.77 | 0.71 | 0.12 | 0.77 | 0.78 | -0.03 | 0.78 | 0.78 | -0.01 | 0.78 | 0.79 | -0.03 | 0.78 | 0.80 | -0.05 |
| Single | 0.05 | 0.02 | 0.11 | 0.04 | 0.03 | 0.06 | 0.02 | 0.02 | -0.01 | 0.02 | 0.02 | -0.01 | 0.02 | 0.02 | -0.03 |
| Widowed/divorced | 0.01 | 0.02 | -0.22 | 0.01 | 0.01 | 0.00 | 0.00 | 0.00 | 0.00 | 0.01 | 0.00 | 0.04 | 0.01 | 0.01 | 0.02 |
| Unknown | 0.18 | 0.24 | -0.15 | 0.18 | 0.18 | 0.01 | 0.20 | 0.19 | 0.01 | 0.20 | 0.19 | 0.02 | 0.20 | 0.17 | 0.06 |
| Cancer type |  |  |  |  |  |  |  |  |  |  |  |  |  |  |  |
| Bladder | 0.02 | 0.03 | -0.15 | 0.02 | 0.03 | -0.13 | 0.02 | 0.02 | -0.02 | 0.02 | 0.02 | 0.01 | 0.02 | 0.02 | 0.01 |
| Brain | 0.02 | 0.01 | 0.06 | 0.02 | 0.01 | 0.03 | 0.01 | 0.00 | 0.09 | 0.01 | 0.01 | 0.06 | 0.01 | 0.01 | 0.01 |
| Breast | 0.09 | 0.15 | -0.19 | 0.09 | 0.15 | -0.20 | 0.10 | 0.11 | -0.04 | 0.10 | 0.13 | -0.08 | 0.10 | 0.13 | -0.08 |
| Cervix | 0.03 | 0.03 | -0.03 | 0.03 | 0.03 | -0.03 | 0.03 | 0.02 | 0.05 | 0.03 | 0.01 | 0.09 | 0.03 | 0.03 | 0.02 |
| Colorectum | 0.13 | 0.14 | -0.02 | 0.13 | 0.12 | 0.03 | 0.14 | 0.14 | 0.01 | 0.14 | 0.13 | 0.04 | 0.14 | 0.13 | 0.02 |
| Gallbladder | 0.03 | 0.01 | 0.08 | 0.03 | 0.01 | 0.08 | 0.03 | 0.02 | 0.04 | 0.03 | 0.02 | 0.06 | 0.03 | 0.04 | -0.06 |
| Kidney | 0.03 | 0.03 | 0.00 | 0.03 | 0.03 | -0.02 | 0.03 | 0.02 | 0.03 | 0.03 | 0.03 | -0.03 | 0.03 | 0.02 | 0.03 |
| Leukemia | 0.06 | 0.02 | 0.18 | 0.06 | 0.02 | 0.17 | 0.02 | 0.02 | 0.02 | 0.02 | 0.02 | 0.03 | 0.02 | 0.02 | 0.03 |
| Liver | 0.10 | 0.07 | 0.09 | 0.10 | 0.07 | 0.10 | 0.10 | 0.13 | -0.09 | 0.10 | 0.11 | -0.04 | 0.09 | 0.10 | -0.04 |
| Lung | 0.15 | 0.15 | 0.01 | 0.15 | 0.16 | -0.04 | 0.15 | 0.16 | -0.01 | 0.16 | 0.17 | -0.02 | 0.16 | 0.17 | -0.04 |
| Lymphoma | 0.05 | 0.05 | 0.02 | 0.05 | 0.05 | 0.01 | 0.06 | 0.06 | -0.03 | 0.05 | 0.04 | 0.07 | 0.06 | 0.05 | 0.03 |
| Nasopharynx | 0.01 | 0.01 | 0.03 | 0.01 | 0.00 | 0.05 | 0.01 | 0.00 | 0.07 | 0.01 | 0.01 | 0.05 | 0.01 | 0.01 | 0.01 |
| Esophagus | 0.03 | 0.04 | -0.05 | 0.03 | 0.04 | -0.06 | 0.04 | 0.03 | 0.05 | 0.04 | 0.03 | 0.03 | 0.04 | 0.02 | 0.09 |
| Oral | 0.02 | 0.01 | 0.05 | 0.02 | 0.02 | 0.04 | 0.01 | 0.01 | 0.00 | 0.02 | 0.02 | -0.02 | 0.02 | 0.02 | 0.01 |
| Pancreas | 0.02 | 0.01 | 0.05 | 0.02 | 0.01 | 0.05 | 0.02 | 0.02 | 0.03 | 0.02 | 0.02 | 0.02 | 0.01 | 0.02 | -0.02 |
| Prostate | 0.03 | 0.02 | 0.07 | 0.03 | 0.01 | 0.11 | 0.03 | 0.03 | -0.01 | 0.03 | 0.04 | -0.05 | 0.03 | 0.02 | 0.05 |
| Stomach | 0.10 | 0.10 | -0.01 | 0.10 | 0.11 | -0.03 | 0.10 | 0.10 | 0.02 | 0.10 | 0.11 | -0.02 | 0.11 | 0.10 | 0.04 |
| Thyroid | 0.09 | 0.11 | -0.08 | 0.09 | 0.11 | -0.06 | 0.10 | 0.11 | -0.02 | 0.09 | 0.11 | -0.05 | 0.10 | 0.10 | -0.03 |
| Hypertension |  |  |  |  |  |  |  |  |  |  |  |  |  |  |  |
| No | 0.98 | 0.97 | 0.10 | 0.98 | 0.97 | 0.12 | 0.98 | 0.98 | 0.06 | 0.98 | 0.99 | -0.04 | 0.98 | 0.98 | -0.01 |
| Yes | 0.02 | 0.03 | -0.10 | 0.02 | 0.03 | -0.12 | 0.02 | 0.02 | -0.06 | 0.02 | 0.01 | 0.04 | 0.02 | 0.02 | 0.01 |
| Diabetes |  |  |  |  |  |  |  |  |  |  |  |  |  |  |  |
| No | 0.97 | 0.98 | -0.06 | 0.97 | 0.97 | -0.04 | 0.97 | 0.99 | -0.11 | 0.97 | 0.98 | -0.07 | 0.97 | 0.98 | -0.07 |
| Yes | 0.03 | 0.02 | 0.06 | 0.03 | 0.03 | 0.04 | 0.03 | 0.01 | 0.11 | 0.03 | 0.02 | 0.07 | 0.03 | 0.02 | 0.07 |
| Heart |  |  |  |  |  |  |  |  |  |  |  |  |  |  |  |
| No | 0.95 | 0.93 | 0.13 | 0.95 | 0.93 | 0.11 | 0.95 | 0.94 | 0.03 | 0.96 | 0.96 | 0.01 | 0.95 | 0.96 | -0.02 |
| Yes | 0.05 | 0.07 | -0.13 | 0.05 | 0.07 | -0.11 | 0.05 | 0.06 | -0.03 | 0.04 | 0.04 | -0.01 | 0.05 | 0.04 | 0.02 |
| Osteoporosis |  |  |  |  |  |  |  |  |  |  |  |  |  |  |  |
| No | 0.84 | 0.84 | 0.01 | 0.84 | 0.83 | 0.03 | 0.84 | 0.85 | -0.03 | 0.84 | 0.85 | -0.02 | 0.84 | 0.86 | -0.05 |
| Yes | 0.16 | 0.16 | -0.01 | 0.16 | 0.17 | -0.03 | 0.16 | 0.15 | 0.03 | 0.16 | 0.15 | 0.02 | 0.16 | 0.14 | 0.05 |
| Cerebral |  |  |  |  |  |  |  |  |  |  |  |  |  |  |  |
| No | 0.97 | 0.95 | 0.11 | 0.97 | 0.95 | 0.11 | 0.97 | 0.96 | 0.08 | 0.97 | 0.97 | 0.00 | 0.97 | 0.98 | -0.10 |
| Yes | 0.03 | 0.05 | -0.11 | 0.03 | 0.05 | -0.11 | 0.03 | 0.04 | -0.08 | 0.03 | 0.03 | 0.00 | 0.03 | 0.02 | 0.10 |
| Comorbidity |  |  |  |  |  |  |  |  |  |  |  |  |  |  |  |
| No | 0.75 | 0.72 | 0.07 | 0.75 | 0.71 | 0.10 | 0.75 | 0.75 | 0.00 | 0.75 | 0.78 | -0.06 | 0.75 | 0.79 | -0.10 |
| Yes | 0.25 | 0.28 | -0.07 | 0.25 | 0.29 | -0.10 | 0.25 | 0.25 | 0.00 | 0.25 | 0.22 | 0.06 | 0.25 | 0.21 | 0.10 |
| Hospitalization frequency | 4.09 | 3.47 | 0.15 | 4.09 | 3.58 | 0.13 | 3.90 | 3.43 | 0.12 | 4.04 | 3.62 | 0.10 | 3.94 | 3.51 | 0.11 |

Local, local center pattern; National, National center pattern; SMD, Standardized mean difference.

# Supplementary Table 8. Matching results for cancer patients in cities with healthcare resources less than average level

|  | Demographic matched | | | Marriage matched | | | Cancer matched | | | Morbidity matched | | | Frequency matched | | |
| --- | --- | --- | --- | --- | --- | --- | --- | --- | --- | --- | --- | --- | --- | --- | --- |
| Variables | Local | Intra-city | SMD | Local | Intra-city | SMD | Local | Intra-city | SMD | Local | Intra-city | SMD | Local | Intra-city | SMD |
| Age group |  |  |  |  |  |  |  |  |  |  |  |  |  |  |  |
| Less40 | 0.12 | 0.10 | 0.06 | 0.12 | 0.11 | 0.02 | 0.12 | 0.10 | 0.07 | 0.12 | 0.09 | 0.09 | 0.10 | 0.10 | 0.01 |
| 40-50 | 0.14 | 0.14 | -0.01 | 0.14 | 0.15 | -0.01 | 0.16 | 0.13 | 0.08 | 0.15 | 0.14 | 0.04 | 0.14 | 0.13 | 0.04 |
| 50-60 | 0.23 | 0.25 | -0.04 | 0.27 | 0.24 | 0.07 | 0.22 | 0.21 | 0.01 | 0.23 | 0.23 | 0.00 | 0.24 | 0.24 | 0.00 |
| Greater60 | 0.51 | 0.51 | 0.00 | 0.47 | 0.50 | -0.06 | 0.50 | 0.56 | -0.11 | 0.51 | 0.55 | -0.08 | 0.52 | 0.53 | -0.03 |
| Sex |  |  |  |  |  |  |  |  |  |  |  |  |  |  |  |
| Male | 0.55 | 0.55 | 0.00 | 0.55 | 0.55 | 0.00 | 0.56 | 0.56 | 0.00 | 0.56 | 0.56 | 0.00 | 0.57 | 0.57 | 0.00 |
| Female | 0.45 | 0.45 | 0.00 | 0.45 | 0.45 | 0.00 | 0.44 | 0.44 | 0.00 | 0.44 | 0.44 | 0.00 | 0.43 | 0.43 | 0.00 |
| First hospitalization year |  |  |  |  |  |  |  |  |  |  |  |  |  |  |  |
| 2015 | 0.45 | 0.39 | 0.14 | 0.39 | 0.36 | 0.05 | 0.31 | 0.37 | -0.12 | 0.33 | 0.36 | -0.08 | 0.37 | 0.36 | 0.01 |
| 2016 | 0.30 | 0.36 | -0.14 | 0.31 | 0.38 | -0.15 | 0.39 | 0.35 | 0.09 | 0.38 | 0.36 | 0.05 | 0.36 | 0.36 | 0.00 |
| 2017 | 0.25 | 0.25 | -0.01 | 0.30 | 0.26 | 0.09 | 0.30 | 0.29 | 0.02 | 0.29 | 0.28 | 0.03 | 0.27 | 0.27 | -0.01 |
| City |  |  |  |  |  |  |  |  |  |  |  |  |  |  |  |
| Binzhou | 0.10 | 0.10 | 0.00 | 0.10 | 0.10 | 0.00 | 0.10 | 0.10 | 0.00 | 0.10 | 0.10 | 0.00 | 0.10 | 0.10 | 0.00 |
| Dezhou | 0.32 | 0.32 | 0.00 | 0.30 | 0.30 | 0.00 | 0.29 | 0.29 | 0.00 | 0.30 | 0.30 | 0.00 | 0.30 | 0.30 | 0.00 |
| Heze | 0.13 | 0.13 | 0.00 | 0.13 | 0.13 | 0.00 | 0.14 | 0.14 | 0.00 | 0.14 | 0.14 | 0.00 | 0.14 | 0.14 | 0.00 |
| Jining | 0.27 | 0.27 | 0.00 | 0.28 | 0.28 | 0.00 | 0.28 | 0.28 | 0.00 | 0.27 | 0.27 | 0.00 | 0.29 | 0.29 | 0.00 |
| Liaocheng | 0.02 | 0.02 | 0.00 | 0.03 | 0.03 | 0.00 | 0.03 | 0.03 | 0.00 | 0.03 | 0.03 | 0.00 | 0.03 | 0.03 | 0.00 |
| Linyi | 0.07 | 0.07 | 0.00 | 0.08 | 0.08 | 0.00 | 0.08 | 0.08 | 0.00 | 0.08 | 0.08 | 0.00 | 0.08 | 0.08 | 0.00 |
| Weifang | 0.02 | 0.02 | 0.00 | 0.02 | 0.02 | 0.00 | 0.02 | 0.02 | 0.00 | 0.02 | 0.02 | 0.00 | 0.02 | 0.02 | 0.00 |
| Zaozhuang | 0.06 | 0.06 | 0.00 | 0.06 | 0.06 | 0.00 | 0.06 | 0.06 | 0.00 | 0.06 | 0.06 | 0.00 | 0.05 | 0.05 | 0.00 |
| Marital status |  |  |  |  |  |  |  |  |  |  |  |  |  |  |  |
| Married | 0.45 | 0.58 | -0.27 | 0.48 | 0.50 | -0.04 | 0.54 | 0.52 | 0.04 | 0.53 | 0.53 | 0.00 | 0.52 | 0.51 | 0.01 |
| Single | 0.02 | 0.02 | 0.01 | 0.02 | 0.02 | -0.01 | 0.02 | 0.02 | 0.04 | 0.02 | 0.02 | 0.01 | 0.02 | 0.02 | -0.01 |
| Widowed/divorced | 0.02 | 0.04 | -0.14 | 0.02 | 0.02 | 0.01 | 0.02 | 0.02 | -0.02 | 0.02 | 0.02 | 0.00 | 0.02 | 0.02 | -0.01 |
| Unknown | 0.51 | 0.36 | 0.30 | 0.48 | 0.46 | 0.04 | 0.42 | 0.44 | -0.05 | 0.43 | 0.43 | 0.00 | 0.44 | 0.44 | -0.01 |
| Cancer type |  |  |  |  |  |  |  |  |  |  |  |  |  |  |  |
| Bladder | 0.01 | 0.03 | -0.23 | 0.01 | 0.02 | -0.12 | 0.01 | 0.01 | 0.01 | 0.01 | 0.01 | -0.02 | 0.01 | 0.01 | -0.02 |
| Brain | 0.01 | 0.02 | -0.22 | 0.01 | 0.02 | -0.14 | 0.01 | 0.01 | 0.01 | 0.01 | 0.01 | 0.03 | 0.01 | 0.01 | 0.00 |
| Breast | 0.02 | 0.11 | -0.71 | 0.01 | 0.11 | -0.79 | 0.01 | 0.01 | -0.03 | 0.01 | 0.01 | -0.04 | 0.01 | 0.02 | -0.04 |
| Cervix | 0.05 | 0.06 | -0.02 | 0.06 | 0.06 | -0.01 | 0.07 | 0.08 | -0.03 | 0.06 | 0.07 | -0.03 | 0.06 | 0.06 | 0.01 |
| Colorectum | 0.35 | 0.13 | 0.46 | 0.32 | 0.14 | 0.38 | 0.24 | 0.25 | -0.04 | 0.25 | 0.26 | -0.03 | 0.27 | 0.25 | 0.04 |
| Gallbladder | 0.01 | 0.00 | 0.05 | 0.01 | 0.01 | 0.05 | 0.01 | 0.01 | 0.00 | 0.01 | 0.01 | 0.02 | 0.01 | 0.01 | -0.02 |
| Kidney | 0.03 | 0.02 | 0.03 | 0.03 | 0.03 | 0.03 | 0.03 | 0.03 | 0.04 | 0.03 | 0.02 | 0.06 | 0.03 | 0.03 | -0.01 |
| Larynx | 0.00 | 0.01 | -0.07 | 0.00 | 0.01 | -0.08 | 0.01 | 0.01 | 0.00 | 0.01 | 0.01 | 0.00 | 0.01 | 0.00 | 0.04 |
| Leukemia | 0.02 | 0.03 | -0.13 | 0.02 | 0.03 | -0.11 | 0.02 | 0.03 | -0.02 | 0.02 | 0.03 | -0.03 | 0.02 | 0.03 | -0.06 |
| Liver | 0.06 | 0.07 | -0.06 | 0.06 | 0.07 | -0.03 | 0.08 | 0.08 | 0.00 | 0.07 | 0.07 | -0.03 | 0.07 | 0.07 | 0.01 |
| Lung | 0.15 | 0.17 | -0.06 | 0.15 | 0.16 | -0.04 | 0.15 | 0.16 | -0.02 | 0.17 | 0.16 | 0.02 | 0.17 | 0.18 | -0.04 |
| Lymphoma | 0.04 | 0.04 | -0.03 | 0.04 | 0.04 | -0.02 | 0.05 | 0.04 | 0.04 | 0.05 | 0.05 | 0.00 | 0.04 | 0.05 | -0.03 |
| Melanoma | 0.00 | 0.00 | 0.04 | 0.01 | 0.00 | 0.04 | 0.00 | 0.00 | 0.03 | 0.00 | 0.00 | 0.00 | 0.00 | 0.00 | 0.01 |
| Nasopharynx | 0.01 | 0.00 | 0.05 | 0.01 | 0.00 | 0.04 | 0.01 | 0.01 | 0.03 | 0.01 | 0.00 | 0.03 | 0.01 | 0.01 | 0.01 |
| Esophagus | 0.06 | 0.07 | -0.06 | 0.06 | 0.08 | -0.06 | 0.08 | 0.08 | 0.01 | 0.07 | 0.08 | -0.04 | 0.08 | 0.08 | 0.02 |
| Oral | 0.01 | 0.01 | 0.00 | 0.01 | 0.01 | 0.00 | 0.02 | 0.02 | -0.04 | 0.02 | 0.02 | 0.01 | 0.02 | 0.02 | -0.01 |
| Other skin | 0.00 | 0.01 | -0.03 | 0.00 | 0.01 | -0.01 | 0.00 | 0.00 | 0.01 | 0.00 | 0.00 | 0.00 | 0.00 | 0.00 | 0.00 |
| Other thoracic | 0.00 | 0.00 | 0.06 | 0.00 | 0.00 | 0.04 | 0.00 | 0.00 | -0.02 | 0.00 | 0.00 | 0.01 | 0.00 | 0.00 | 0.01 |
| Pancreas | 0.00 | 0.01 | -0.17 | 0.01 | 0.02 | -0.14 | 0.01 | 0.01 | 0.00 | 0.01 | 0.01 | 0.01 | 0.00 | 0.01 | -0.01 |
| Prostate | 0.01 | 0.02 | -0.05 | 0.01 | 0.02 | -0.03 | 0.01 | 0.02 | -0.05 | 0.01 | 0.01 | -0.01 | 0.01 | 0.01 | 0.01 |
| Stomach | 0.07 | 0.08 | -0.04 | 0.07 | 0.09 | -0.05 | 0.09 | 0.08 | 0.01 | 0.09 | 0.09 | 0.00 | 0.09 | 0.08 | 0.03 |
| Thyroid | 0.08 | 0.07 | 0.03 | 0.09 | 0.07 | 0.05 | 0.10 | 0.08 | 0.07 | 0.09 | 0.07 | 0.07 | 0.08 | 0.08 | -0.01 |
| Hypertension |  |  |  |  |  |  |  |  |  |  |  |  |  |  |  |
| No | 0.98 | 0.97 | 0.04 | 0.98 | 0.98 | 0.01 | 0.98 | 0.98 | -0.02 | 0.97 | 0.98 | -0.04 | 0.98 | 0.98 | -0.03 |
| Yes | 0.02 | 0.03 | -0.04 | 0.02 | 0.02 | -0.01 | 0.02 | 0.02 | 0.02 | 0.03 | 0.02 | 0.04 | 0.02 | 0.02 | 0.03 |
| Diabetes |  |  |  |  |  |  |  |  |  |  |  |  |  |  |  |
| No | 0.99 | 0.98 | 0.08 | 0.99 | 0.98 | 0.05 | 0.99 | 0.98 | 0.04 | 0.98 | 0.99 | -0.06 | 0.99 | 0.98 | 0.04 |
| Yes | 0.01 | 0.02 | -0.08 | 0.01 | 0.02 | -0.05 | 0.01 | 0.02 | -0.04 | 0.02 | 0.01 | 0.06 | 0.01 | 0.02 | -0.04 |
| Heart |  |  |  |  |  |  |  |  |  |  |  |  |  |  |  |
| No | 0.96 | 0.94 | 0.12 | 0.96 | 0.94 | 0.13 | 0.96 | 0.94 | 0.08 | 0.95 | 0.97 | -0.05 | 0.96 | 0.97 | -0.03 |
| Yes | 0.04 | 0.06 | -0.12 | 0.04 | 0.06 | -0.13 | 0.04 | 0.06 | -0.08 | 0.05 | 0.03 | 0.05 | 0.04 | 0.03 | 0.03 |
| Osteoporosis |  |  |  |  |  |  |  |  |  |  |  |  |  |  |  |
| No | 0.70 | 0.70 | -0.02 | 0.71 | 0.70 | 0.01 | 0.73 | 0.71 | 0.05 | 0.72 | 0.72 | 0.00 | 0.71 | 0.72 | -0.03 |
| Yes | 0.30 | 0.30 | 0.02 | 0.29 | 0.30 | -0.01 | 0.27 | 0.29 | -0.05 | 0.28 | 0.28 | 0.00 | 0.29 | 0.28 | 0.03 |
| Cerebral |  |  |  |  |  |  |  |  |  |  |  |  |  |  |  |
| No | 0.95 | 0.95 | 0.02 | 0.96 | 0.95 | 0.02 | 0.95 | 0.94 | 0.05 | 0.95 | 0.96 | -0.04 | 0.95 | 0.96 | -0.03 |
| Yes | 0.05 | 0.05 | -0.02 | 0.04 | 0.05 | -0.02 | 0.05 | 0.06 | -0.05 | 0.05 | 0.04 | 0.04 | 0.05 | 0.04 | 0.03 |
| Comorbidity |  |  |  |  |  |  |  |  |  |  |  |  |  |  |  |
| No | 0.62 | 0.60 | 0.05 | 0.64 | 0.61 | 0.06 | 0.65 | 0.61 | 0.08 | 0.64 | 0.65 | -0.03 | 0.63 | 0.65 | -0.04 |
| Yes | 0.38 | 0.40 | -0.05 | 0.36 | 0.39 | -0.06 | 0.35 | 0.39 | -0.08 | 0.36 | 0.35 | 0.03 | 0.37 | 0.35 | 0.04 |
| Hospitalization frequency | 3.84 | 2.61 | 0.34 | 3.71 | 2.56 | 0.33 | 3.76 | 2.43 | 0.38 | 3.78 | 2.53 | 0.36 | 3.23 | 3.17 | 0.02 |
| Variables | National | Intra-city | SMD | National | Intra-city | SMD | National | Intra-city | SMD | National | Intra-city | SMD | National | Intra-city | SMD |
| Age group |  |  |  |  |  |  |  |  |  |  |  |  |  |  |  |
| Less40 | 0.13 | 0.13 | 0.00 | 0.12 | 0.11 | 0.03 | 0.11 | 0.11 | 0.00 | 0.14 | 0.11 | 0.07 | 0.14 | 0.10 | 0.12 |
| 40-50 | 0.23 | 0.23 | 0.01 | 0.24 | 0.23 | 0.02 | 0.22 | 0.18 | 0.10 | 0.21 | 0.14 | 0.17 | 0.21 | 0.18 | 0.08 |
| 50-60 | 0.25 | 0.24 | 0.01 | 0.25 | 0.25 | 0.00 | 0.25 | 0.25 | -0.01 | 0.24 | 0.23 | 0.02 | 0.23 | 0.23 | 0.01 |
| Greater60 | 0.39 | 0.39 | -0.01 | 0.40 | 0.41 | -0.03 | 0.42 | 0.46 | -0.07 | 0.42 | 0.52 | -0.21 | 0.42 | 0.50 | -0.16 |
| Sex |  |  |  |  |  |  |  |  |  |  |  |  |  |  |  |
| Male | 0.59 | 0.60 | 0.00 | 0.59 | 0.59 | 0.00 | 0.60 | 0.62 | -0.03 | 0.61 | 0.61 | -0.01 | 0.59 | 0.61 | -0.04 |
| Female | 0.41 | 0.40 | 0.00 | 0.41 | 0.41 | 0.00 | 0.40 | 0.38 | 0.03 | 0.39 | 0.39 | 0.01 | 0.41 | 0.39 | 0.04 |
| First hospitalization year |  |  |  |  |  |  |  |  |  |  |  |  |  |  |  |
| 2015 | 0.39 | 0.39 | 0.00 | 0.38 | 0.38 | 0.00 | 0.37 | 0.37 | 0.01 | 0.36 | 0.39 | -0.05 | 0.36 | 0.32 | 0.09 |
| 2016 | 0.40 | 0.39 | 0.00 | 0.40 | 0.40 | 0.00 | 0.41 | 0.42 | -0.04 | 0.40 | 0.39 | 0.02 | 0.40 | 0.43 | -0.06 |
| 2017 | 0.22 | 0.22 | 0.00 | 0.22 | 0.22 | 0.00 | 0.22 | 0.21 | 0.04 | 0.23 | 0.22 | 0.03 | 0.24 | 0.25 | -0.03 |
| City |  |  |  |  |  |  |  |  |  |  |  |  |  |  |  |
| Binzhou | 0.05 | 0.05 | 0.00 | 0.05 | 0.05 | -0.01 | 0.05 | 0.05 | -0.02 | 0.05 | 0.05 | -0.02 | 0.05 | 0.05 | -0.02 |
| Dezhou | 0.27 | 0.27 | -0.01 | 0.27 | 0.27 | 0.00 | 0.25 | 0.23 | 0.05 | 0.24 | 0.23 | 0.02 | 0.27 | 0.24 | 0.06 |
| Heze | 0.13 | 0.12 | 0.03 | 0.12 | 0.11 | 0.03 | 0.12 | 0.11 | 0.03 | 0.12 | 0.11 | 0.04 | 0.08 | 0.08 | 0.02 |
| Jining | 0.33 | 0.33 | -0.01 | 0.33 | 0.34 | -0.01 | 0.35 | 0.36 | -0.03 | 0.35 | 0.36 | -0.02 | 0.36 | 0.38 | -0.04 |
| Linyi | 0.19 | 0.19 | -0.01 | 0.19 | 0.19 | -0.01 | 0.20 | 0.21 | -0.03 | 0.21 | 0.22 | -0.02 | 0.20 | 0.21 | -0.03 |
| Weifang | 0.04 | 0.03 | 0.01 | 0.04 | 0.03 | 0.02 | 0.03 | 0.03 | -0.01 | 0.03 | 0.03 | 0.00 | 0.04 | 0.04 | 0.00 |
| Marital status |  |  |  |  |  |  |  |  |  |  |  |  |  |  |  |
| Married | 0.45 | 0.56 | -0.23 | 0.46 | 0.46 | -0.01 | 0.48 | 0.49 | 0.00 | 0.48 | 0.48 | -0.01 | 0.47 | 0.47 | 0.00 |
| Single | 0.03 | 0.02 | 0.04 | 0.02 | 0.02 | 0.02 | 0.03 | 0.01 | 0.09 | 0.03 | 0.02 | 0.03 | 0.02 | 0.03 | -0.03 |
| Widowed/divorced | 0.00 | 0.03 | -0.38 | 0.00 | 0.00 | 0.00 | 0.00 | 0.00 | 0.03 | 0.00 | 0.00 | 0.03 | 0.00 | 0.00 | 0.03 |
| Unknown | 0.51 | 0.38 | 0.26 | 0.52 | 0.52 | 0.01 | 0.48 | 0.50 | -0.03 | 0.49 | 0.50 | 0.00 | 0.51 | 0.50 | 0.00 |
| Cancer type |  |  |  |  |  |  |  |  |  |  |  |  |  |  |  |
| Bladder | 0.01 | 0.02 | -0.09 | 0.01 | 0.02 | -0.10 | 0.01 | 0.01 | -0.01 | 0.01 | 0.01 | -0.01 | 0.01 | 0.01 | 0.01 |
| Brain | 0.06 | 0.05 | 0.04 | 0.06 | 0.05 | 0.01 | 0.06 | 0.04 | 0.07 | 0.06 | 0.05 | 0.05 | 0.06 | 0.05 | 0.04 |
| Breast | 0.04 | 0.12 | -0.38 | 0.04 | 0.10 | -0.27 | 0.05 | 0.05 | -0.02 | 0.05 | 0.04 | 0.03 | 0.05 | 0.05 | 0.01 |
| Cervix | 0.01 | 0.04 | -0.36 | 0.01 | 0.04 | -0.33 | 0.01 | 0.02 | -0.08 | 0.01 | 0.01 | -0.03 | 0.00 | 0.00 | 0.00 |
| Colorectum | 0.07 | 0.13 | -0.22 | 0.07 | 0.16 | -0.32 | 0.08 | 0.10 | -0.08 | 0.08 | 0.08 | -0.01 | 0.08 | 0.08 | -0.02 |
| Gallbladder | 0.01 | 0.01 | 0.02 | 0.01 | 0.01 | 0.02 | 0.01 | 0.01 | -0.01 | 0.01 | 0.01 | 0.02 | 0.01 | 0.01 | 0.02 |
| Kidney | 0.02 | 0.03 | -0.07 | 0.02 | 0.03 | -0.05 | 0.03 | 0.02 | 0.02 | 0.03 | 0.01 | 0.11 | 0.03 | 0.02 | 0.03 |
| Leukemia | 0.07 | 0.06 | 0.04 | 0.06 | 0.04 | 0.06 | 0.05 | 0.05 | 0.00 | 0.06 | 0.06 | 0.01 | 0.04 | 0.05 | -0.05 |
| Liver | 0.10 | 0.04 | 0.19 | 0.11 | 0.04 | 0.23 | 0.10 | 0.10 | 0.00 | 0.11 | 0.12 | -0.04 | 0.11 | 0.09 | 0.06 |
| Lung | 0.17 | 0.18 | -0.02 | 0.17 | 0.16 | 0.03 | 0.19 | 0.19 | 0.00 | 0.19 | 0.18 | 0.03 | 0.19 | 0.19 | 0.00 |
| Lymphoma | 0.09 | 0.04 | 0.16 | 0.08 | 0.04 | 0.18 | 0.09 | 0.06 | 0.09 | 0.08 | 0.07 | 0.02 | 0.06 | 0.07 | -0.03 |
| Nasopharynx | 0.01 | 0.00 | 0.07 | 0.01 | 0.00 | 0.07 | 0.00 | 0.00 | -0.01 | 0.00 | 0.00 | 0.00 | 0.00 | 0.01 | -0.04 |
| Esophagus | 0.04 | 0.05 | -0.03 | 0.04 | 0.06 | -0.06 | 0.05 | 0.04 | 0.02 | 0.05 | 0.05 | 0.00 | 0.05 | 0.04 | 0.03 |
| Oral | 0.01 | 0.02 | -0.18 | 0.01 | 0.02 | -0.17 | 0.01 | 0.01 | 0.02 | 0.01 | 0.01 | -0.01 | 0.01 | 0.00 | 0.04 |
| Pancreas | 0.01 | 0.02 | -0.06 | 0.01 | 0.02 | -0.04 | 0.01 | 0.01 | 0.03 | 0.01 | 0.02 | -0.07 | 0.01 | 0.02 | -0.03 |
| Prostate | 0.03 | 0.01 | 0.10 | 0.03 | 0.02 | 0.06 | 0.03 | 0.03 | -0.01 | 0.02 | 0.04 | -0.17 | 0.02 | 0.03 | -0.10 |
| Stomach | 0.06 | 0.10 | -0.18 | 0.06 | 0.10 | -0.18 | 0.07 | 0.08 | -0.04 | 0.07 | 0.08 | -0.04 | 0.07 | 0.10 | -0.11 |
| Thyroid | 0.19 | 0.08 | 0.29 | 0.19 | 0.10 | 0.23 | 0.16 | 0.17 | -0.02 | 0.15 | 0.14 | 0.01 | 0.18 | 0.17 | 0.05 |
| Hypertension |  |  |  |  |  |  |  |  |  |  |  |  |  |  |  |
| No | 0.99 | 0.97 | 0.21 | 0.99 | 0.98 | 0.12 | 0.99 | 0.98 | 0.07 | 0.99 | 0.99 | -0.01 | 0.99 | 0.99 | -0.01 |
| Yes | 0.01 | 0.03 | -0.21 | 0.01 | 0.03 | -0.12 | 0.01 | 0.02 | -0.07 | 0.01 | 0.01 | 0.01 | 0.01 | 0.01 | 0.01 |
| Diabetes |  |  |  |  |  |  |  |  |  |  |  |  |  |  |  |
| No | 0.98 | 0.98 | -0.01 | 0.98 | 0.99 | -0.04 | 0.98 | 0.98 | 0.03 | 0.99 | 0.98 | 0.05 | 0.98 | 0.98 | -0.01 |
| Yes | 0.02 | 0.02 | 0.01 | 0.02 | 0.01 | 0.04 | 0.02 | 0.02 | -0.03 | 0.01 | 0.02 | -0.05 | 0.02 | 0.02 | 0.01 |
| Heart |  |  |  |  |  |  |  |  |  |  |  |  |  |  |  |
| No | 0.97 | 0.95 | 0.14 | 0.97 | 0.95 | 0.11 | 0.97 | 0.95 | 0.10 | 0.97 | 0.97 | 0.00 | 0.97 | 0.97 | 0.00 |
| Yes | 0.03 | 0.05 | -0.14 | 0.03 | 0.05 | -0.11 | 0.03 | 0.05 | -0.10 | 0.03 | 0.03 | 0.00 | 0.03 | 0.03 | 0.00 |
| Osteoporosis |  |  |  |  |  |  |  |  |  |  |  |  |  |  |  |
| No | 0.76 | 0.79 | -0.07 | 0.75 | 0.76 | -0.02 | 0.76 | 0.77 | -0.03 | 0.77 | 0.78 | -0.02 | 0.75 | 0.74 | 0.03 |
| Yes | 0.24 | 0.21 | 0.07 | 0.25 | 0.24 | 0.02 | 0.24 | 0.23 | 0.03 | 0.23 | 0.22 | 0.02 | 0.25 | 0.26 | -0.03 |
| Cerebral |  |  |  |  |  |  |  |  |  |  |  |  |  |  |  |
| No | 0.98 | 0.94 | 0.29 | 0.98 | 0.94 | 0.26 | 0.98 | 0.94 | 0.24 | 0.98 | 0.98 | -0.01 | 0.98 | 0.98 | -0.01 |
| Yes | 0.02 | 0.06 | -0.29 | 0.02 | 0.06 | -0.26 | 0.02 | 0.06 | -0.24 | 0.02 | 0.02 | 0.01 | 0.02 | 0.02 | 0.01 |
| Comorbidity |  |  |  |  |  |  |  |  |  |  |  |  |  |  |  |
| No | 0.72 | 0.68 | 0.08 | 0.71 | 0.67 | 0.09 | 0.71 | 0.67 | 0.09 | 0.72 | 0.73 | -0.03 | 0.70 | 0.70 | 0.00 |
| Yes | 0.28 | 0.32 | -0.08 | 0.29 | 0.33 | -0.09 | 0.29 | 0.33 | -0.09 | 0.28 | 0.27 | 0.03 | 0.30 | 0.30 | 0.00 |
| Hospitalization frequency | 3.39 | 2.77 | 0.19 | 3.39 | 2.70 | 0.21 | 3.50 | 2.56 | 0.28 | 3.49 | 2.67 | 0.25 | 2.99 | 3.06 | -0.02 |

Local, local center pattern; National, National center pattern; SMD, Standardized mean difference.

# Supplementary Table 9. Five-year survival for sequentially matched patients and hazard ratio of all-cause mortality risk for local and national center patterns vs. intra-city pattern in sensitivity analysis.

| Comparison between intra-city pattern and local center pattern | | | | | |
| --- | --- | --- | --- | --- | --- |
|  | Demographic matched | Marriage matched | Cancer matched | Morbidity matched | Frequency matched |
| 5-year all-cause survival (%) |  |  |  |  |  |
| Intra-city | 65.1 | 66.7 | 64.8 | 66.0 | **64.0** |
| Local center | 69.4 | 69.1 | 68.5 | 67.7 | **68.0** |
| Difference in survival (pp., reference: intra-city) | 4.3 | 2.4 | 3.7 | 1.7 | **4.0** |
| Difference explained (pp.)^1^ |  | 1.9 | 1.3 | 2.0 | **2.3** |
| HR, (95%CI, reference: intra-city) | 0.83(0.74-0.93) | 0.89(0.79-1.00) | 0.85(0.76-0.96) | 0.92(0.82-1.03) | **0.86(0.76-0.96)** |
| P value | <0.01 | 0.05 | 0.01 | 0.16 | **0.01** |
| Comparison between intra-city pattern and national center pattern | | | | | |
|  | Demographic matched | Marriage matched | Cancer matched | Morbidity matched | Frequency matched |
| 5-year all-cause survival (%) |  |  |  |  |  |
| Intra-city | 69.1 | 68.3 | 66.3 | 66.8 | **66.7** |
| National center | 71.4 | 71.4 | 71.4 | 71.2 | **72.2** |
| Difference in survival (pp., reference: intra-city) | 2.3 | 3.1 | 5.1 | 4.4 | **5.5** |
| Difference explained (pp.)^1^ |  | 0.8 | 2.0 | 0.7 | **1.1** |
| HR, (95%CI, reference: intra-city) | 0.89(0.73-1.09) | 0.86(0.70-1.06) | 0.79(0.64-0.98) | 0.83(0.67-1.02) | **0.80(0.65-0.99)** |
| P value | 0.27 | 0.15 | 0.03 | 0.08 | **0.04** |

^1^: the survival difference explained by a set of matching variables was calculate as the absolute change in survival difference before and after adjusting for the corresponding set of variables. pp.: percentage point. HR: hazard ratio. Demographic variables included age, sex, first hospitalization year, residential city. Patients with any hospitalization records within the first six months (January 1, 2015 to May 31, 2015) of the study period were excluded in the sensitivity analysis. Marriage variables included demographic variables and marital status. Cancer variables included marriage variables and cancer types. Comorbidity variables included cancer variables and comorbidities including hypertension, diabetes, heart disease, osteoporosis, cerebral disease. Frequency variables included comorbidity variables and hospitalization frequencies (shown in bold). Intra-city indicated patients who never hospitalized outside the residential city. Local center indicated patients who never hospitalized outside the Shandong province but hospitalized outside the residential city at least once. National center indicated patients who hospitalized outside the Shandong province at least once.

# Supplementary Table 10. Five-year survival for sequentially matched patients and hazard ratio of all-cause mortality risk for local and national center patterns vs. intra-city pattern by healthcare resource and cancer type.

|  | Five most common cancers, intra-city vs. local center | | | | | Uncommon cancers, intra-city vs. local center | | | | |
| --- | --- | --- | --- | --- | --- | --- | --- | --- | --- | --- |
|  | Demographic matched | Marriage matched | Cancer matched | Morbidity matched | Frequency matched | Demographic matched | Marriage matched | Cancer matched | Morbidity matched | Frequency matched |
| 5-year all-cause survival (%) |  |  |  |  |  |  |  |  |  |  |
| Intra-city | 67.9 | 71.1 | 67.9 | 68.8 | 67.5 | 60.9 | 60.3 | 59.9 | 59.9 | 59.8 |
| Local center | 74.8 | 73.8 | 72.4 | 72.6 | 73.4 | 61.7 | 62.6 | 62.6 | 62.2 | 62.2 |
| Difference in survival (pp., reference: intra-city) | 6.9 | 2.7 | 4.5 | 3.8 | 5.9 | 0.8 | 2.3 | 2.7 | 2.3 | 2.4 |
| Difference explained (pp.)^a^ |  | 4.2 | 1.8 | 0.7 | 2.1 |  | 1.5 | 0.4 | 0.4 | 0.1 |
| HR, (95%CI, reference: intra-city) | 0.73(0.64-0.84) | 0.86(0.75-1) | 0.81(0.7-0.94) | 0.84(0.72-0.97) | 0.78(0.67-0.9) | 0.92(0.79-1.07) | 0.86(0.73-1) | 0.87(0.74-1.02) | 0.88(0.75-1.03) | 0.88(0.75-1.03) |
| P value | <0.01 | 0.05 | <0.01 | 0.02 | <0.01 | 0.27 | 0.05 | 0.08 | 0.1 | 0.1 |
|  | Five most common cancers, intra-city vs. national center | | | | | Uncommon cancers, intra-city vs. national center | | | | |
|  | Demographic matched | Marriage matched | Cancer matched | Morbidity matched | Frequency matched | Demographic matched | Marriage matched | Cancer matched | Morbidity matched | Frequency matched |
| 5-year all-cause survival (%) |  |  |  |  |  |  |  |  |  |  |
| Intra-city | 71.8 | 72.3 | 71.4 | 70.6 | 71.9 | 57.1 | 59.2 | 55.7 | 57.8 | 52.5 |
| National center | 72.6 | 72.7 | 71.8 | 71.7 | 73.1 | 62.8 | 61.3 | 62.8 | 64.8 | 62.6 |
| Difference in survival (pp., reference: intra-city) | 0.8 | 0.4 | 0.4 | 1.1 | 1.2 | 5.7 | 2.1 | 7.1 | 7.0 | 10.1 |
| Difference explained (pp.)^a^ |  | 0.4 | 0.0 | 0.7 | 0.1 |  | 3.6 | 5.0 | 0.1 | 3.1 |
| HR, (95%CI, reference: intra-city) | 0.91(0.7-1.19) | 0.93(0.71-1.2) | 0.94(0.72-1.23) | 0.91(0.7-1.18) | 0.88(0.67-1.16) | 0.8(0.63-1.02) | 0.89(0.69-1.15) | 0.78(0.6-1.01) | 0.76(0.58-0.99) | 0.72(0.55-0.94) |
| P value | 0.50 | 0.57 | 0.66 | 0.47 | 0.37 | 0.07 | 0.37 | 0.06 | 0.04 | 0.02 |
|  | Cities with hospital beds per capita more than average level, intra-city vs. local center | | | | | Cities with hospital beds per capita less than average level, intra-city vs. local center | | | | |
|  | Demographic matched | Marriage matched | Cancer matched | Morbidity matched | Frequency matched | Demographic matched | Marriage matched | Cancer matched | Morbidity matched | Frequency matched |
| 5-year all-cause survival (%) |  |  |  |  |  |  |  |  |  |  |
| Intra-city | 64.7 | 68.4 | 62.6 | 64.7 | 65.8 | 66.4 | 66.1 | 65 | 65.5 | 64.9 |
| Local center | 70.6 | 69.9 | 68.2 | 68.3 | 69.2 | 69.1 | 69.5 | 67.7 | 67.9 | 68.3 |
| Difference in survival (pp., reference: intra-city) | 5.9 | 1.5 | 5.6 | 3.6 | 3.4 | 2.7 | 3.4 | 2.7 | 2.4 | 3.4 |
| Difference explained (pp.)^a^ |  | 4.4 | 4.1 | 2.0 | 0.2 |  | 0.7 | 0.7 | 0.3 | 1.0 |
| HR, (95%CI, reference: intra-city) | 0.80(0.69-0.92) | 0.94(0.81-1.1) | 0.81(0.7-0.94) | 0.87(0.74-1.01) | 0.87(0.74-1.01) | 0.86(0.75-0.99) | 0.83(0.72-0.95) | 0.86(0.74-0.99) | 0.87(0.75-1.01) | 0.85(0.74-0.99) |
| P value | <0.01 | 0.46 | <0.01 | 0.06 | 0.07 | 0.03 | 0.01 | 0.04 | 0.06 | 0.03 |
|  | Cities with hospital beds per capita more than average level, intra-city vs. local center | | | | | Cities with hospital beds per capita less than average level, intra-city vs. local center | | | | |
|  | Demographic matched | Marriage matched | Cancer matched | Morbidity matched | Frequency matched | Demographic matched | Marriage matched | Cancer matched | Morbidity matched | Frequency matched |
| 5-year all-cause survival (%) |  |  |  |  |  |  |  |  |  |  |
| Intra-city | 70.5 | 69.1 | 67.0 | 67.0 | 65.3 | 66.5 | 66.2 | 63.1 | 65.3 | 66.5 |
| National center | 70.2 | 70.1 | 71.1 | 70.0 | 71.6 | 66.1 | 65.5 | 64.5 | 63.0 | 66.9 |
| Difference in survival (pp., reference: intra-city) | -0.3 | 1.0 | 4.1 | 3.0 | 6.3 | -0.4 | -0.7 | 1.4 | -2.3 | 0.4 |
| Difference explained (pp.)^a^ |  | 1.3 | 3.1 | 1.1 | 3.3 |  | 0.3 | 2.1 | 3.7 | 2.7 |
| HR, (95%CI, reference: intra-city) | 0.99(0.77-1.26) | 0.94(0.73-1.19) | 0.83(0.65-1.08) | 0.87(0.68-1.12) | 0.75(0.58-0.96) | 0.95(0.73-1.24) | 0.96(0.74-1.26) | 0.91(0.69-1.19) | 1(0.76-1.32) | 0.94(0.7-1.25) |
| P value | 0.91 | 0.59 | 0.16 | 0.29 | 0.03 | 0.71 | 0.77 | 0.48 | 0.99 | 0.67 |

^a^: the survival difference explained by a set of matching variables was calculate as the absolute change in survival difference before and after adjusting for the corresponding set of variables. pp.: percentage point. HR: hazard ratio. In the frequency-matching process, which was the final step in the sequential matching procedure, all matching variables were included: age, sex, year of first hospitalization, residential city, marital status, cancer types, comorbidities (including hypertension, diabetes, heart disease, osteoporosis, and cerebral disease), and hospitalization frequencies. Intra-city indicated patients who never hospitalized outside the residential city. Local center indicated patients who never hospitalized outside the Shandong province but hospitalized outside the residential city at least once. National center indicated patients who hospitalized outside the Shandong province at least once. Five most common cancers included lung, colorectum, stomach, breast, and thyroid cancer. Uncommon cancers were defined as other cancers except five common cancers. Above -average and below-average healthcare resource groups indicated cities with hospital beds per capita more and less than average level of all cities in Shandong province, respectively.
